# Antihypertensive drugs and brain function: mechanisms underlying therapeutically beneficial and harmful neuropsychiatric effects

**DOI:** 10.1093/cvr/cvac110

**Published:** 2022-07-28

**Authors:** Carla Carnovale, Cristiana Perrotta, Sara Baldelli, Dario Cattaneo, Cristina Montrasio, Silvia S Barbieri, Giulio Pompilio, Chiara Vantaggiato, Emilio Clementi, Marco Pozzi

**Affiliations:** Unit of Clinical Pharmacology, Department of Biomedical and Clinical Sciences (DIBIC), ASST Fatebenefratelli-Sacco University Hospital, Università degli Studi di Milano, Via GB Grassi 74, 20157 Milano, Italy; Unit of Clinical Pharmacology, Department of Biomedical and Clinical Sciences (DIBIC), ASST Fatebenefratelli-Sacco University Hospital, Università degli Studi di Milano, Via GB Grassi 74, 20157 Milano, Italy; Unit of Clinical Pharmacology, ASST Fatebenefratelli-Sacco University Hospital, 20157 Milano, Italy; Unit of Clinical Pharmacology, ASST Fatebenefratelli-Sacco University Hospital, 20157 Milano, Italy; Unit of Clinical Pharmacology, ASST Fatebenefratelli-Sacco University Hospital, 20157 Milano, Italy; Unit of Brain-Heart Axis: Cellular and Molecular Mechanisms – Centro Cardiologico Monzino IRCCS, 20138 Milano, Italy; Unit of Vascular Biology and Regenerative Medicine – Centro Cardiologico Monzino IRCCS, 20138 Milan, Italy; Department of Biomedical, Surgical and Dental Sciences, Università degli Studi di Milano, 20122 Milan, Italy; Scientific Institute IRCCS Eugenio Medea, 23842 Bosisio Parini (LC), Italy; Unit of Clinical Pharmacology, Department of Biomedical and Clinical Sciences (DIBIC), ASST Fatebenefratelli-Sacco University Hospital, Università degli Studi di Milano, Via GB Grassi 74, 20157 Milano, Italy; Scientific Institute IRCCS Eugenio Medea, 23842 Bosisio Parini (LC), Italy; Scientific Institute IRCCS Eugenio Medea, 23842 Bosisio Parini (LC), Italy

**Keywords:** Antihypertensive drugs, Neuropsychiatric disorders, Personalized medicine

## Abstract

A bidirectional relationship exists between hypertension and psychiatric disorders, including unipolar and bipolar depression, anxiety, post-traumatic stress disorder (PTSD), psychosis, schizophrenia, mania, and dementia/cognitive decline. Repurposing of antihypertensive drugs to treat mental disorders is thus being explored. A systematic knowledge of the mechanisms of action and clinical consequences of the use of antihypertensive agents on neuropsychiatric functions has not been achieved yet. In this article, we review the putative role of antihypertensive agents in psychiatric disorders, discuss the targets and mechanisms of action, and examine how and to what extent specific drug classes/molecules may trigger, worsen, or mitigate psychiatric symptoms. In addition, we review pharmacokinetics (brain penetration of drugs) and pharmacogenetics data that add important information to assess risks and benefits of antihypertensive drugs in neuropsychiatric settings.

The scientific literature shows robust evidence of a positive effect of α1 blockers on PTSD symptoms, nightmares and sleep quality, α2 agonists on core symptoms, executive function, and quality of life in Attention-Deficit/Hyperactivity Disorder, PTSD, Tourette’s syndrome, and β blockers on anxiety, aggression, working memory, and social communication. Renin-angiotensin system modulators exert protective effects on cognition, depression, and anxiety, and the loop diuretic bumetanide reduced the core symptoms of autism in a subset of patients. There is no evidence of clear benefits of calcium channel blockers in mood disorders in the scientific literature. These findings are mainly from preclinical studies; clinical data are still insufficient or of anecdotal nature and seldom systematic. The information herewith provided can support a better therapeutic approach to hypertension, tailored to patients with, or with high susceptibility to, psychiatric illness. It may prompt clinical studies exploring the potential benefit of antihypertensive drugs in selected patients with neuropsychiatric comorbidities that include outcomes of neuropsychiatric interest and specifically assess undesirable effects or interactions.

## Graphical Abstract

**Graphical abstract:**
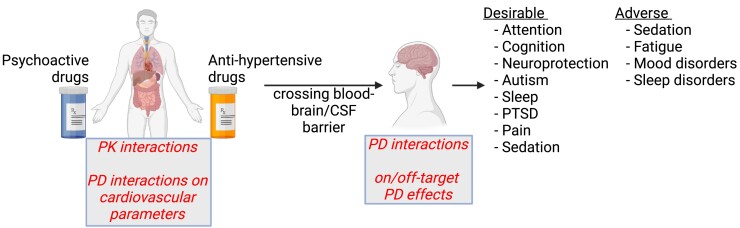

## 1. Introduction

The mind–heart–body relationship, specifically the connection between mental disorders and cardiovascular diseases, has received considerable attention in the last decades. Consistent evidence indicates that patients suffering of both cardiovascular diseases and psychiatric disorders (e.g. depression, anxiety, post-traumatic stress, schizophrenia, dementia, and cognitive decline) have a lower quality of life and are at higher risk of mortality.^1–5^ Hypertension not only is a major risk factor for cardiovascular diseases but also is related to mental health problems such as anxiety and depression. Hypertensive patients are more likely to have a recorded diagnosis of mental disorders, and hypertension increases the severity of psychological distress. Conversely, prospective studies showed that psychiatric disorders are independent risk factors for hypertension. Mental disorders can cause continuous activation of the sympathetic nervous system and dysfunction of the hypothalamus-pituitary-adrenaline axis, thus increasing vascular tone and blood pressure and leading to pathological hypertension disorders.^6^ In conclusion, a clinically significant bidirectional relationship between these two disorders exists,^7–9^ leading to a vicious cycle that contributes to worsen their outcome. Proper psychiatric and hypertensive management may be crucial to prevent complications in these patients.

Based on this knowledge, in recent years the possibility of repurposing antihypertensive drugs to treat mental disorders has been explored: notable examples include diuretics (bumetanide) for autism and epilepsy,^10^ and AT1R blockers (ARBs) and calcium channel blockers (CCBs) for Alzheimer’s dementia.^11^ From a clinical perspective, it is important to clarify how and to what extent antihypertensive drugs would exert neuropsychiatric effects, both desirable and adverse, also in view of managing combined therapies. In this review, we provide an overview of essential aspects of the pharmacokinetics and pharmacogenetics of the different pharmacological classes of antihypertensive drugs. For each class, we describe the receptors and molecular mechanisms through which antihypertensive drugs generate neuropsychiatric effects in preclinical models, highlighting those that, in our opinion, are desirable and potentially exploitable in therapy. We then discuss drug-drug interactions with commonly prescribed psychiatric drugs, focussing only on psychiatric aspects, while metabolic issues are reviewed elsewhere.^12,13^ Lastly, we report the results of a systematic review that we performed on data from clinical studies included over the last 20 years in the most popular bibliographic database in health and medical sciences, PUBMED/Medical Literature Analysis and Retrieval System Online (MEDLINE), Excerpta Medica database (EMBASE), and Psycinfo that provides systematic coverage of literature in the field of psychology. In this context, we describe the psychiatric effects of antihypertensive drugs and, where relevant, the pharmacological aspects that may influence such effects.

This article aims to stimulate future clinical research studies and promote a conscious tailored therapeutic approach to hypertension in patients with, or with high susceptibility to, psychiatric illness.

## 2. Central nervous system penetration of anti-hypertensive drugs

The ability of anti-hypertensive drugs to exert their potential neurological effects is linked to the capability of these compounds to cross the blood–brain barrier (BBB) and/or the blood cerebrospinal fluid barrier (BCSFB) and reach their therapeutic targets in the central nervous system (CNS) in sufficient concentrations.^14^

**Figure 1:**
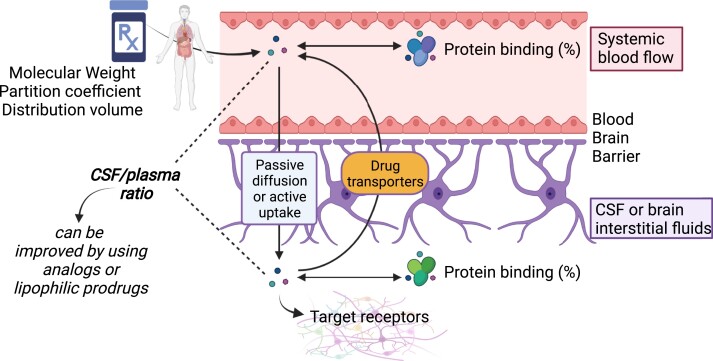
Brain penetration of anti-hypertensive and other drugs. Brain penetration of anti-hypertensive drugs is regulated by some intrinsic chemical properties, including molecular weight, partition coefficient, and distribution volume: these regulate the overall permeability of drugs through biological membranes. Once drugs are absorbed and distributed in the blood flow, they are bound by plasma proteins. The unbound fraction can cross the BBB either passively or actively, and it is often sent back to the blood flow by drug transporters. Once in the cerebrospinal fluid (CSF) or brain interstitial fluids, again drugs can be bound. Unbound fractions in the brain can actually bind their receptors and produce effects. Created with BioRender.com.

Drugs can pass the BBB and/or the BCSFB either by passive diffusion or by active uptake through specific trans-membrane protein transporters (carrier-mediated endocytosis) (*Figure 1*). Most small molecule drugs with high lipophilicity (molecular weight <400–600 Dalton; logarithm of the octanol/water partition coefficient, i.e. logP >1) are able to enter the brain by trans-cellular passive diffusion through the lipid membranes. However, the extent of brain penetration for these drugs depends also on the protein binding in blood and brain. Only the unionized, unbound drug concentration at the site of action leads to pharmacological activity; therefore, brain entry by passive diffusion may be significantly restricted for molecules with a high protein binding (>80%).

Furthermore, efflux transporters such as P-glycoprotein (P-gp), organic anionic transporters (OATPs), and breast cancer resistance protein (BCRP) protect the brain from penetration of harmful substances. Therefore, if a drug is substrate of these efflux transporters, it is less likely to penetrate into the brain (*Table 1*).

**Table 1:** Physico-chemical and pharmacokinetic features of the main anti-hypertensives from different classes

	Molecular weight (Da)	Log p	Vd (L/kg)	Protein binding (%)	CSF/plasma ratio, %	Drug transporters
ACEIs						
Captopril	217	0.3	0.7	30%	Not available	Inhibitor of P-gp
Enalapril	376	2.5	2.0	50%	Not available	Substrate of OATP1 and MRP2
Lisinopril	405	−1.3	1.8	<1%	Not available	
Trandolapril	431	1.9	0.3	80%	Not available	
Ramipril	416	2.9	1.2	70%	2%	
CCB						
Amlodipine	409	3	21.4	98%	Not available	Substrate and inhibitor of P-g
Verapamil	455	1.8	3.8	88%	7%	Substrate and strong inhibitor of P-gp
Nifedipine	346	2.2	13	95%	Not available	Inhibitor of P-gp
Felodipine	383	3.8	10	99%	Not available	Substrate of P-gp
Diltiazem	414	3	5.3	75%	10%	Substrate and inhibitor of P-gp
Beta-blockers						
Bisoprolol	325	2.2	3.5	30%	55%	Inhibitor of P-gp
Metoprolol	267	2.1	4.0	10%	43%	
Atenolol	266	0.2	0.7	3%	10%	
Propanolol	259	3.5	4.0	90%	5%	Substrate and inducer of P-gp
Carvedilol	406	3.8	1.6	95%	Not available	Strong inhibitor of P-gp
Diuretics						
Bumetanide	364	2.1	0.44	96%	2%	Substrate of OATP1 and MRP4
Furosemide	331	1.8	0.14	99%	Not available	Substrate of MRP4 and BCRP
Torasemide	348	3.4	0.2	99%	Not available	Substrate of SLCO1B1
Chlorthalidone	339	0.9	3.9	75%	Not available	
Hydrochlorothiazide	298	−0.1	2.0	58%	4%	Substrate of MRP4

CCB, calcium channel blockers; P-gp, P-glycoprotein.

**Table 2:** Binding profile of antihypertensive drugs at selected receptors of neuropsychiatric interest

Class	Drugs\receptors	NE β1	NE β2	NE β3	NE α1A	NE α1B	NE α1D	NE α2A	NE α2B	NE α2C	5-HT1A	5-HT1B	5-HT2B	5-HT2C	DRD2	VMAT1	VMAT2	AcChE	MAOA	LAAD	SLC12A3	SCNN1A	Carb.Anhyd	Kir6.1	Kir6.2	InsP3R	MinCortR	AndrogenR	L-type VGCC	ACE	AT1R
**Selective β blocker (β1>β2)**	Acebutolol	ISA																													
	Atenolol																														
	Betaxolol	ISA	ISA																												
	Bisoprolol																														
	Celiprolol	ISA	ISA					ISA																							
	Esmolol																														
	Metoprolol																														
	Nebivolol																														
**Nonselective β blocker (β1≤β2)**	Nadolol																														
	Oxprenolol	ISA	ISA																												
	Penbutolol	ISA	ISA																												
	Pindolol	ISA	ISA	ISA																											
	Propranolol																														
	Sotalol																														
	Tertatolol																														
	Timolol																														
**α1-β blockers**	Carvedilol																														
	Labetalol	ISA	ISA																												
**α1 blockers**	Doxazosin																														
	Prazosin																														
	Alfuzosin																														
	Terazosin																														
**α2 agonists**	Clonidine																														
	Guanfacine																														
	Guanabenz																														
	Methyldopa							AM	**Ki?**	**Ki?**										**Ki?**											
**Thiazide diuretics**	Chlorthalidone																														
	Chlorothiazide																														
	Hydrochlorothiazide																														
	Indapamide																														
	Metolazone																														
**K sparing diuretics**	Amiloride																														
	Spironolactone																														
	Triamterene																														
**Loop diuretics**	Furosemide																														
	Bumetanide																														
**Others**	Reserpine																														
	Hydralazine																									**Ki?**					
	Minoxidil																							**Ki?**	**Ki?**						
**Calcium Channel Blockers**	Isradipine																														
	Nicardipine																														
	Nifedipine																														
	Nimodipine																														
	Diltiazem																														
	Verapamil																														
**ACEIs**	Captropril																														
	Lisinopril																														
	Perindopril																														
**AT1R blockers**	Candesartan																														
	Irbesartan																														
	Losartan																														
	Telmisartan																														
	Valsartan																														

Colours reported in each cell indicate the pKi for the specific binding between drugs (left) and receptors (top): red = pKi above 9; orange = pKi 8-9; yellow = pKi 7-8; green = pKi 6-7; blue = pKi 5-6; violet = pKi 4-5; gray = binding confirmed, but pKi not known. Only receptors for which at least one drug shows at least pKi >6 are shown.

AM, active metabolite; ISA, intrinsic sympathomimetic activity. Sources for parameters are the PDSP Database [Science Netwatch, 28 January 2000; 287 (5453)] and the Binding DB [Gilson, M.K., Liu, T., Baitaluk, M., Nicola, G., Hwang, L., and Chong, J. BindingDB in 2015: A public database for medicinal chemistry, computational chemistry and systems pharmacology Nucleic Acids Research 44:D1045-D1063 (2016)].

*Table 1* provides the main physico-chemical and pharmacokinetic features of some antihypertensive drugs, which can be used to predict their ability to cross the brain barriers. This table is not intended to be exhaustive of all the antihypertensive drugs on the market; it aims to show that the capacity to penetrate the CNS can vary for molecules belonging to the same therapeutic category based on their chemical, physical, and pharmacokinetic characteristics. All antihypertensive drugs are small molecules and, therefore, potential candidates for passive diffusion. However, even within the same drug class, important differences exist in the lipophilicity of the different compounds. For instance, among the ACE inhibitors (ACEIs), lisinopril has low lipophilicity and thus, theoretically, less propensity to cross the brain barriers than ramipril, which has high lipophilicity. The picture is further complicated by key differences in the protein binding, with lisinopril largely circulating as a free drug and ramipril been mostly bound to plasma proteins. Based on these considerations, lisinopril could entry the brain to a higher extent than ramipril. One of the most evident limitations in *Table 1* is the lack of data on CNS penetration for most of the molecules. In the only study published so far for ACEIs,^15^ it was shown that cerebrospinal fluid (CSF) reached 4.1% (interquartile ranges 2.5–5%) of total serum concentrations for hydrochlorothiazide and 2.3% (1.7–5.7%) for ramiprilat, corresponding to about 11.3% and 5.5% of respective unbound serum concentrations. Since the CSF levels of these agents, both free and bound, were much lower than the corresponding concentrations in serum, the Authors concluded that the observed CNS adverse events were unlikely mediated primarily via direct effects of these drugs in the brain. These data suggest that the CNS concentrations reached by these antihypertensive drugs at therapeutic dosages are likely too low to carry out beneficial actions at a central level. Similarly, also for CCBs and diuretics, with a trend for higher CSF/plasma ratio reported for verapamil (7%) and diltiazem (10%) compared with bumetanide (2%).^16–18^ Conversely, a significantly higher penetration has been reported for β blockers, ranging from 5% for propranolol up to 55% for bisoprolol.^15^ This difference is presumably driven by a combination of high lipophilicity and low protein binding, with the exception of propranolol (*Table 1*).

## 3. Adrenergic modulators

### 3.1 Adrenergic receptors in the brain

**Figure 2:**
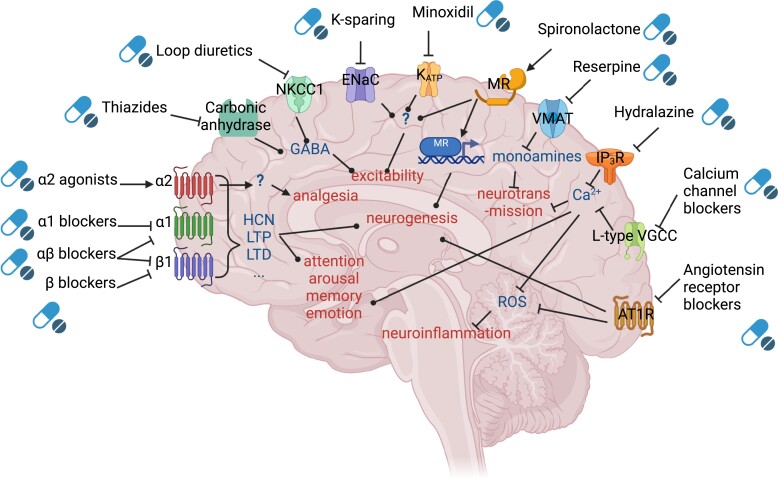
Main mechanisms of action of anti-hypertensive drugs in the brain. For each drug class, we reported on the brain surface the main molecular target in the brain; downstream, we reported the intermediate mechanisms of action; terminally, we reported the higher-level final mechanisms of action. The aspects and localizations of receptors and mechanisms are purely graphical and do not indicate molecular structures or anatomical localizations. Sharp-headed arrows indicate stimulation; flat-headed arrows indicate inhibition; circular-headed arrows indicate modulation. Created with BioRender.com.

Several antihypertensive drugs act through antagonism at β or α1, or agonism at α2 adrenergic receptors (*Table 2* and *Figure 2*). These adrenergic receptors are integrated in the Cortico-Striato-Thalamo-Cortical (CSTC) neurocircuitry,^19^ where they regulate arousal, attention, anxiety, and emotional trauma through the norepinephrine signalling (among other neurotransmission systems). Although the adrenal medulla can release norepinephrine in the bloodstream, the locus coeruleus (LC) is the primary source of norepinephrine for the brain, since it innervates several areas of the cortex and can efficiently alter the CSTC function. The amount of released norepinephrine is proportional to the amount and intensity of stimuli received from sensory organs, and correlates directly with the overall activity of the CSTC. The norepinephrine receptors present in the CSTC circuit can be put in a pharmacologically relevant order by decreasing affinity to norepinephrine, starting with the α2C presynaptic autoreceptors, α2A post-synaptic receptors and presynaptic hetero/autoreceptors, α1 post-synaptic receptors, and ending with β post-synaptic receptors: their sequential activation produces different cognitive effects, as detailed below.

During sleep, norepinephrine release from the LC is almost absent. During waking in the absence of stimuli, at the lowest norepinephrine concentrations, the presynaptic autoreceptors α2C on the LC noradrenergic neurones inhibit norepinephrine release towards the prefrontal cortex (PFC). As a consequence, the CSTC is in a resting condition. In the presence of external stimuli, low tonic norepinephrine release from the LC maintains wakefulness and allows the allocation of attention to distinct stimuli. In this context, the α2A post-synaptic receptors on the pyramidal neurones in the PFC lead to hyperpolarization-activated cyclic nucleotide-gated (HCN) channels closure and produce attention focussing. At the same time, the α2A presynaptic heteroreceptors on the serotoninergic neurones of the amygdala prevent emotional interferences, and the α2A presynaptic autoreceptors in the LC prevent further rises in norepinephrine release, thus maintaining a tonic signalling. In these circumstances, the CSTC directs attention towards salient stimuli.

Potent or repetitive sensorial stimuli produce a more intense and phasic norepinephrine signalling (rapid subsequent intense bursts), which engages α1 receptors. α1B receptors stimulation on PFC neurones^20,21^ activates protein kinase C (PKC), which consecutively opens potassium channels SK and K_v_7.^22,23^ This leads to a reorientation of attention, which is not focussed anymore, but becomes diffuse and spatial (i.e. to look out for danger), while reducing the ability of the PFC to focus on specific tasks.^24^ In this situation, corresponding to mild/acute stress, the CSTC engages memory formation, allowing emotional components to be included with the processing of sensory stimuli. α1A and α1D receptors in the hippocampus facilitate emotional memory formation.^25^

When very stressful conditions further enhance the noradrenergic signalling from LC, β adrenergic receptors are engaged and increase cAMP levels, thus triggering signalling and effects that are opposite to those of the α2A receptors in the PFC. The activation of β receptors in the PFC increases spatial arousal at the expense of attention and cognitive processing.^26^ In addition, it stimulates serotoninergic neurones in the amygdala, favouring the adaptation to stress and promoting the formation of emotional memory.^27^ In parallel, β receptors induce memory consolidation in the hippocampus,^28,29^ with a specific preference for emotional events.^30,31^ This mechanism is also involved in the development of emotional trauma and post-traumatic stress.

Other important and complex noradrenergic functions include the direct and indirect (via melatonin) regulation of sleep^32–34^ and analgesia mediated by α2 receptors.^35^

### 3.2 Preclinical evidence for neuropsychiatric effects of adrenergic antihypertensive drugs and pharmacodynamic interactions with neuropsychiatric drugs

Based on their lipophilicity, noradrenergic antihypertensive drugs have different propensity to enter the brain and exert psychoactive effects (*Figure 1*). Attention and working memory can be enhanced by α2 agonists (including clonidine, guanfacine, methyldopa, guanabenz), α1 blockers (primarily the lipophilic prazosin), β blockers (mostly the lipophilic ones, without regard to β selectivity), and even more by α1/β blockers (carvedilol and labetalol). A possible side effect of these drugs is fatigue.^36^ Further effects of α2 agonists include the reduction of anaesthetic and opioid dose requirements in the context of surgery.^37^

Regarding emotional associations and memory area, α1 blockers can prevent the formation of fear conditioning and stressful memories, but cannot revert them.^38^ β1 blockers are acutely anxiolytic; however, they have delayed depressant effects, possibly due to the impairment of long-term potentiation. They induce symptoms of fatigue and lethargy, putatively due to their inhibitory activity on neuronal plasticity; they can alter sleep and cause nightmares in unclear ways; in rare cases, they can cause hallucination, possibly by disrupting dopaminergic signals.^28,29^ Moreover, β blockers reduce melatonin levels.^39^

Concerning the interactions of antihypertensive drugs with neuropsychiatric drugs, secondary effect of α2 antagonism from α1 blockers can improve antidepressant action by avoiding some adverse effects that are due to norepinephrine and serotonin excess, in particular short-term anxiety and gastrointestinal disturbances.^40^ Conversely, α2 antagonism can reduce the efficacy of several antipsychotics that work on the negative symptoms of schizophrenia and extrapyramidal disorders via serotonin increase. Lastly, α2 agonists can increase the adverse effects of antidepressants, while they can reduce the adverse effects and increase the therapeutic effects of antipsychotics.

α1 antagonism is a core mechanism by which antipsychotics reduce excessive arousal and reactive/aggressive behaviours; therefore, α1 blockers may improve the effectiveness of antipsychotics on soothing and sedation.

### 3.3 Clinical evidence for neuropsychiatric effects of adrenergic antihypertensive drugs and pharmacogenetic considerations

#### 3.3.1 α2 agonists

Most of the recent evidence on the role of α2 agonists in psychiatry is on the rehabilitation treatment of Attention-Deficit/Hyperactivity Disorder (ADHD) in children.^41^ Based on several large-scale randomized controlled trials (RCTs), the Food and Drug Administration (FDA) approved the extended-release formulations of clonidine and guanfacine, to treat children and adolescents aged 6– 17 years with ADHD. More recently, these drugs have been tested to treat a variety of psychiatric conditions beyond their approved indication. The main characteristics of studies reporting beneficial vs harmful effects of each antihypertensive drug class in the neuropsychiatric clinic are detailed in the Supplementary material online, *Table S1*. In adult ADHD patients, 3 RCTs showed that guanfacine produced clinically relevant improvements in core symptoms, executive function, and quality of life.^42–44^ No data are available yet for clonidine in adults with ADHD.^45^

Clonidine and guanfacine showed other promising neuropsychiatric effects; however, these were reported in non-controlled studies and require verification in randomized blinded trials.

Regarding ADHD comorbidities, clonidine extended-release significantly improved symptoms of oppositional defiant disorder, conduct disorder, insomnia,^46,47^ Tourette’s syndrome, or tics in children with ADHD treated with stimulants.^48^ Transdermal clonidine was used successfully to relieve anxiety and post-traumatic stress disorder (PTSD) symptoms in comorbidity with ADHD.^49–52^ Guanfacine extended-release reduced intrusive thoughts and hyperarousal in children with PTSD.^53^

Other off-label use of clonidine includes the management of obsessive-compulsive disorder (OCD) symptoms^54^ and clinically relevant sleep problems in paediatric patients affected by neurodevelopmental disorders.^55^ Clonidine has been also successfully used in monotherapy for hallucinations in adults with schizophrenia,^56–58^ although its mechanism of antipsychotic effect remains hypothetical.

The most common psychiatric side effects due to the use of α2 agonists are usually dose-related and reversible, and include dizziness, drowsiness, somnolence, and other depressive symptoms.^59,60^ Recently, a case of manic reaction in a child induced by guanfacine-extended release (up to 2 mg/day for 12 days) was reported;^59^ resolution occurred over 7 weeks following discontinuation of the α2 agonist. Clinical evidence on neuropsychiatric effects of antihypertensive drugs is briefly summarized in *Table 3*.

**Table 3:** Beneficial vs harmful effects of adrenergic antihypertensive drugs in the neuropsychiatric clinic

Antihypertensive drug class	Possible therapeutic role (the investigated drug/class)	Harmful effects (the investigated drug/class)
*Alpha 2 agonists* ^a^	Positive effects on: core symptoms, executive function and quality of life in adults with ADHD (Guanfacine) ^42–44^ intrusive thoughts and hyperarousal in children with PTSD (Guanfacine)^53^ oppositional defiant disorder, conduct disorder, insomnia, PTSD, Tourette’s syndrome or tics in children with ADHD treated with stimulants (Clonidine)^46–53^ hallucinations in adults with schizophraenia (Clonidine, in monotherapy)^56–58^ obsessive-compulsive disorder symptoms (Clonidine).^54^	Usually dose-related and reversible: dizziness, drowsiness, somnolence, and other depressive symptoms (All).^59,60^ Manic reaction (guanfacine-extended release, up to 2 mg/day for 12 days).^59^
*Alpha 1 blockers*	Positive effects on: PTSD symptoms, nightmares and sleep quality and content, both in adults^61–66^ and children^67^ (Prazosin) drug dreams in patients with substance use disorder (Prazosin).^68,69^	Not reported.
*Beta blockers*	Positive effects on: anxiety in patients suffering from anxiety disorders (Propanolol)^70^ aggression, verbal problem-solving/semantic fluency, working memory, social communication, in patients with schizophraenia, dementia, ASD (Particularly propanolol).^71–75^	Sleep disorders up to insomnia, nightmares, visual hallucinations, delirium or psychosis (All).^76–85^
*Diuretics*	Positive effects on: ASD symptoms, in a limited subgroup of children who have a specific deficit in GABAergic circuits (Bumetanide)^86–91^ CARS, ADOS, ABC, RDEG and RRB scores in a 10-year-old Fragile X boy (Bumetanide)^92^ schizophraenia symptoms (Bumetanide, diazoxide).^93,94^	No clear neuropsychiatric effect would be expected of bumetanide.
*CCBs*	Positive effects on: acute bipolar mania (All, particularly verapamil)^95,96^ verbal memory, attention dysfunction and functional capacity in patients with schizophraenia (Isradipine)^97,98^ cognitive function in elderly hypertensive people (All).^99,100^	Dizziness, difficulty sleeping and decreased energy in symptomatically-stable patients with schizophraenia (Isradipine).^97^ Delirium in a 63-year-old man with ischaemic stroke (Amlodipine).^b^
*ACEIs*	Positive effects on: depressed mood when compared with patients taking other antihypertensive drugs (All)^99,101–103^ mental health outcomes (overall quality of life, positive wellbeing, mental and anxiety domains of quality of life) (All)^104^ cognitive function in adult patients affected by cardiovascular disease (All).^b^	Visual hallucinations, paranoid delusions, confusion, disorientation, anxiety (Quinalapril).^b^ A higher risk of major psychiatric disorders, compared to ARB users (adjusted HR = 1.07; 95% CI = 1.02, 1.13) (All).^b^
*ARBs*	Positive effects on: schizophraenia symptoms (Telmisartan)^105,106^ depression and anxiety and functional statuses (Valsartan)^b^ cognitive function in older patients with hypertension (Candesartan).^107,108^	Risk for suicide (All).^109^

ABC, aberrant behaviour checklist; ADOS, autism diagnostic observation schedule; ARBs, angiotensin II type 1 receptor; ASD, autism spectrum disorder; CARS, Childhood Autism Rating Scale; GABA, gamma-aminobutyric acid; HR, hazard ratio; PTSD, post-traumatic stress disorder; RDEG, regulation disorder evaluation grid; RRB, repetitive and restrictive behaviour.

Clonidine and guanfacine are approved treatment for ADHD in children, long-acting formulations only.

The detailed list of references is reported in the Supplementary material online, *Table S1*.

#### 3.3.2 α1 blockers

A large amount of literature is available regarding the use of prazosin for sleep disturbances, specifically in the context of PTSD (see Supplementary material online, *Table S1*). The currently available systematic reviews and meta-analyses addressing adults have found predominantly positive findings on overall PTSD symptoms, nightmares, and sleep quality and content, supporting the use of prazosin as a good pharmacological option to treat PTSD.^61–65^ However, prazosin was not effective in improving sleep disorders in patients with PTSD and alcohol dependence.^110^ Although prazosin seems to be very promising in adults with PTSD, no firm conclusions should be drawn, due to the high methodological heterogeneity among studies on prazosin and PTSD, as well as the limited number of available trials.

A systematic review showed that prazosin may also be promising for PTSD-related nightmares in children and adolescents;^66^ however, only sporadic case reports are currently available, supporting the need for well-designed placebo-controlled trials. Preliminary evidence supports the use of prazosin to treat ‘drug dreams’ in patients with substance use disorder.^68,69^ Its mechanism of action seems to involve the decrease in noradrenaline effects at α1 adrenoreceptors, but is not well-characterized, particularly in the adolescent population.

Furthermore, preliminary studies have shown the potential efficacy of the α1-adrenergic antagonist doxazosin in the field of substance use disorder.^111,112^ In these studies, doxazosin reduced cocaine use, presumably by modulating norepinephrine-mediated adrenergic effects and/or altering the balance of dopamine and norepinephrine.^71^A double-blind, randomized, placebo-controlled trial^113^ found that cocaine use rates were influenced by the rs1611115 (c.-979T > C) polymorphism in the dopamine β-hydroxylase gene DβH, which codifies for the enzyme converting dopamine to norepinephrine). Cocaine use rates were lower in subjects with the T-allele (CT/TT) than in those with the CC genotype, with the T-allele associated with lower DβH and norepinephrine levels. The subsequent lower activation of α1 and β adrenergic receptors may result in reduced formation of emotional memory and reduced addiction mechanisms based on dopamine.

Another potential pharmacogenetic marker for substance use disorder was described in a 12-week placebo controlled clinical trial, focussed on the genetic variant rs2236554 (c.*129A > T) located in the intracellular domain of the α1 adrenoreceptor subtype D (ADRA1D) gene. In this trial, the T-allele carriers had a greater reduction of cocaine use after treatment with doxazosin.^114^

#### 3.3.3 β blockers

According to specific properties related to their lipophilicity, intrinsic sympathomimetic activity, and cardioselectivity, β blockers may exert different therapeutic and adverse effects on the CNS.^115^ It is well known that β blockers, especially propranolol and metoprolol, are associated with sleep disorders up to insomnia, nightmares, visual hallucinations, delirium or psychosis, even when they are used at low dose.^76–85^

Propranolol, the most investigated β blocker, showed potential for benefits in aggression, verbal problem-solving/semantic fluency,^71,72^ working memory,^73^ and social communication^74,75^ in case series and single-dose studies on patients with a variety of neuropsychiatric conditions, such as schizophrenia, dementia, autism spectrum disorders (ASD), or behavioural disorders (see Supplementary material online, *Table S1*). A recent meta-analysis of RCTs found no evidence for effects of propranolol on PTSD symptom severity.^70^

Despite the wide range of potential psychiatric applications for β blockers, positive evidence is currently most robust for treating symptoms of social anxiety; further potential roles for β blockers include panic disorder, aggression in patients with psychosis, acquired brain injury or intellectual disability, and cognitive protection in patients with recent stroke (see Supplementary material online, *Table S1*).

RCTs suggest that some β-blockers may also have antidepressants effects.^116^ Although more recent observational studies have challenged the association between β blocker therapy and increased risk of depression,^117,118^ others have not.^119^ Pharmacogenetic considerations tried to explain this discrepancy. Two common single nucleotide polymorphisms (SNPs) in linkage disequilibrium on the adrenergic β1 receptor (ADRB1) gene, p.Ser49Gly (rs1801252) and p.Gly389Arg (rs1801253), differentially affect blood pressure response to β-blocker therapy.^120,121^ Noteworthy, the rs1801253 polymorphism of the β1 adrenergic receptor was associated also with a better response to antidepressant treatment in patients with major depression.^122,123^ Furthermore, a study with homozygous 389Arg subjects examined the relationship between ADRB1 genetic variability and differences in systolic blood pressure and heart rate consequent to the use of SSRIs with high adrenergic β receptor affinity (‘beta-blocking’ SSRIs paroxetine and fluoxetine).^124^ The study subjects receiving paroxetine and fluoxetine had significantly lower systolic blood pressure and heart rate than those receiving other SSRIs.^125^ This evidence supports that ADRB1 genetic variability may also influence the effects of some antidepressants.

### 3.4 Adrenergic modulators—summary

The effects of adrenergic modulators depend on the receptor engaged in the brain. α1 antagonists reduce fear-related memories and enhance cognition even under stressful conditions; clinical data are however currently anecdotal and systematic research is needed to better elucidate their beneficial in treating patients with PTSD symptoms and substance use disorder. α2 agonists promote cognitive functions and potentiate antipsychotic effects, while increasing the adverse effects of antidepressants. α2 antagonists reduce the adverse effects of antidepressants, but decrease the efficacy of antipsychotics. Lastly, β antagonists, which are anxiolytic, induce symptoms of depression, including fatigue and lethargy, and sleep disruption. As most evidence on the role of β blockers comes from case reports and observational studies, rigorous studies investigating their benefits are needed.

## 4. Diuretics

### 4.1 Receptors for diuretics in the brain

Diuretic drugs act on molecular targets that are class-specific or even drug-specific. Diuretics alter the electrolyte content and, consequently, the excitability of neurones, thus producing changes in neurotransmission and potential neuropsychiatric effects. Diuretics have very different neuropsychiatric profiles depending on which receptor they engage.

Thiazide diuretics act predominantly by inhibiting sodium/chloride co-transporters; some of them can also inhibit the activity of several carbonic anhydrase (CA) subtypes (*Figure 2*). Sodium/chloride co-transporters participate in regulating the concentration of electrolytes throughout the body; they are crucial for neuronal activity and for the pharmacokinetics of any drug.

Among potassium sparing diuretics, amiloride and triamterene have their main target in the epithelial sodium channel (ENaC) (*Figure 2*), which is in the same superfamily as acid sensing ion channels (ASICs). ENaCs are ubiquitous, while ASICs are enriched in neurones and have important roles still under investigation.^126^

Spironolactone is a highly lipophilic mineralocorticoid antagonist with minor action on the androgen receptor, and a much less potent antagonist of the oestrogen and glucocorticoid receptors. The loop diuretics furosemide and bumetanide act by inhibiting the sodium-potassium-chloride co-transporter NKCC2; they also target its isoform NKCC1, expressed in the juvenile brain, which becomes increasingly silenced with neuronal development.^127^ As long as NKCC1 expression is high, the chloride electrochemical potential inside neurones is high. Consequently, GABA-triggered chloride currents flow inside-out and GABA acts as an excitatory neurotransmitter. This mechanism is considered important for brain synchronization and synapse wiring during brain development stages. Later, the electrochemical potential of chloride generates by the potassium-chloride co-transporter KCC2, which orients GABA-triggered chloride currents outside-in and allows the inhibitory activity of GABA that is typical of adult neurones.^128^

### 4.2 Preclinical evidence for neuropsychiatric effects of diuretic antihypertensive drugs and pharmacodynamic interactions with neuropsychiatric drugs

All diuretics may cause whole-body electrolyte imbalances, thus resulting in various neurological adverse effects and/or in altered pharmacokinetics of psychoactive drugs. A neuropsychiatric effect of thiazides is not known. Moreover, the lipophilic metolazone might alter electrolyte levels in the brain more efficiently than other non-lipophilic thiazides. The role of CAs has been increasingly studied with respect to synaptic function, spatial learning, memory,^129^ and altering the function of GABA receptor channels. Some lipophilic thiazides (indapamide, metolazone) may contrast seizures through their inhibitory action on CAs, similarly to some antiepileptic drugs that inhibit CAs (e.g. acetazolamide, methazolamide, zonisamide, and topiramate).^130^ CAs functionality is required for proper cognitive and memory processes, whereas insufficient CA activity may be connected with risk of dementia.^131^ CA1 was found to be either down-regulated or up-regulated in depression.^132,133^ Moreover, the antidepressant drugs fluoxetine, sertraline, and citalopram were shown to activate CA1 and CA2,^129^ whereas the CA1 inhibitor acetazolamide was suggested to be useful in treating the depressive phase of bipolar disorders.^134^ Antipsychotic drugs also inhibit CA1 and CA2,^67^ with an unknown functional role.

Among potassium sparing diuretics, amiloride and triamterene are scantly lipophilic. Therefore, at therapeutic dosages, these drugs would unlikely reach significant concentrations in the brain. There is ongoing research with respect to intranasal administrations of amiloride, which should reach the brain more efficiently, but its effects are yet to be defined.^135^

Mineralocorticoid receptors are not currently associated with any neuropsychiatric effect. However, several observations suggest that they are involved in neurogenesis aspects that establish anxious responses to stress.^136^ Furthermore, a decreased response to mineralocorticoid agonists has been found in depression, indicating down-regulation of the mineralocorticoid receptors.^137^ Mineralocorticoid receptors may also take part in addictive behaviours with unclear roles.^138^ Moreover, mineralocorticoid receptors have been shown to inhibit non-transcriptionally the NRG1-ERBB4 pathway, which is involved in the cognitive symptoms of schizophrenia:^139^ indeed, spironolactone is being tested as add-on treatment in schizophrenic patients with cognitive impairments.^140^

The possible role of NKCC1 dysregulation in the aetiopathogenesis of several neuropsychiatric disorders has been investigated. NKCC1 may be a promising target for the treatment of specific epilepsies,^141,142^ neurodevelopmental disorders including autism spectrum disorders and tuberous sclerosis, and neuropsychiatric disorders including schizophrenia, anxiety, pain, and brain degeneration or injury.^143,144^ The therapeutic relevance of treatments based on NKCC1 channels is yet to be proven, also because the correlation between bioavailability in the brain and neuropsychiatric effects is unclear. Furosemide is the most lipophilic NKCC1 blocker and also inhibits the CA; however, it found scarce application. The hydrophilic bumetanide, which reaches the brain to a very limited extent (<1% brain/plasma concentration),^145^ has been extensively tested for autism spectrum disorders, infantile seizures, and other disorders.^10^ Bumetanide may be efficacious for a subset of autistic patients, while it fails to improve symptoms in the average population; its antiepileptic role is harshly contested.^146^ Part of the scepticism around bumetanide depends on its scant permeability to the brain; therefore, several optimization approaches have been applied. One bumetanide lipophilic prodrug, its dimethylaminoethyl ester (BMU5), showed promising results in increasing the seizure threshold after epileptogenic brain insults.^147,148^ However, BUM5 decreased survival in a mouse model of stroke, probably due to toxic effects following accumulation in the brain upon several days of administration.^149^ More recently, the potential anticonvulsant efficacy of the lipophilic bumetanide derivative BUM97 was assessed in *in vivo* models.^150^ The treatment suppressed hippocampal paroxysmal discharges and spike trains in a dose-dependent manner; moreover, it exerted a synergistic anticonvulsant effect with phenobarbital.

### 4.3 Clinical evidence for neuropsychiatric effects of diuretics

Most of the existing data on the role of diuretics in psychiatry are related to the use of bumetanide for treating core symptoms of ASD.^86–91^ Following a pilot study,^151^ 2 placebo-controlled randomized trials tested bumetanide in paediatric patients with ASD.^152,153^ In both trials, bumetanide reduced measures of core autism symptoms significantly. Additional anecdotal evidence came from studies on emotion recognition^154,155^ and reports of single cases of success, for instance in a boy with Fragile X.^92^ Phase 2 trials suggested further efficacy of bumetanide on repetitive behaviours, but no effects on other outcomes of social responsiveness. Phase 3 international trials failed to show significant effect of bumetanide over placebo on any outcome in the latest interim analyses, thus leading to a disappointing stop in drug development.^156^ Despite this positive evidence on the role of bumetanide in treating children with ASD, it must be considered that autism is not a homogeneous psychiatric condition.^157,158^ Some sporadic positive evidence on the role of bumetanide and diazoxide in treating schizophrenia has been reported;^93,94^ however, further research is needed to investigate this hypothesis.

### 4.4 Diuretics—summary

CA-inhibiting lipophilic thiazides may have neuropsychiatric effects on mood and cognition, yet to be thoroughly investigated in preclinical models. Agonism at the NKCC1 receptor is the only mechanism currently targeted in clinical applications for the treatment of autism spectrum disorders and epilepsy of specific aetiology. Thus, the use of bumetanide is limited to subpopulations of patients.^159^ It remains to be clarified how the hydrophilic bumetanide can be more efficacious than the lipophilic furosemide, how it could be better targeted, and whether conditions other than a subset of autism spectrum disorders may be treated with NKCC1 inhibitors. Furthermore, as for future applications, CA inhibitors are being studied as antiepileptic agents, and spironolactone is being studied for addiction and/or schizophrenia. Except for pharmacokinetic effects based on volaemic changes, no interactions with neuropsychiatric drugs are known or expected from diuretics.

## 5. Calcium channel blockers

### 5.1 L-type calcium channels in the brain

CCBs inhibit the inward current of Ca^2+^ ions by binding to the L-type ‘long-acting’ voltage-gated Ca^2+^ channels (LTCCs). These react to membrane potential depolarization by opening, thereby allowing Ca^2+^ to pass into the cytosol^160^ (*Figure 2*). Cytosolic Ca^2+^ can modulate signalling pathways either directly, by binding Ca^2+^-dependent kinases and phosphatases, or indirectly, via the Ca^2+^ binding protein calmodulin.^161^ As LTCCs activity in the brain is connected with neuropsychiatric functions, CCBs might have therapeutic potential.

The LTCCs consist of the α1 subunit, that forms the pore, and of the additional α2δ, β, and γ subunits.^162^ Four different genes (CACNA1S, CACNA1C, CACNA1D, CACNA1F) encode for the α1 subunits of the channel isoforms (Cav1.1-Cav1.4), differing in their biophysical properties, pharmacological specificities, and locations.^162^ The auxiliary subunits play a role in channel membrane localization, activity, and dynamic regulation.^163,164^ All isoforms of LTCCs are sensitive to the main classes of CCBs (dihydropyridines [DHPs], phenylalkylamines, and benzothiazepines), but differ in terms of tissue distribution and gating activity.^165^ The brain isoforms of LTCCs are Cav1.2 and Cav1.3. These are expressed especially in the hippocampus, amygdala, and substantia nigra, though at a different extent (approximately 90% Cav1.2 and 10% Cav1.3).^166,167^ Initial pharmacological assessments, complemented by studies in genetic animal models, have dissected the role of the Cav1.2 and Cav1.3 isoforms in synaptic plasticity and learning and memory processes in CA1 pyramidal neurones in the hippocampus.^168^ These studies showed the specific involvement of Cav1.2 in the consolidation or reconsolidation of spatial memory.^169,170^ Cav1.3 is implicated in the neuronal plasticity and in the age-dependent cognitive decline by allowing the Ca^2+^ influx underlying the slow post-burst after-hyperpolarization (AHP).^171^ Moreover, Cav1.3 is involved in processes regulating the formation of fear memories within the lateral amygdala.^172,173^ Cav1.2 and Cav1.3 channels contribute to modulate the dopaminergic mesoaccumbens pathway, which regulates natural reward sensitivity and behavioural processes underlying drug addiction.^174^ Furthermore, they both are expressed by microglia and appear to play a role in neuroinflammation by triggering secretion of inflammatory signals.^175^

### 5.2 Preclinical evidence for neuropsychiatric effects of CCBs and pharmacodynamic interactions with neuropsychiatric drugs

Despite the structural differences among the three classes, all the CCBs reversibly bind the α1 subunit of the LTCCs and interfere with the voltage-dependent activation of the channel.^160^ Since Cav1.2 and Cav1.3 channels have very similar pharmacological properties, CCBs discriminate between these channels only weakly.^160,166^ However, the analysis of the binding of different dihydropyridines to both channels suggested some selectivity for Cav1.2 of nifedipine and nitrendipine.^160,176^

The data obtained by the genetic modification of brain LTCCs suggest behavioural effects and anxiolytic-like features for CCBs, which are possibly related to the modulation of neurotransmitters release.^177,178^ Preclinical *in vitro* and *in vivo* data showed that CCBs modulate the activity of Cav1.2 and Cav1.3 channels in the brain. However, evidence in hypertensive patients suggests that therapeutic doses of these drugs do not affect brain function.^167^ Nevertheless, these data help understand the mechanism of action of the channels and the therapeutic potential of their inhibitions. In a study carried out in mice that had been treated with CCBs acutely, verapamil and diltiazem facilitated depression, presumably through an off-target decrease of the release in norepinephrine and serotonin at higher dosages. Conversely, nifedipine showed an antidepressant-like behaviour,^178^ in line with a previous paper reporting an antidepressant profile for nifedipine and other DPHs, such as nicardipine, nitrendipine, isradipine, felodipine, and nimodipine. The involvement of the LTCCs in this event was further confirmed by the finding that an activator of the channels, Bay K8644, reduced the effect of nifedipine.^179^*In vivo*, nimodipine reduced fear conditioning, an animal model of fear and anxiety, by significantly depressing neurotransmitters release and excitatory post-synaptic currents (EPSC) in the amygdala.^180^ Recently, it has been reported that diltiazem produces anxiolytic-like effects in mice via the up-regulation of brain neurosteroids, such as tetrahydrodeoxycorticosterone and allopregnanolone.^181^ Blocking LTCCs with isradipine in organotypic slices and mouse models of neurotoxin-induced Parkinson’s disease showed neuroprotection of dopaminergic neurones, probably by inhibiting mitochondrial oxidative stress increase.^182,183^

Noteworthy, CCBs may interact pharmacodynamically with diverse psychoactive drugs. Up-regulation of LTCCs has been reported in rat brain after repeated administration of psychostimulant drugs such as methamphetamine, cocaine, and opioids (e.g. morphine).^184^ However, the concurrent administration of nimodipine prevented the up-regulation of the channels caused by opioid administration.^185^ In general, in preclinical studies, CCBs prevented or reduced important components of dependence and the development of tolerance, presumably by acting at the level of mesolimbic dopamine system on neuronal transmission and synaptic plasticity.^186^ Moreover, it has been recently reported that acute administration of the SSRI fluoxetine reduces the morphine-induced up-regulation of Cav1.2 and Cav1.3 in cortex and mesolimbic; moreover, when administered along with either nimodipine or diltiazem, fluoxetine increases morphine-induced anti-nociception.^187^

### 5.3 Clinical evidence for neuropsychiatric effects of CCBs and pharmacogenetic considerations

CCBs have been studied clinically in psychiatric conditions such as mood disorders and substance abuse/dependence, yielding conflicting results (see Supplementary material online, *Table S1*). Isradipine was effective in improving schizophrenia symptoms in two recent RCTs;^97,98^ however, no clear evidence of a role of CCBs in neurocognition is currently available.^99,100^

Interestingly, genome-wide association studies (GWAS) support a role for the gene codifying for the main target of antihypertensive CCBs, CACNA1C, in bipolar disorders, major depressive disorders, schizophrenia, and ASD.^188–193^ Moreover, variants in CACNA1C have been correlated with sleep latency and sleep quality by GWAS.^194,195^ In a Mendelian randomization study, genetically predicted insomnia was associated with a higher hypertension risk.^196^ Several SNPs in CACNA1C have been linked to psychiatric disorders, with most of them being located in a large intron between exons 3 and 4 (intron 3).^197^ Many studies have been performed on the SNP rs1006737 in CACNA1C. There is a significant association of this polymorphism with bipolar disorders, major depressive disorders, schizophrenia, and ADHD. At molecular level, rs100637 is correlated with changes in CACNA1C expression and an increased L-type current in induced human neurones derived from individuals carrying rs1006737.^198^ Furthermore, the minor allele for rs1006737 (A) is correlated with increased methylation of CpG islands located within intron 3.^199^ Imaging studies have shown associations of rs1006737 with changes in structure and activity of brain regions that are related to emotion processing, memory formation, and cognition.^200,201^ The effect of the rs1006737 genotype and its interaction with LTCC antagonism could be clarified by the Oxford study of Calcium channel Antagonism, Cognition, Mood instability and Sleep (OxCaMS), an exploratory experimental medicine study of the LTCC antagonist nicardipine given to participants with high mood instability.^202^

To date, observational studies have reported contradictory associations between CCBs intake and mood disorders (see Supplementary material online, *Table S1*). In a longitudinal study based on a large hospital database of patients hospitalized for mood disorders, the use of CCBs and β blockers was associated with an increased risk of admission for mood disorders.^101^ Conversely, in a cohort study of patients with serious mental illness, reduced rates of psychiatric hospitalization and self-harm were observed in patients with bipolar disorder and schizophrenia who were treated with CBBs.^203^ Two recent systematic reviews of studies investigating the role of CCBs in bipolar disorder showed no evidence of superiority of verapamil over placebo in treating acute mania.^95,96^ Based on genetic associations between voltage-gated calcium channels and major depression, CCBs have been associated with decreased risk of developing depression in a few uncontrolled clinical trials.^197,204^

### 5.4 CCBs—summary

CCBs interact with the brain isoforms of LTCCs Cav1.2 and Cav1.3, thereby modulating their channel activity. The preclinical evidence suggests that CCBs might have therapeutic value in the treatment of neuropsychiatric disorders, neurodegenerative diseases, and drug dependence. However, human clinical studies are not conclusive about an association between CCBs intake and mood disorders therapy. This might be due to a low CFS drug concentration at the dosage used for cardiovascular disease treatment.

## 6. Renin-angiotensin system modulators

### 6.1 Renin-angiotensin system in the brain

Two renin-angiotensin systems (RAS) exist in the brain, the systemic and the local RAS.^205,206^ However, since the BBB restricts the access of the systemic RAS components to most brain regions, the brain RAS is probably essential in the pathophysiology of several neurodegenerative diseases.^207–209^ The brain RAS plays also an important role in regulating autonomic functions and maintaining cardiovascular homeostasis.^210^ The enzyme renin initiates the RAS pathway by producing angiotensin I (Ang I) through the cleavage of angiotensinogen. Within the brain, renin has been found within neurones and astrocytes,^211^ while angiotensinogen is mainly produced and secreted by astrocytes.^211–213^ Four neuroactive peptides derive from Ang I: Ang II, Ang IV, Ang (1–7), and alamandine. The angiotensin converting enzyme (ACE) cleaves Ang I to generate Ang II, which is the principal effector peptide of RAS and binds two different receptors, the Ang II type I receptor (AT1R) and the type II receptor (AT2R). Ang I and Ang II may generate Ang III, an intermediate peptide that engages AT1R and AT2R and can be further processed to Ang IV. The latter binds Ang II type IV receptor (AT4R) and at high concentration also AT1R. In addition, Ang I or II can be cleaved by the isoform ACE2 to produce Ang (1–7), which binds Mas receptor (MasRs).^214^ Alamandine, an analogue of Ang (1–7), is the most recently discovered peptide of the RAS system; it functions through the interaction with the Mas-related-G protein coupled receptor (MrgD).

AT1Rs, AT2Rs, and MasRs are G-protein coupled receptors (GPCRs) located on neurones, astrocytes and microglia of the cortex, hippocampus, and basal ganglia;^215,216^ MrgDs and AT4Rs have been found only in neurones.^217–219^ The up-regulation of ACE expression and the increased activation of AT1R signalling exacerbate inflammation, cell death, and cognitive impairment.^215,220,221^ On the contrary, AT2Rs, AT4R, MasRs, MrgDs, and ACE2 have antioxidant and anti-inflammatory activity and stimulate cell survival and cognition.^215,222,223^ Neurones, microglia, and astrocytes express AT1Rs and AT2Rs, both at the plasma membrane level and intracellularly, especially in the mitochondria and nuclei.^213,223,224^

Neurones can produce an intracellular form of renin and secrete the precursor form of renin, prorenin; both can bind to prorenin receptors (PRRs), which are also expressed in neurones and lead to angiotensinogen cleavage.^213^

The binding of Ang II to the neuronal plasma membrane AT1R leads to the recruitment of heterotrimeric G proteins (Gq/G11 and/or Gi/Go) and/or adaptor proteins such as JAK2, GRK2, and β-arrestin. This triggers different intracellular pathways such as the production of reactive oxygen species (ROS) via the activation of the NADPH oxidase (NOX).^225,226^ The increase of oxidative stress is also a consequence of the activation of NOX4 via the mitochondrial AT1R; this pathway exacerbates cell death in the cortex, hippocampus, and basal ganglia, which are essential for cognitive functions.^224,227^

Nuclear AT1R, however, induces AT2R expression that functions in an opposite way to AT1R/ACE signalling. Plasma membrane AT2R regulates neuronal excitability and differentiation, and promotes neurite outgrowth and cell survival through the activation of multiple pathways.^228–232^ In mitochondria, AT2R modulates mitochondrial respiration to decrease ROS production.^228^ AT1R and AT2R are almost undetectable in microglia of healthy brain, while they are up-regulated in activated cells in pathological conditions related to inflammation.^220^ Up-regulation of AT1R exacerbates neuronal damage through the activation of NOX signalling and ROS production.^233^ Conversely, the increased expression of AT2R appears to be a compensatory mechanism involved in the switch of microglial cells toward an anti-inflammatory phenotype that blunts ROS production via NOX inhibition.^234^

AT1R activation in astrocytes has both negative and positive outcomes. In neurones and microglia, it leads to the increase of ROS generation via the NOX pathway, to oxidative stress, and to cell death. However, AT1R activation decreases the permeability and maintenance of the BBB through the mobilization of occludin, a protein of the tight junctions, in the lipid rafts of the brain endothelial cell.^227,235^

Regarding the role of brain RAS in hypertension, accumulating evidence indicates that RAS constituents and mediators of inflammation act on the brain within a neural network to increase the sympathetic nervous system activity and, consequently, blood pressure.^236,237^ Furthermore, the increase of Ang II in the brain appears to be one of the compensatory responses of neuro-humoral activation during the development of chronic heart failure.^238^

The brain RAS modulates also sensory information processing, learning and memory, and emotional responses.^239^ Ang II induces dopamine and serotonin release when infused in the rat brain via presynaptic AT1R stimulation.^240,241^ RAS may also promote cell survival by modulating the brain levels of the brain-derived neurotrophic factor (BDNF) and its receptor tropomyosin-related kinase B receptor (TRKB) through the activation of AT2R.^242,243^ The hyper-activation of the brain RAS has been involved in the pathogenesis of several neurodegenerative disorders, such as Alzheimer’s disease, Parkinson’s disease, multiple sclerosis, depression, schizophrenia, and bipolar disorder.^244^ In particular, the pathogenesis of depression and bipolar disorder is linked to hyper-activation of the hypothalamic–pituitary–adrenocortical (HPA) axis:^245,246^ under stress conditions, the increase of Ang II and the activation of AT1R induce gene expression and release of the corticotropin-releasing hormone by the neurosecretory cells in the paraventricular nucleus of the hypothalamus (PVN), thereby triggering the entire cascade of the HPA axis.^247^ A role for RAS in schizophrenia has been hypothesized^248,249^ based on different lines of evidence, such as: *i.* changes in the levels of ACE in the brain of patients affected by schizophrenia;^250^*ii.* the involvement of brain RAS in glutamate-induced oxidative stress and in dopaminergic vulnerability;^227,251,252^*iii.* the positive effects of AT2R activation in neurite outgrowth and elongation, neuronal excitability, and synapse plasticity;^228–232^*iv*. the capability of ACE to cleave peptides such as neurotensin and substance P, which are dysregulated in schizophrenia patients, the former being associated with regulation of dopamine transmission and the latter with neuroinflammation.^253–255^

### 6.2 Preclinical evidence for neuropsychiatric effects of modulation of renin-angiotensin system in the brain

Drugs targeting the RAS at various levels, such as ACEIs, ARBs, and renin inhibitors, are used to treat cardiovascular diseases.^256,257^ Evidence supporting the antidepressant effects of these drugs is increasing. In a recent study in mice, the ACEI captopril and lisinopril showed a rapid and long-lasting beneficial effect on stress-induced depressive-like behaviours.^258^ However, this event was mediated not by a direct action on the RAS, but by potentiating a pathway involving bradykinin, a secondary substrate of ACE, and the mammalian target of rapamycin complex 1 (mTORC1).^258^ Moreover, captopril reduced anxiety behaviour, as did candesartan and losartan, while enalapril did not show any neuropsychiatric effect.^259–261^ Similarly, valsartan, irbesartan, and telmisartan exerted an antidepressant effect, indicating that AT1R stimulation is involved in controlling the cognitive and behavioural responses to stress and anxiety.^262–264^

The beneficial effect of pharmacological modulation of RAS in schizophrenia mainly depends on the neuroprotective properties of ARBs. Regarding treatment of schizophrenia-related cognitive impairment with irbesartan, losartan, and telmisartan, a novel mechanism of action has been hypothesized, which involves the negative regulation of the kynurenine aminotransferase II and the reduction of kynurenic acid.^265^ Indeed, high levels of kynurenic acid in the brain are associated with memory impairment and psychotic symptoms.^266^ Furthermore, the high lipophilic telmisartan is particularly interesting among ARBs as a central modulator in schizophrenia, as it showed the strongest anti-inflammatory activity and the highest affinity for PPAR-γ, which is increased in patients with schizophrenia.^267^ In a mouse model of schizophrenia at peri-pubertal age, candesartan was administered at a dosage below the starting dose used to treat hypertension in children and adolescents. It prevented behavioural alterations with a mechanism that appeared to be independent of AT1R activation and presumably consistent with a direct antioxidant effect of the molecule.^268,269^

### 6.3 Clinical evidence for neuropsychiatric effects of renin-angiotensin-system acting agents and pharmacogenetic considerations

Although it has been reported that ACEIs have positive effects on mood when compared with patients taking other antihypertensive drugs,^99,101–103^ there is scant evidence on the use of ACEIs in the field of neuropsychiatry (see Supplementary material online, *Table S1*). Associations between ACE polymorphisms and depression have been described,^270,271^ and altered methylation of the regulatory region of the ACE gene has been reported in depression.^272^ A great body of evidence suggested that ACE insertion (I)/deletion (D) polymorphism (rs4646994) (ACE I/D) may be associated with depression;^273^ moreover, this polymorphism presumably plays a role in the modulation of serotonergic and dopaminergic turnover in the human CNS.^274^ In addition, the SNP rs4291, which is located in the promoter region of the ACE gene and influences ACE activity and HPA-axis hyperactivity, was associated with unipolar major depression.^273^ In a case-control study including 961 individuals^102^ and in a subsequent study using Danish nationwide population-based registers,^103^ clinical evidence confirmed that ACEIs were associated with a decreased rate of onset of depression.

Genetic studies^275,276^ have identified an association between the ACE I/D polymorphism (rs4646994) and schizophrenia; however, the findings are contradictory. The first case-control study showed that the II genotype of the I/D polymorphism had a protective effect for schizophrenia among females, whereas there was no significant association between I/D polymorphism and susceptibility to schizophrenia among male subjects.^275^ Conversely, a meta-analysis found no association between the ACE I/D polymorphism and schizophrenia.^276^ Similarly, inconsistent findings have been reported for ACE activity and protein levels with regard to schizophrenia.^248,277^ In a study, ACE activity was decreased in the plasma of patients with schizophrenia compared with healthy controls; however, it was increased in another study. The Schizophrenia Working Group of the Psychiatric Genetics Consortium^278^ published a preprint of the largest GWAS of schizophrenia, which included 69 369 cases and 236 642 controls. Using fine-mapping and functional genomic data, the authors prioritized 19 genes based on protein-coding or UTR variation, including the well-known candidate CACNA1C. Furthermore, through a summary-based Mendelian randomization analysis, they showed an association of decreased ACE expression in blood with increased risk of schizophrenia. This analysis parallel with the findings of another Mendelian randomization study that suggested an association of lower levels of ACE (messenger RNA and protein) with decreased systolic blood pressure (SBP) on one side, and with an increased risk of schizophrenia on the other side.^279^ There was no evidence for an association between genetically estimated SBP and schizophrenia risk, suggesting that any association of ACE with schizophrenia is presumably independent of its association with blood pressure.

Beside major depressive disorder, SNPs within the ACE gene were also significantly associated with acute stress response and higher mortality following a major trauma.^273,280,281^ In particular, the rs4311 SNP within the ACE gene, that has been associated with the panic attack syndrome, might be a functional polymorphism that increases risk for dysregulated fear responses.^282^ In a cohort study recruiting trauma-exposed individual, this polymorphism was associated with PTSD symptoms and diagnosis, as the T-carriers at the rs4311 SNP had significantly greater likelihood of a PTSD diagnosis.^283^

A large meta-analysis suggested that the insertion (I)-deletion (D) polymorphism of the ACE gene could be a marker of Alzheimer’s disease (AD).^284^ In particular, the D allele was associated with raised plasma levels of the enzyme,^285^ and the genotype DD was protective for AD risk;^284^ conversely, the presence of the I allele was associated with an increased risk of AD.^286,287^ This association could be due to linkage disequilibrium with the true risk factor. A very recent study supposed that the effects of the ACE insertion/deletion polymorphism might differ according to the apolipoprotein E ε4 (APOE*4) carrier status, the only fully established susceptibility allele for AD.^288^

With regard to ARBs, data in humans are currently very limited. Clinical reports and observational studies recently reported encouraging findings in psychotic patients;^105,106^ protective effects of ARBs on cognition,^107,108^ depression, and anxiety have also been reported (see Supplementary material online, *Table S1*). According to recent findings from a meta-analysis of 11 RCTs comparing ACEIs or ARBs versus either placebo or non-ACEI or non-ARBs, the use of ACEI or ARBs was significantly associated with improved mental health domains in adults (overall quality of life, positive wellbeing, mental and anxiety domains of quality of life).^104^

Noteworthy, in a recent 12-week randomized, double-blind, placebo-controlled study, the adjunctive treatment with telmisartan improved schizophrenia symptoms in 22 patients receiving either clozapine or olanzapine.^106^ However, no adjustments were made for multiple comparisons; moreover, due to the limited duration of the trial and the small simple size, no firm conclusion can be drawn. In a nested case-control study investigating all antihypertensive drug classes, suicide risk was associated only with ARB use (OR: 3.52; 95% CI: 1.33–9.30).^109^

### 6.4 Renin-angiotensin system modulators—summary

Increasing preclinical evidence supports the antidepressant and antipsychotic effects of drugs targeting the RAS. Particularly interesting are ARBs, with their neuroprotective properties exerted through the stimulation of AT1R and its downstream pathways. The psychotropic potential of these medications is supported by studies demonstrating a genetic association between ACE polymorphisms and depression/schizophrenia. The available clinical evidence further points to a psychotropic role of RAS modulators. However, the intrinsic limitations of the studies that have addressed the topic (e.g. small cohort size, observational nature, small period of observation) support the need for further trials with larger sample sizes and longer treatment durations. Such studies should aim to characterize the beneficial effects of RAS-acting agents on psychopathology, cognition, and safety in patients with schizophrenia and mood disorders, along with the potential mechanism mediating these effects and pharmacogenetic aspects.^249^

## 7. Other antihypertensive drugs

### 7.1 Vasodilators

The blood vessel dilating drugs hydralazine and minoxidil have important off-target activities in the brain that do not involve vasodilation; however, since preclinical and clinical evidence is scant, we only provide a summary of the available data.

The hydrophilic drug hydralazine is thought to act as an antioxidant by preventing the degradation of NO and thereby increasing its activity. Hydralazine can also inhibit inositol-triphosphate channels, which control calcium release from intracellular storages. Calcium concentrations in the neurone participate in controlling survival, structural, and functional aspects, which are connected also with memory and neurodegeneration.^289–291^ A therapeutic role for hydralazine is currently only hypothetical; furthermore, its high hydrophilicity and intense antihypertensive effect may prevent the possibility to reach useful concentrations in the brain.

Minoxidil is a lipophilic opener of ATP-sensitive potassium channels (K_ATP_), which can trigger the relaxation of blood vessels smooth muscles and play a crucial role in peripheral and hypothalamic glycaemic regulation. Beside these roles, K_ATP_ channels can couple the electrical activity of neurones with a check of energy availability, possibly playing a role in brain injury following ischaemia or hypoxia. Indeed, K_ATP_ channels dysfunction has been putatively connected with neurodegeneration, in particular of dopaminergic neurones.^292^ Minoxidil hypothetically plays a role in neuroprotection following toxic damage or ischaemia, as shown in cardiomyocytes,^293,294^ but no studies have been conducted on patients yet.

### 7.2 Reserpine

Reserpine is a lipophilic monoamine depleting agent that irreversibly inhibits the vesicular monoamine transporter (VMAT), which reuptakes norepinephrine, serotonin, and dopamine from the cytosol into presynaptic vesicles (*Figure 2*). The consequent increase in the cytosolic concentration of monoamines leads to their degradation by monoamine oxidase and to depletion. Subsequently, monoamines have to be synthesized anew. This action of reserpine is depressant. In addition, reserpine may also induce neurone loss, due to an increased energy expenditure for neurotransmitter synthesis. Indeed, there are pathological models of pain, depression, and neurodegeneration induced by reserpine treatment.^295,296^ There are scant observations of neuropsychiatric effects of reserpine in clinical practice or clinical trials.

## 8. Conclusion and perspectives

Preclinical studies have explored the molecular mechanisms through which antihypertensive drugs lead to neuropsychiatric effects; however, clinical data are currently insufficient to recommend the use of most antihypertensive drugs based on possible beneficial/harmful effects in selected psychiatric comorbid patients. Notable exceptions are lipophilic noradrenergic antihypertensive drugs, which cause symptoms attributable to depression, and bumetanide, which may obtain a therapeutic placement for autism or epilepsy in specific patients. When treating psychiatric comorbid patients, knowledge of the neuropsychiatric mechanisms of action and effects of antihypertensive drugs can help discriminate between psychiatric comorbidity and the adverse effects (or unexpected positive effects) that may occur.

Overall, the effects of adrenergic modulators depend on the engaged receptors. α2 agonists, recently approved for the treatment of ADHD, promote cognitive functions and potentiate antipsychotic effects, while increasing the adverse effects of antidepressants. They relieve anxiety and PTSD symptoms, and show beneficial effects in the management of OCD and schizophrenia. α1 antagonists reduce fear-related memories and enhance cognition, even under stressful conditions. Prazosin, thanks to its anxiolytic effect, is used to treat PTSD by reducing the overall burden of disease symptoms. Similarly, β antagonists show a potential benefit in schizophrenia, anxiety, ASD, and behaviour disorders, but induce symptoms of depression, including fatigue and lethargy, and sleep disruption, thereby reducing the efficacy of some antidepressant medications. Limited data are available on the effects of diuretic drugs in the field of neuropsychiatry. The inhibition/antagonism of NKCC1 receptor is the only mechanism currently targeted in clinical applications for the treatment of ASD and epilepsy of specific aetiology, limiting their use to specific subpopulations of patients. For future applications, clinical studies have been exploring the potential use of CA inhibitors as antiepileptic agents and of spironolactone for treatment of addiction and/or schizophrenia.

CCBs are associated with a relative low rate of psychiatric complications, but also with a poor positive effect on brain function. Under debate is their potential role in improving or worsening cognitive functions and mood disorders. CCBs seem to reduce psychiatric hospitalization in patients with schizophrenia, whereas no evidence supports their role to improve acute mania and bipolar disorders, or an antidepressant effect. Evidence of neuropsychiatric effects of drugs targeting RAS is currently almost absent. However, ACEIs and ARBs rescue anxiety- and depression-like phenotypes in animal models, and seem to improve mental health and schizophrenia symptoms in human.

Open questions regard on the one hand how to avoid undesirable effects or interactions with neuropsychiatric drugs and, on the other hand, how to exploit these additional mechanisms for neuropsychiatric treatment. Adverse effects can be avoided by switching drugs, for instance to a less lipophilic equivalent, or by choosing the proper combination between antihypertensive and neuropsychiatric drugs to avoid undesired interactions. Therapeutic development of antihypertensive drugs can be pursued firstly by solving the bioavailability issue, since most have limited penetration in the CNS. There are three different approaches to improve the pharmacological profile of these drugs. One is to use lipophilic prodrugs that more easily penetrate the brain barriers and are subsequently metabolized, once within the CNS, to release the active moieties. The second approach is to design lipophilic derivatives of antihypertensive drugs that cross the brain barriers more easily than the parent compound. The third is to modify those antihypertensive drugs that have different targets in the CNS, reducing their antihypertensive potential that may be excessive or untoward when considering neuropsychiatric applications.

Future studies should include outcomes of neuropsychiatric interest, because previous studies have focussed on cardiovascular outcomes and only reported anecdotal evidence on neuropsychiatric aspects. The Mendelian randomization approach may provide an additional piece of evidence about the effects of drugs on certain neurological diseases, especially since RCTs are sometimes too expensive or even impossible to perform. Some studies have already applied this approach to investigate the effects of antihypertensive drugs in the context of neuropsychiatric diseases.^297–299^

## Supplementary material

Supplementary material is available at *Cardiovascular Research* online.

## Supplementary Material

cvac110_Supplementary_Data

## Data Availability

Data derived from sources in the public domain. Reference details are provided in full (both in the Main text and in the Supplementary Material online).

## References

[1] Amadio P , Zarà M, Sandrini L, Ieraci A, Barbieri SS. Depression and cardiovascular disease: the viewpoint of platelets. Int J Mol Sci 2020;21:1–34. 10.3390/ijms21207560 PMC7589256 33066277

[2] Sandrini L , Ieraci A, Amadio P, Zarà M, Barbieri SS. Impact of acute and chronic stress on thrombosis in healthy individuals and cardiovascular disease patients. Int J Mol Sci 2020;21:7818. 33105629 10.3390/ijms21217818 PMC7659944

[3] Vaccarino V , Badimon L, Bremner JD, Cenko E, Cubedo J, Dorobantu M, Duncker DJ, Koller A, Manfrini O, Milicic D, Padro T, Pries AR, Quyyumi AA, Tousoulis D, Trifunovic D, Vasiljevic Z, de Wit C, Bugiardini R; ESC Scientific Document Group Reviewers. Depression and coronary heart disease: 2018 position paper of the ESC working group on coronary pathophysiology and microcirculation. Eur Heart J 2020;41:1687–1696. 30698764 10.1093/eurheartj/ehy913 PMC10941327

[4] de Hert M , Detraux J, Vancampfort D. The intriguing relationship between coronary heart disease and mental disorders. Dialog Clin Neurosci 2018;20:31–40. 10.31887/DCNS.2018.20.1/mdehert PMC6016051 29946209

[5] Korosi B , Laszlo A, Tabak A, Batta D, Lenart L, Fekete A, Eorsi D, Cseprekal O, Tisler A, Nemcsik-Bencze Z, Gonda X, Rihmer Z, Nemcsik J. The impact of currently recommended antihypertensive therapy on depression and other psychometric parameters: preliminary communication. Neuropsychopharmacol Hung 2017;19:11–22. 28467955

[6] Zou S , Hu J. Mental illness and hypertension. Secondary Hypertension: Screening, Diagnosis and Treatment. 2019:389-402.

[7] Qin J , He Z, Wu L, Wang W, Lin Q, Lin Y, Zheng L. Prevalence of mild cognitive impairment in patients with hypertension: a systematic review and meta-analysis. Hypertens Res 2021;44:1251–1260. 34285378 10.1038/s41440-021-00704-3

[8] Graham N , Smith DJ. Comorbidity of depression and anxiety disorders in patients with hypertension. J Hypertens 2016;34:397–398. 26818922 10.1097/HJH.0000000000000850

[9] Sumner JA , Kubzansky LD, Roberts AL, Gilsanz P, Chen Q, Winning A, Forman JP, Rimm EB, Koenen KC. Post-traumatic stress disorder symptoms and risk of hypertension over 22 years in a large cohort of younger and middle-aged women. Psychol Med 2016;46:3105–3116. 27534802 10.1017/S0033291716001914 PMC5093068

[10] Kharod SC , Kang SK, Kadam SD. Off-label use of bumetanide for brain disorders: an overview. Front Neurosci 2019;13:310. 31068771 10.3389/fnins.2019.00310 PMC6491514

[11] Corbett A , Pickett J, Burns A, Corcoran J, Dunnett SB, Edison P, Hagan JJ, Holmes C, Jones E, Katona C, Kearns I, Kehoe P, Mudher A, Passmore A, Shepherd N, Walsh F, Ballard C. Drug repositioning for Alzheimer’s disease. Nat Rev Drug Discov 2012;11:833–846. 23123941 10.1038/nrd3869

[12] Solmi M , Tiihonen J, Lähteenvuo M, Tanskanen A, Correll CU, Taipale H. Antipsychotics use is associated with greater adherence to cardiometabolic medications in patients with schizophrenia: results from a nationwide, within-subject design study. Schizophr Bull 2022;48:166–175. 34286338 10.1093/schbul/sbab087 PMC8781351

[13] Carnovale C , Lucenteforte E, Battini V, Mazhar F, Fornili M, Invernizzi E, Mosini G, Gringeri M, Capuano A, Scavone C, Nobile M, Vantaggiato C, Pisano S, Bravaccio C, Radice S, Clementi E, Pozzi M. Association between the glyco-metabolic adverse effects of antipsychotic drugs and their chemical and pharmacological profile: a network meta-analysis and regression. Psychol Med 2021:1–13. 10.1017/S0033291721000180 33622426

[14] Neumaier F , Zlatopolskiy BD, Neumaier B. Drug penetration into the central nervous system: pharmacokinetic concepts and in vitro model systems. Pharmaceutics 2021;13:1542. 34683835 10.3390/pharmaceutics13101542 PMC8538549

[15] Sigaroudi A , Kinzig M, Wahl O, Stelzer C, Schroeter M, Fuhr U, Holzgrabe U, Sörgel F. Quantification of hydrochlorothiazide and ramipril/ramiprilate in blood serum and cerebrospinal fluid: a pharmacokinetic assessment of central nervous system adverse effects. Pharmacology 2018;102:133–137. 29982257 10.1159/000489999

[16] Naito K , Nagao T, Otsuka M, Harigaya S, Nakajima H. Penetration into and elimination from the cerebrospinal fluid of diltiazem, a calcium antagonist, in anesthetized rabbits. Arzneimittelforschung 1986;36:25–28. 3954820

[17] Narang PK , Blumhardt CL, Doran AR, Pickar D. Steady-state cerebrospinal fluid transfer of verapamil and metabolites in patients with schizophrenia. Clin Pharmacol Ther 1988;44:550–557. 3180637 10.1038/clpt.1988.193

[18] Töpfer M , Töllner K, Brandt C, Twele F, Bröer S, Löscher W. Consequences of inhibition of bumetanide metabolism in rodents on brain penetration and effects of bumetanide in chronic models of epilepsy. Eur J Neurosci 2014;39:673–687. 24251546 10.1111/ejn.12424

[19] Arnsten AFT , Wang MJ, Paspalas CD. Neuromodulation of thought: flexibilities and vulnerabilities in prefrontal cortical network synapses. Neuron 2012;76:223–239. 23040817 10.1016/j.neuron.2012.08.038 PMC3488343

[20] Jackman SL , Regehr WG. The mechanisms and functions of synaptic facilitation. Neuron 2017;94:447–464. 28472650 10.1016/j.neuron.2017.02.047 PMC5865607

[21] Luo F , Tang H, Li B-M, Li S-H. Activation of α1-adrenoceptors enhances excitatory synaptic transmission via a pre- and postsynaptic protein kinase C-dependent mechanism in the medial prefrontal cortex of rats. Eur J Neurosci 2014;39:1281–1293. 24494713 10.1111/ejn.12495

[22] Delmas P , Brown DA. Pathways modulating neural KCNQ/M (Kv7) potassium channels. Nat Rev Neurosci 2005;6:850–862. 16261179 10.1038/nrn1785

[23] van der Horst J , Greenwood IA, Jepps TA. Cyclic AMP-dependent regulation of Kv7 voltage-gated potassium channels. Front Physiol 2020;11:727. 32695022 10.3389/fphys.2020.00727 PMC7338754

[24] Birnbaum S , Gobeske KT, Auerbach J, Taylor JR, Arnsten AFT. A role for norepinephrine in stress-induced cognitive deficits: α-1-Adrenoceptor mediation in the prefrontal cortex. Biol Psychiatry 1999;46:1266–1274. 10560032 10.1016/s0006-3223(99)00138-9

[25] Szot P , White SS, Greenup JL, Leverenz JB, Peskind ER, Raskind MA. α1-Adrenoreceptor in human hippocampus: binding and receptor subtype mRNA expression. Brain Res Mol Brain Res 2005;139:367–371. 16039007 10.1016/j.molbrainres.2005.06.013

[26] Arnsten AFT . Stress signalling pathways that impair prefrontal cortex structure and function. Nat Rev Neurosci 2009;10:410–422. 19455173 10.1038/nrn2648 PMC2907136

[27] Arnsten AFT . Through the looking glass: differential noradenergic modulation of prefrontal cortical function. Neural Plasticity 2000;7:133–146. 10709220 10.1155/NP.2000.133 PMC2565372

[28] McCormick DA , Pape HC, Williamson A. Actions of norepinephrine in the cerebral cortex and thalamus: implications for function of the central noradrenergic system. Progr Brain Res 1991;88:293–305. 10.1016/s0079-6123(08)63817-0 1726028

[29] O’Dell TJ , Connor SA, Guglietta R, Nguyen PV. β-Adrenergic receptor signaling and modulation of long-term potentiation in the mammalian hippocampus. Learn Mem 2015;22:461–471. 26286656 10.1101/lm.031088.113 PMC4561407

[30] Ferry B , McGaugh JL. Role of amygdala norepinephrine in mediating stress hormone regulation of memory storage. Acta Pharmacol Sin 2000;21:481–493. 11360681

[31] Ikegaya Y , Nakanishi K, Saito H, Abe K. Amygdala β-noradrenergic influence on hippocampal long-term potentiation in vivo. NeuroReport 1997;8:3143–3146. 9331930 10.1097/00001756-199709290-00027

[32] Osorio-Forero A , Cardis R, Vantomme G, Guillaume-Gentil A, Katsioudi G, Devenoges C, Fernandez LMJ, Lüthi A. Noradrenergic circuit control of non-REM sleep substates. Curr Biol 2021;31:5009–5023.e7. 34648731 10.1016/j.cub.2021.09.041

[33] Yu X , Franks NP, Wisden W. Sleep and sedative states induced by targeting the histamine and noradrenergic systems. Front Neural Circuits 2018;12:4. 29434539 10.3389/fncir.2018.00004 PMC5790777

[34] Mitchell HA , Weinshenker D. Good night and good luck: norepinephrine in sleep pharmacology. Biochem Pharmacol 2010;79:801–809. 19833104 10.1016/j.bcp.2009.10.004 PMC2812689

[35] Stone LS , MacMillan LB, Kitto KF, Limbird LE, Wilcox GL. The α(2a) adrenergic receptor subtype mediates spinal analgesia evoked by α2 agonists and is necessary for spinal adrenergic-opioid synergy. J Neurosci 1997;17:7157–7165. 9278550 10.1523/JNEUROSCI.17-18-07157.1997 PMC6573259

[36] Pozzi M , Mauri M, Bertella S, Gatti E, Nobile M. Comprehensive Pharmacology - 1st Edition. Attention Deficit Hyperactivity Disorder. https://www.elsevier.com/books/comprehensive-pharmacology/kenakin/978-0-12-820472-6.

[37] Ju JY , Kim KM, Lee S. Effect of preoperative administration of systemic alpha-2 agonists on postoperative pain: a systematic review and meta-analysis. Anesth Pain Med 2020;15:157–166. 10.17085/apm.2020.15.2.157 PMC7713826 33329808

[38] Lucas EK , Wu WC, Roman-Ortiz C, Clem RL. Prazosin during fear conditioning facilitates subsequent extinction in male C57Bl/6N mice. Psychopharmacology (Berl) 2019;236:273–279. 30112577 10.1007/s00213-018-5001-x PMC6374171

[39] Stoschitzky K , Sakotnik A, Lercher P, Zweiker R, Maier R, Liebmann P, Lindner W. Influence of beta-blockers on melatonin release. Eur J Clin Pharmacol 1999;55:111–115. 10335905 10.1007/s002280050604

[40] Uys MM , Shahid M, Harvey BH. Therapeutic potential of selectively targeting the α2C -adrenoceptor in cognition, depression, and schizophrenia—new developments and future perspective. Front Psychiatry 2017;8:144. 28855875 10.3389/fpsyt.2017.00144 PMC5558054

[41] Hirota T , Schwartz S, Correll CU. Alpha-2 agonists for attention-deficit/hyperactivity disorder in youth: a systematic review and meta-analysis of monotherapy and add-on trials to stimulant therapy. J Am Acad Child Adolesc Psychiatry 2014;53:153–173. 24472251 10.1016/j.jaac.2013.11.009

[42] Butterfield ME , Saal J, Young B, Young JL. Supplementary guanfacine hydrochloride as a treatment of attention deficit hyperactivity disorder in adults: a double blind, placebo-controlled study. Psychiatry Res 2016;236:136–141. 26730446 10.1016/j.psychres.2015.12.017

[43] Taylor FB , Russo J. Comparing guanfacine and dextroamphetamine for the treatment of adult attention-deficit/hyperactivity disorder. J Clin Psychopharmacol 2001;21:223–228. 11270920 10.1097/00004714-200104000-00015

[44] Iwanami A , Saito K, Fujiwara M, Okutsu D, Ichikawa H. Efficacy and safety of guanfacine extended-release in the treatment of attention-deficit/hyperactivity disorder in adults: results of a randomized, double-blind, placebo-controlled study. J Clin Psychiatry 2020;81:19m12979. 10.4088/JCP.19m12979 32297719

[45] Cortese S , Adamo N, Del Giovane C, Mohr-Jensen C, Hayes AJ, Carucci S, Atkinson LZ, Tessari L, Banaschewski T, Coghill D, Hollis C, Simonoff E, Zuddas A, Barbui C, Purgato M, Steinhausen HC, Shokraneh F, Xia J, Cipriani A. Comparative efficacy and tolerability of medications for attention-deficit hyperactivity disorder in children, adolescents, and adults: a systematic review and network meta-analysis. Lancet Psychiatry 2018;5:727–738. 30097390 10.1016/S2215-0366(18)30269-4 PMC6109107

[46] Barrett JR , Tracy DK, Giaroli G. To sleep or not to sleep: a systematic review of the literature of pharmacological treatments of insomnia in children and adolescents with attention-deficit/hyperactivity disorder. J Child Adolesc Psychopharmacol 2013;23:640–647. 24261659 10.1089/cap.2013.0059 PMC3870602

[47] Hazell PL , Stuart JE. A randomized controlled trial of clonidine added to psychostimulant medication for hyperactive and aggressive children. J Am Acad Child Adolesc Psychiatry 2003;42:886–894. 12874489 10.1097/01.CHI.0000046908.27264.00

[48] Kollins SH , Jain R, Brams M, Segal S, Findling RL, Wigal SB, Khayrallah M. Clonidine extended-release tablets as add-on therapy to psychostimulants in children and adolescents with ADHD. Pediatrics 2011;127:e1406–e1413. 21555501 10.1542/peds.2010-1260 PMC3387872

[49] Ye L , Lippmann S. Reduction of anxiety after treatment with transdermal clonidine. Am J Health Syst Pharm 2018;75:742–744. 29802109 10.2146/ajhp180064

[50] Ye L , Shipley E, Lippmann S. Transdermal clonidine for mitigating posttraumatic stress disorder in an adolescent. Am J Health Syst Pharm 2019;76:487–488. 31361867 10.1093/ajhp/zxz021

[51] Jain R , Segal S, Kollins SH, Khayrallah M. Clonidine extended-release tablets for pediatric patients with attention-deficit/hyperactivity disorder. J Am Acad Child Adolesc Psychiatry 2011;50:171–179. 21241954 10.1016/j.jaac.2010.11.005

[52] Panther SG , Knotts AM, Odom-Maryon T, Daratha K, Woo T, Klein TA. Off-label prescribing trends for ADHD medications in very young children. J Pediatr Pharmacol Ther 2017;22:423–429. 29290742 10.5863/1551-6776-22.6.423 PMC5736254

[53] Connor DF , Grasso DJ, Slivinsky MD, Pearson GS, Banga A. An open-label study of guanfacine extended release for traumatic stress related symptoms in children and adolescents. J Child Adolesc Psychopharmacol 2013;23:244–251. 23683139 10.1089/cap.2012.0119 PMC3657282

[54] Akouchakian S , Tarrahi MJ, Mohebati E. Evaluation of clonidine augmentation therapy for obsessive-compulsive disorder treatment; a randomized clinical trial. Iran J Psychiatry Behav Sci 2021;15:e112131.

[55] Ingrassia A , Turk J. The use of clonidine for severe and intractable sleep problems in children with neurodevelopmental disorders: a case series. Eur Child Adolesc Psychiatry 2005;14:34–40. 15756514 10.1007/s00787-005-0424-4

[56] Aksu MH , Arikan Z. Successful treatment of auditory hallucinations in a schizophrenia patient with the use of clonidine. Dusunen Adam 2019;32:271–274.

[57] Dardennes RM , Al Anbar NN, Rouillon F. Successful augmentation of clozapine-resistant treatment of schizophrenia with clonidine. Prog Neuropsychopharmacol Biol Psychiatry 2010;34:724–725. 20307621 10.1016/j.pnpbp.2010.03.022

[58] Lerner AG , Gelkopf M, Oyffe I, Finkel B, Katz S, Sigal M, Weizman A. LSD-induced hallucinogen persisting perception disorder treatment with clonidine: an open pilot study. Int Clin Psychopharmacol 2000;15:35–37. 10836284 10.1097/00004850-200015010-00005

[59] Elbe D , Perel-Panar C, Wicholas L. Manic reaction in a child induced by guanfacine-extended release. J Child Adolesc Psychopharmacol 2016;26:566–567. 27228067 10.1089/cap.2016.0050

[60] Delaney J , Spevack D, Doddamani S, Ostfeld R. Clonidine-induced delirium. Int J Cardiol 2006;113:276–278. 16545874 10.1016/j.ijcard.2005.09.032

[61] Reist C , Streja E, Tang CC, Shapiro B, Mintz J, Hollifield M. Prazosin for treatment of post-traumatic stress disorder: a systematic review and meta-analysis. CNS Spectr 2021;26:338–344. 32362287 10.1017/S1092852920001121

[62] Khachatryan D , Groll D, Booij L, Sepehry AA, Schütz CG. Prazosin for treating sleep disturbances in adults with posttraumatic stress disorder: a systematic review and meta-analysis of randomized controlled trials. Gen Hosp Psychiatry 2016;39:46–52. 26644317 10.1016/j.genhosppsych.2015.10.007

[63] George KC , Kebejian L, Ruth LJ, Miller CWT, Himelhoch S. Meta-analysis of the efficacy and safety of prazosin versus placebo for the treatment of nightmares and sleep disturbances in adults with posttraumatic stress disorder. J Trauma Dissociation 2016;17:494–510. 26835889 10.1080/15299732.2016.1141150

[64] Singh B , Hughes AJ, Mehta G, Erwin PJ, Parsaik AK. Efficacy of prazosin in posttraumatic stress disorder: a systematic review and meta-analysis. Prim Care Companion CNS Disord 2016;18. 10.4088/PCC.16r01943 27828694

[65] Zhang Y , Ren R, Sanford LD, Yang L, Ni Y, Zhou J, Zhang J, Wing YK, Shi J, Lu L, Tang X. The effects of prazosin on sleep disturbances in post-traumatic stress disorder: a systematic review and meta-analysis. Sleep Med 2020;67:225–231. 31972510 10.1016/j.sleep.2019.06.010 PMC6986268

[66] Petrakis IL , Desai N, Gueorguieva R, Gueorguieva R, Arias A, O'Brien E, Jane JS, Sevarino K, Southwick S, Ralevski E. Prazosin for veterans with posttraumatic stress disorder and comorbid alcohol dependence: a clinical trial. Alcohol Clin Exp Res 2016;40:178–186. 26683790 10.1111/acer.12926

[67] Akinsanya A , Marwaha R, Tampi RR. Prazosin in children and adolescents with posttraumatic stress disorder who have nightmares: a systematic review. J Clin Psychopharmacol 2017;37:84–88. 27930498 10.1097/JCP.0000000000000638

[68] Gopalakrishna G , Popoola O, Campbell A, Nemetalla MA. Two case reports on use of prazosin for drug dreams. J Addict Med 2016;10:131–133. 26900667 10.1097/ADM.0000000000000194

[69] Aggarwal A , Lindegaard V. The use of prazosin in treatment of drug dreams in adolescents with substance use disorder: two case reports. Psychopharmacol Bull 2020;50:29–31. 33012871 10.64719/pb.4371 PMC7511147

[70] Steenen SA , Van Wijk AJ, Van Der Heijden GJMG, Van Westrhenen R, De Lange J, De Jongh A. Propranolol for the treatment of anxiety disorders: systematic review and meta-analysis. J Psychopharmacol 2016;30:128–139. 26487439 10.1177/0269881115612236 PMC4724794

[71] Beversdorf DQ , Saklayen S, Higgins KF, Bodner KE, Kanne SM, Christ SE. Effect of propranolol on word fluency in autism. Cogn Behav Neurol 2011;24:11–17. 21487259 10.1097/WNN.0b013e318204d20e

[72] Beversdorf DQ , Carpenter AL, Miller RF, Cios JS, Hillier A. Effect of propranolol on verbal problem solving in autism spectrum disorder. Neurocase 2008;14:378–383. 18766980 10.1080/13554790802368661

[73] Bodner KE , Beversdorf DQ, Saklayen SS, Christ SE. Noradrenergic moderation of working memory impairments in adults with autism spectrum disorder. J Int Neuropsychol Soc 2012;18:556–564. 22414705 10.1017/S1355617712000070

[74] Zamzow RM , Ferguson BJ, Stichter JP, Porges EC, Ragsdale AS, Lewis ML, Beversdorf DQ. Effects of propranolol on conversational reciprocity in autism spectrum disorder: a pilot, double-blind, single-dose psychopharmacological challenge study. Psychopharmacology (Berl) 2016;233:1171–1178. 26762378 10.1007/s00213-015-4199-0

[75] Hegarty JP , Zamzow RM, Ferguson BJ, Christ SE, Porges EC, Johnson JD, Beversdorf DQ. Beta-adrenergic antagonism alters functional connectivity during associative processing in a preliminary study of individuals with and without autism. Autism 2020;24:795–801. 31416333 10.1177/1362361319868633 PMC7021567

[76] Fisher AA , Davis M, Jeffery I. Acute delirium induced by metoprolol. Cardiovasc Drugs Ther 2002;16:161–165. 12090909 10.1023/a:1015761618314

[77] Chang C-H , Yang Y-HK, Lin S-J, Su J-J, Cheng C-L, Lin L-J. Risk of insomnia attributable to β-blockers in elderly patients with newly diagnosed hypertension. Drug Metab Pharmacokinet 2013;28:53–58. 22813717 10.2133/dmpk.dmpk-12-rg-004

[78] Kogoj A . Suspected propranolol-induced delirium. Can J Psychiatry 2004;49:645. 10.1177/070674370404900920 15503741

[79] Maebara C , Ohtani H, Sugahara H, Mine K, Kubo C, Sawada Y. Nightmares and panic disorder associated with carvedilol overdose. Ann Pharmacother 2002;36:1736–1740. 12398570 10.1345/aph.1A476

[80] Shahrbabaki ME , Estilaee F, Shahrbabaki AE. Can low-dose propranolol induce a manic syndrome? Case report of an unexpected side effect. Acta Neuropsychiatr 2013;25:184–186. 25287473 10.1111/acn.12021

[81] Zhao Y , Xu W, Qiu L, Yang W. Metoprolol-induced psychosis in a young patient. Gen Hosp Psychiatry 2013;35:102.e1–102.e2. 10.1016/j.genhosppsych.2012.03.007 22516214

[82] Butt JH , Dalsgaard S, Torp-Pedersen C, Køber L, Gislason GH, Kruuse C, Fosbøl EL. Beta-blockers for exams identify students at high risk of psychiatric morbidity. J Child Adolesc Psychopharmacol 2017;27:266–273. 27782771 10.1089/cap.2016.0079

[83] Ahmed AIA , van Mierlo P, Jansen P. Sleep disorders, nightmares, depression and anxiety in an elderly patient treated with low-dose metoprolol. Gen Hosp Psychiatry 2010;32:646.e5–646.e7. 10.1016/j.genhosppsych.2010.04.008 21112460

[84] Al-Dury S , Molinaro A, Hedenström P. Propranolol-induced hallucinations mimicking encephalopathy in a patient with liver cirrhosis. Scand J Gastroenterol 2021;56:829–831. 33961526 10.1080/00365521.2021.1919198

[85] Colbourne L , Luciano S, Harrison PJ. Onset and recurrence of psychiatric disorders associated with anti-hypertensive drug classes. Transl Psychiatry 2021;11:319. 34039956 10.1038/s41398-021-01444-1 PMC8155006

[86] Mollajani R , Joghataei MT, Tehrani-Doost M. Review paper: Bumetanide therapeutic effect in children and adolescents with autism spectrum disorder: a review study. Basic Clin Neurosci 2019;10:433–441. 32284832 10.32598/bcn.9.10.380 PMC7149950

[87] James BJ , Gales MA, Gales BJ. Bumetanide for autism spectrum disorder in children: a review of randomized controlled trials. Ann Pharmacother 2019;53:537–544. 30501497 10.1177/1060028018817304

[88] Zhang L , Huang CC, Dai Y, Luo Q, Ji Y, Wang K, Deng S, Yu J, Xu M, Du X, Tang Y, Shen C, Feng J, Sahakian BJ, Lin CP, Li F. Symptom improvement in children with autism spectrum disorder following bumetanide administration is associated with decreased GABA/glutamate ratios. Transl Psychiatry 2020;10:9. 32066666 10.1038/s41398-020-0692-2 PMC7026137

[89] Du L , Shan L, Wang B, Li H, Xu Z, Staal WG., Jia F. A pilot study on the combination of applied behavior analysis and bumetanide treatment for children with autism. J Child Adolesc Psychopharmacol 2015;25:585–588. 26258842 10.1089/cap.2015.0045

[90] Fernell E , Gustafsson P, Gillberg C. Bumetanide for autism: open-label trial in six children. Acta Paediatric 2021;110:1548–1553. 10.1111/apa.15723 PMC8248373 33336381

[91] Feng JY , Li HH, Wang B, Shan L, Jia FY. Successive clinical application of Vitamin D and bumetanide in children with autism spectrum disorder: a case report. Medicine (Baltimore) 2020;99:e18661. 31914053 10.1097/MD.0000000000018661 PMC6959878

[92] Lemonnier E , Robin G, Degrez C, Tyzio R, Grandgeorge M, Ben-Ari Y. Treating fragile X syndrome with the diuretic bumetanide: a case report. Acta Paediatric 2013;102:e288–e290. 10.1111/apa.12235 23647528

[93] Lemonnier E , Lazartigues A, Ben-Ari Y. Treating schizophrenia with the diuretic bumetanide: a case report. Clin Neuropharmacol 2016;39:115–117. 26966887 10.1097/WNF.0000000000000136

[94] Akhondzadeh S , Mojtahedzadeh V, Mirsepassi GR, Moin M, Amini-Nooshabadi H, Kamalipour A. Diazoxide in the treatment of schizophrenia: novel application of potassium channel openers in the treatment of schizophrenia. J Clin Pharm Ther 2002;27:453–459. 12472985 10.1046/j.1365-2710.2002.00445.x

[95] Yildiz A , Nikodem M, Vieta E, Correll CU, Baldessarini RJ. A network meta-Analysis on comparative efficacy and all-cause discontinuation of antimanic treatments in acute bipolar mania. Psychol Med 2015;45:299–317. 25036226 10.1017/S0033291714001305

[96] Cipriani A , Saunders K, Attenburrow MJ, Stefaniak J, Panchal P, Stockton S, Lane TA, Tunbridge EM, Geddes JR, Harrison PJ. A systematic review of calcium channel antagonists in bipolar disorder and some considerations for their future development. Mol Psychiatry 2016;21:1324–1332. 27240535 10.1038/mp.2016.86 PMC5030455

[97] Burdick KE , Perez-Rodriguez M, Birnbaum R, Shanahan M, Larsen E, Harper C, Poskus J, Sklar P. A molecular approach to treating cognition in schizophrenia by calcium channel blockade: an open-label pilot study of the calcium-channel antagonist isradipine. Schizophr Res Cogn 2020;21:100180. 32455122 10.1016/j.scog.2020.100180 PMC7235642

[98] Vahdani B , Armani Kian A, Esmaeilzadeh A, Zenoozian S, Yousefi V, Mazloomzadeh S. Adjunctive raloxifene and isradipine improve cognitive functioning in patients with schizophrenia: a pilot study. J Clin Psychopharmacol 2020;40:457–463. 32796392 10.1097/JCP.0000000000001274

[99] Béné J , Richard F, Henon H, Bordet AM, Deplanque D, Lucas C, Girot M, Leys D, Abstracts BR. Impact of main antihypertensive and lipid lowering agents on cognitive decline in a cohort of stroke patients. Fundament Clin Pharmacol 2012;26:1–122.

[100] Climent M , Martínez-Castillo A, Rodríguez-Moldes J, Mota K, Botella P, Salar L, Moreno L. Poster Abstracts. Antihypertensive therapy associated with a lower risk of cognitive impairment. a community pharmacy study. Basic Clin Pharmacol Toxicol 2013;113:21–48.

[101] Boal AH , Smith DJ, McCallum L, Muir S, Touyz RM, Dominiczak AF, Padmanabhan S. Monotherapy with major antihypertensive drug classes and risk of hospital admissions for mood disorders. Hypertension 2016;68:1132–1138. 27733585 10.1161/HYPERTENSIONAHA.116.08188 PMC5058642

[102] Williams LJ , Pasco JA, Kessing L V, Quirk SE, Fernandes BS, Berk M. Angiotensin converting enzyme inhibitors and risk of mood disorders. Psychother Psychosom 2016;85:250–252. 27230871 10.1159/000444646

[103] Kessing L V , Rytgaard HC, Gerds TA, Berk M, Ekstrøm CT, Andersen PK. New drug candidates for depression – a nationwide population-based study. Acta Psychiatr Scand 2019;139:68–77. 30182363 10.1111/acps.12957

[104] Brownstein DJ , Salagre E, Köhler C, Stubbs B, Vian J, Pereira C, Chavarria V, Karmakar C, Turner A, Quevedo J, Carvalho AF, Berk M, Fernandes BS. Blockade of the angiotensin system improves mental health domain of quality of life: a meta-analysis of randomized clinical trials. Aust N Z J Psychiatry. 2018; 52:24–38. 28754072 10.1177/0004867417721654

[105] Lin SY , Lin CL, Lin CC, Hsu WH, Lin CD, Wang IK, Hsieh MH, Hsu CY, Kao CH. Association between angiotensin receptor blockers and suicide: nationwide population-based propensity score matching study. J Affect Disord 2020;276:815–821. 32738666 10.1016/j.jad.2020.07.106

[106] Fan X , Song X, Zhao M, Jarskog LF, Natarajan R, Shukair N, Freudenreich O, Henderson DC, Goff DC. The effect of adjunctive telmisartan treatment on psychopathology and cognition in patients with schizophrenia. Acta Psychiatr Scand 2017;136:465–472. 28851055 10.1111/acps.12799 PMC5630515

[107] Anderson C . More indirect evidence of potential neuroprotective benefits of angiotensin receptor blockers. J Hypertens 2010;28:429. 20160578 10.1097/HJH.0b013e3283371355

[108] Saxby BK , Harrington F, Wesnes KA, McKeith IG, Ford GA. Candesartan and cognitive decline in older patients with hypertension: a substudy of the SCOPE trial. Neurology 2008;70:1858–1866. 18458219 10.1212/01.wnl.0000311447.85948.78

[109] Callréus T , Agerskov Andersen U, Hallas J, Andersen M. Cardiovascular drugs and the risk of suicide: a nested case-control study. Eur J Clin Pharmacol 2007;63:591–596. 17468865 10.1007/s00228-007-0293-5

[110] Erzengin M , Bilen C, Ergun A, Gençer N. Antipsychotic agents screened as human carbonic anhydrase I and II inhibitors. Arc Physiol Biochem 2014;120:29–33. 10.3109/13813455.2013.863359 24289818

[111] Newton TF , de La Garza R, Brown G, Kosten TR, Mahoney JJ, Haile CN. Noradrenergic α 1 receptor antagonist treatment attenuates positive subjective effects of cocaine in humans: a randomized trial. PLoS ONE 2012;7:e30854. 22319592 10.1371/journal.pone.0030854 PMC3272014

[112] Shorter D , Lindsay JA, Kosten TR. The alpha-1 adrenergic antagonist doxazosin for treatment of cocaine dependence: a pilot study. Drug Alcohol Depend 2013;131:66–70. 23306096 10.1016/j.drugalcdep.2012.11.021 PMC3655111

[113] Zhang X , Nielsen DA, Domingo CB, Shorter DI, Nielsen EM, Kosten TR. Pharmacogenetics of dopamine β-hydroxylase in cocaine dependence therapy with doxazosin. Addict Biol 2019;24:531–538. 29498170 10.1111/adb.12611 PMC6119656

[114] Shorter DI , Zhang X, Domingo CB, Nielsen EM, Kosten TR, Nielsen DA. Doxazosin treatment in cocaine use disorder: pharmacogenetic response based on an alpha-1 adrenoreceptor subtype D genetic variant. Am J Drug Alcohol Abuse 2020;46:184–193. 31914324 10.1080/00952990.2019.1674864

[115] Frishman WH , Lazar EJ, Gorodokin G. Pharmacokinetic optimisation of therapy with β-adrenergic blocking agents. Clin Pharmacokinet 1991;20:311–318. 1674683 10.2165/00003088-199120040-00005

[116] Liu Y , Zhou X, Zhu D, Chen J, Qin B, Zhang Y, Wang X, Yang D, Meng H, Luo Q, Xie P. Is pindolol augmentation effective in depressed patients resistant to selective serotonin reuptake inhibitors? A systematic review and meta-analysis. Hum Psychopharmacol 2015;30:132–142. 25689398 10.1002/hup.2465

[117] Ranchord AM , Spertus JA, Buchanan DM, Gosch KL, Chan PS. Initiation of β-blocker therapy and depression after acute myocardial infarction. Am Heart J 2016;174:37–42. 26995368 10.1016/j.ahj.2015.11.018 PMC4802859

[118] Kim C , Duan L, Phan DQ, Lee MS. Frequency of utilization of beta blockers in patients with heart failure and depression and their effect on mortality. Am J Cardiol 2019;124:746–750. 31277789 10.1016/j.amjcard.2019.05.054

[119] Agustini B , Mohebbi M, Woods RL, McNeil JJ, Nelson MR, Shah RC, Murray AM, Ernst ME, Reid CM, Tonkin A, Lockery JE, Berk M; ASPREE Investigator Group. The association of antihypertensive use and depressive symptoms in a large older population with hypertension living in Australia and the United States: a cross-sectional study. J Hum Hypertens 2020;34:787–794. 32001828 10.1038/s41371-020-0303-y PMC7390661

[120] Johnson JA , Zineh I, Puckett BJ, McGorray SP, Yarandi HN, Pauly DF. β1-adrenergic receptor polymorphisms and antihypertensive response to metoprolol. Clin Pharmacol Ther 2003;74:44–52. 12844134 10.1016/S0009-9236(03)00068-7

[121] Liu J , Liu Z-Q, Tan Z-R, Chen X-P, Wang L-S, Zhou G, Zhou H-H. Gly389Arg polymorphism of β1-adrenergic receptor is associated with the cardiovascular response to metoprolol. Clin Pharmacol Ther 2003;74:372–379. 14534524 10.1016/S0009-9236(03)00224-8

[122] Zill P , Baghai TC, Engel R, Zwanzger P, Schüle C, Minov C, Behrens S, Bottlender R, Jäger M, Rupprecht R, Möller HJ, Ackenheil M, Bondy B. Beta-1-adrenergic receptor gene in major depression: Influence on antidepressant treatment response. Am J Med Genet B Neuropsychiatr Genet 2003;120:85–89. 10.1002/ajmg.b.20017 12815745

[123] Amare AT , Schubert KO, Klingler-Hoffmann M, Cohen-Woods S, Baune BT. The genetic overlap between mood disorders and cardiometabolic diseases: a systematic review of genome wide and candidate gene studies. Transl Psychiatry 2017;7:e1007. 28117839 10.1038/tp.2016.261 PMC5545727

[124] Keiser MJ , Setola V, Irwin JJ, Laggner C, Abbas AI, Hufeisen SJ, Jensen NH, Kuijer MB, Matos RC, Tran TB, Whaley R, Glennon RA, Hert J, Thomas KL, Edwards DD, Shoichet BK, Roth BL. Predicting new molecular targets for known drugs. Nature 2009;462:175–181. 19881490 10.1038/nature08506 PMC2784146

[125] Thomas KLH , Ellingrod VL, Bishop JR, Keiser MJ. A pilot study of the pharmacodynamic impact of SSRI drug selection and beta-1 receptor genotype (ADRB1) on cardiac vital signs in depressed patients: a novel pharmacogenetic approach. Psychopharmacol Bull 2010;43:11–22. 20581797 PMC3807824

[126] Hill AS , Ben-Shahar Y. The synaptic action of degenerin/epithelial sodium channels. Channels 2018;12:262–275. 30001175 10.1080/19336950.2018.1495006 PMC6986788

[127] Dzhala VI , Talos DM, Sdrulla DA, Brumback AC, Mathews GC, Benke TA, Delpire E, Jensen FE, Staley KJ. NKCC1 transporter facilitates seizures in the developing brain. Nat Med 2005;11:1205–1213. 16227993 10.1038/nm1301

[128] Ben-Ari Y , Gaiarsa JL, Tyzio R, Khazipov R. GABA: a pioneer transmitter that excites immature neurons and generates primitive oscillations. Physiol Rev 2007;87:1215–1284. 17928584 10.1152/physrev.00017.2006

[129] Casini A , Caccia S, Scozzafava A, Supuran CT. Carbonic anhydrase activators. The selective serotonin reuptake inhibitors fluoxetine, sertraline and citalopram are strong activators of isozymes I and II. Bioorg Med Chem Lett 2003;13:2765–2768. 12873510 10.1016/s0960-894x(03)00507-9

[130] Ozsoy HZ . Anticonvulsant effects of carbonic anhydrase inhibitors: the enigmatic link between carbonic anhydrases and electrical activity of the brain. Neurochem Res 2021;46:2783–2799. 34226984 10.1007/s11064-021-03390-2

[131] Sun MK , Alkon DL. Carbonic anhydrase gating of attention: memory therapy and enhancement. Trends Pharmacol Sci 2002;23:83–89. 11830265 10.1016/s0165-6147(02)01899-0

[132] Song YR , Wu B, Yang YT, Chen J, Zhang LJ, Zhang ZW, Shi HY, Huang CL, Pan JX, Xie P. Specific alterations in plasma proteins during depressed, manic, and euthymic states of bipolar disorder. Braz J Med Biol Res 2015;48:973–982. 26375446 10.1590/1414-431X20154550 PMC4671523

[133] Johnston-Wilson NL , Sims CD, Hofmann JP, Anderson L, Shore AD, Torrey EF, Yolken RH. Disease-specific alterations in frontal cortex brain proteins in schizophrenia, bipolar disorder, and major depresssive disorder. Mol Psychiatry 2000;5:142–149. 10822341 10.1038/sj.mp.4000696

[134] Hayes SG . Acetazolamide in bipolar affective disorders. Ann Clin Psychiatry 1994;6:91–98. 7804393 10.3109/10401239409148987

[135] Yellepeddi V , Sayre C, Burrows A, Watt K, Davies S, Strauss J, Battaglia M. Stability of extemporaneously compounded amiloride nasal spray. PLoS One 2020;15:e0232435. 32649677 10.1371/journal.pone.0232435 PMC7351165

[136] Chen J , Wang ZZ, Zhang S, Chu S-F, Mou Z, Chen N-H. The effects of glucocorticoids on depressive and anxiety-like behaviors, mineralocorticoid receptor-dependent cell proliferation regulates anxiety-like behaviors. Behav Brain Res 2019;362:288–298. 30654121 10.1016/j.bbr.2019.01.026

[137] Juruena MF , Pariante CM, Papadopoulos AS, Poon L, Lightman S, Cleare AJ. The role of mineralocorticoid receptor function in treatment-resistant depression. J Psychopharmacol 2013;27:1169–1179. 23904409 10.1177/0269881113499205

[138] Makhijani VH , Van Voorhies K, Besheer J. The mineralocorticoid receptor antagonist spironolactone reduces alcohol self-administration in female and male rats. Pharmacol Biochem Behav 2018;175:10–18. 30171933 10.1016/j.pbb.2018.07.011 PMC6240486

[139] Wehr MC , Hinrichs W, Brzózka MM, Unterbarnscheidt T, Herholt A, Wintgens JP, Papiol S, Soto-Bernardini MC, Kravchenko M, Zhang M, Nave KA, Wichert SP, Falkai P, Zhang W, Schwab MH, Rossner MJ. Spironolactone is an antagonist of NRG 1- ERBB 4 signaling and schizophrenia-relevant endophenotypes in mice. EMBO Mol Med 2017;9:1448–1462. 28743784 10.15252/emmm.201707691 PMC5653977

[140] Hasan A , Roeh A, Leucht S, Langguth B, Hansbauer M, Oviedo-Salcedo T, Kirchner SK, Papazova I, Löhrs L, Wagner E, Maurus I, Strube W, Rossner MJ, Wehr MC, Bauer I, Heres S, Leucht C, Kreuzer PM, Zimmermann S, Schneider-Axmann T, Görlitz T, Karch S, Egert-Schwender S, Schossow B, Rothe P, Falkai P. Add-on spironolactone as antagonist of the NRG1-ERBB4 signaling pathway for the treatment of schizophrenia: study design and methodology of a multicenter randomized, placebo-controlled trial. Contemp Clin Trials Commun 2020;17:100537. 32072071 10.1016/j.conctc.2020.100537 PMC7013159

[141] Liu R , Wang J, Liang S, Zhang G, Yang X. Role of NKCC1 and KCC2 in epilepsy: from expression to function. Front Neurol 2020;10:1407. 32010056 10.3389/fneur.2019.01407 PMC6978738

[142] Löscher W , Puskarjov M, Kaila K. Cation-chloride cotransporters NKCC1 and KCC2 as potential targets for novel antiepileptic and antiepileptogenic treatments. Neuropharmacology 2013;69:62–74. 22705273 10.1016/j.neuropharm.2012.05.045

[143] Ben-Ari Y . NKCC1 chloride importer antagonists attenuate many neurological and psychiatric disorders. Trends Neurosci 2017;40:536–554. 28818303 10.1016/j.tins.2017.07.001

[144] Krystal AD , Sutherland J, Hochman DW. Loop diuretics have anxiolytic effects in rat models of conditioned anxiety. PLoS One 2012;7:e35417. 22514741 10.1371/journal.pone.0035417 PMC3325958

[145] Römermann K , Fedrowitz M, Hampel P, Kaczmarek E, Töllner K, Erker T, Sweet DH, Löscher W. Multiple blood-brain barrier transport mechanisms limit bumetanide accumulation, and therapeutic potential, in the mammalian brain. Neuropharmacology 2017;117:182–194. 28192112 10.1016/j.neuropharm.2017.02.006

[146] Löscher W , Kaila K. Reply to the commentary by Ben-Ari and Delpire: bumetanide and neonatal seizures: fiction versus reality. Epilepsia 2021;62:941–946. 33764535 10.1111/epi.16866

[147] Töllner K , Brandt C, Töpfer M, Brunhofer G, Erker T, Gabriel M, Feit PW, Lindfors J, Kaila K, Löscher W. A novel prodrug-based strategy to increase effects of bumetanide in epilepsy. Ann Neurol 2014;75:550–562. 24615913 10.1002/ana.24124

[148] Erker T , Brandt C, Töllner K, Schreppel P, Twele F, Schidlitzki A, Löscher W. The bumetanide prodrug BUM5, but not bumetanide, potentiates the antiseizure effect of phenobarbital in adult epileptic mice. Epilepsia 2016;57:698–705. 26921222 10.1111/epi.13346

[149] Huang H , Bhuiyan MIH, Jiang T, Song S, Shankar S, Taheri T, Li E, Schreppel P, Hintersteininger M, Yang SS, Lin SH, Molyneaux BJ, Zhang Z, Erker T, Sun D. A novel Na+-K+-Cl- cotransporter 1 inhibitor STS66∗ reduces brain damage in mice after ischemic stroke. Stroke 2019;50:1021–1025. 30862257 10.1161/STROKEAHA.118.024287 PMC6608592

[150] Auer T , Schreppel P, Erker T, Schwarzer C. Functional characterization of novel bumetanide derivatives for epilepsy treatment. Neuropharmacology 2020;162:107754. 31476353 10.1016/j.neuropharm.2019.107754

[151] Lemonnier E , Ben-Ari Y. The diuretic bumetanide decreases autistic behaviour in five infants treated during 3 months with no side effects. Acta Paediatric 2010;99:1885–1888. 10.1111/j.1651-2227.2010.01933.x 20608900

[152] Lemonnier E , Degrez C, Phelep M, Tyzio R, Josse F, Grandgeorge M, Hadjikhani N, Ben-Ari Y. A randomised controlled trial of bumetanide in the treatment of autism in children. Transl Psychiatry 2012;2:e202. 23233021 10.1038/tp.2012.124 PMC3565189

[153] Lemonnier E , Villeneuve N, Sonie S, Serret S, Rosier A, Roue M, Brosset P, Viellard M, Bernoux D, Rondeau S, Thummler S, Ravel D, Ben-Ari Y. Effects of bumetanide on neurobehavioral function in children and adolescents with autism spectrum disorders. Transl Psychiatry 2017;7:e1056. 28291262 10.1038/tp.2017.10 PMC5416661

[154] Hadjikhani N , Zürcher NR, Rogier O, Ruest T, Hippolyte L, Ben-Ari Y, Lemonnier E. Improving emotional face perception in autism with diuretic bumetanide: a proof-of-concept behavioral and functional brain imaging pilot study. Autism 2015;19:149–157. 24343334 10.1177/1362361313514141

[155] Hadjikhani N , Åsberg Johnels J, Lassalle A, Zürcher NR, Hippolyte L, Gillberg C, Lemonnier E, Ben-Ari Y. Bumetanide for autism: more eye contact, less amygdala activation. Sci Rep 2018;8:3602. 29483603 10.1038/s41598-018-21958-x PMC5827728

[156] Vergeau J-C . Servier and Neurochlore announce the main results of the two phase 3 clinical studies assessing bumetanide in the treatment of Autism Spectrum Disorders in children and adolescents. 2021:1–3. Press release.

[157] Wang T , Shan L, Miao C, Xu Z, Jia F. Treatment effect of bumetanide in children with autism spectrum disorder: a systematic review and meta-analysis. Front Psychiatry 2021;12:751575. 34867539 10.3389/fpsyt.2021.751575 PMC8634163

[158] Ben-Ari Y , Lemonnier E. Using bumetanide to treat autism appears promising but further clinical trials are needed to confirm this approach. Acta Paediatric 2021;110:1395–1397. 10.1111/apa.15747 33484191

[159] Sprengers JJ , van Andel DM, Zuithoff NPA, Zuithoff NPA, Keijzer-Veen MG, Schulp AJA, Scheepers FE, Lilien MR, Oranje B, Bruining H. Bumetanide for core symptoms of autism spectrum disorder (BAMBI): a single center, double-blinded, participant-randomized, placebo-controlled, phase-2 superiority trial. J Am Acad Child Adolesc Psychiatry 2021;60:865–876. 32730977 10.1016/j.jaac.2020.07.888

[160] Striessnig J , Ortner N, Pinggera A. Pharmacology of L-type calcium channels: novel drugs for old targets? Curr Mol Pharmacol 2015;8:110–122. 25966690 10.2174/1874467208666150507105845 PMC5384371

[161] Bootman MD . Calcium signaling. Cold Spring Harb Perspect Biol 2012;4:a011171. 22751152 10.1101/cshperspect.a011171 PMC3385957

[162] Dolphin AC . Voltage-gated calcium channels and their auxiliary subunits: physiology and pathophysiology and pharmacology. J Physiol 2016;594:5369–5390. 27273705 10.1113/JP272262 PMC5043047

[163] Campiglio M , Flucher BE. The role of auxiliary subunits for the functional diversity of voltage-gated calcium channels. J Cell Physiol 2015;230:2019–2031. 25820299 10.1002/jcp.24998 PMC4672716

[164] Zamponi GW , Striessnig J, Koschak A, Dolphin AC. The physiology, pathology, and pharmacology of voltage-gated calcium channels and their future therapeutic potential. Pharmacol Rev 2015;67:821–870. 26362469 10.1124/pr.114.009654 PMC4630564

[165] Striessnig J , Koschak A. Exploring the function and pharmacotherapeutic potential of voltage-gated Ca2 + channels with gene Knockout models. Channels 2008;2:233–251. 18719397 10.4161/chan.2.4.5847

[166] Sinnegger-Brauns MJ , Huber IG, Koschak A, Wild C, Obermair GJ, Einzinger U, Hoda JC, Sartori SB, Striessnig J. Expression and 1,4-dihydropyridine-binding properties of brain L-type calcium channel isoforms. Mol Pharmacol 2009;75:407–414. 19029287 10.1124/mol.108.049981

[167] Striessnig J , Pinggera A, Kaur G, Bock G, Tuluc P. L-type Ca2 + channels in heart and brain. Wiley Interdiscip Rev Membr Transp Signal 2014;3:15–38. 24683526 10.1002/wmts.102 PMC3968275

[168] Berger SM , Bartsch D. The role of L-type voltage-gated calcium channels Cav1.2 and Cav1.3 in normal and pathological brain function. Cell Tissue Res 2014;357:463–476. 24996399 10.1007/s00441-014-1936-3

[169] White JA , McKinney BC, John MC, Powers PA, Kamp TJ, Murphy GG. Conditional forebrain deletion of the L-type calcium channel Ca V1.2 disrupts remote spatial memories in mice. Learn Mem 2008;15:1–5. 18174367 10.1101/lm.773208

[170] Moosmang S , Haider N, Klugbauer N, Adelsberger H, Langwieser N, Müller J, Stiess M, Marais E, Schulla V, Lacinova L, Goebbels S, Nave KA, Storm DR, Hofmann F, Kleppisch T. Role of hippocampal Cav1.2 Ca2 + channels in NMDA receptor-independent synaptic plasticity and spatial memory. J Neurosci 2005;25:9883–9892. 16251435 10.1523/JNEUROSCI.1531-05.2005 PMC6725564

[171] Oh MM , Oliveira FA, Disterhoft JF. Learning and aging related changes in intrinsic neuronal excitability. Front Aging Neurosci 2010;2:2. 20552042 10.3389/neuro.24.002.2010 PMC2874400

[172] Bauer EP , Schafe GE, LeDoux JE. NMDA receptors and L-type voltage-gated calcium channels contribute to long-term potentiation and different components of fear memory formation in the lateral amygdala. J Neurosci 2002;22:5239–5249. 12077219 10.1523/JNEUROSCI.22-12-05239.2002 PMC6757716

[173] McKinney BC , Murphy GG. The L-type voltage-gated calcium channel Cav1.3 mediates consolidation, but not extinction, of contextually conditioned fear in mice. Learn Mem 2006;13:584–589. 17015855 10.1101/lm.279006 PMC1783612

[174] Giordano TP , Tropea TF, Satpute SS, Sinnegger-Brauns MJ, Striessnig J, Kosofsky BE, Rajadhyaksha AM. Molecular switch from L-type Cav1.3 to Cav1.2 Ca 2 + channel signaling underlies long-term psychostimulant-induced behavioral and molecular plasticity. J Neurosci 2010;30:17051–17062. 21159975 10.1523/JNEUROSCI.2255-10.2010 PMC3077109

[175] Espinosa-Parrilla JF , Martínez-Moreno M, Gasull X, Mahy N, Rodríguez MJ. The L-type voltage-gated calcium channel modulates microglial pro-inflammatory activity. Mol Cell Neurosci 2015;64:104–115. 25497271 10.1016/j.mcn.2014.12.004

[176] Wang Y , Tang S, Harvey KE, Salyer AE, Li TA, Rantz EK, Lill MA, Hockerman GH. Molecular determinants of the differential modulation of Ca v 1.2 and Ca v 1.3 by nifedipine and FPL 64176. Mol Pharmacol 2018;94:973–983. 29980657 10.1124/mol.118.112441 PMC11033928

[177] Ganouni S E , Tazi A, Hakkou F. Potential serotonergic interactions with the anxiolytic-like effects of calcium channel antagonists. Pharmacol Biochem Behav 1998;60:365–369. 9632218 10.1016/s0091-3057(98)00014-8

[178] Srivastava SK , Nath C. The differential effects of calcium channel blockers in the behavioural despair test in mice. Pharmacol Res 2000;42:293–297. 10987986 10.1006/phrs.2000.0696

[179] Cohen C , Perrault G, Sanger DJ. Assessment of the antidepressant-like effects of L-type voltage-dependent channel modulators. Behav Pharmacol 1997;8:629–638. 9832975 10.1097/00008877-199711000-00019

[180] Shinnick-Gallagher P , McKernan MG, Xie J, Zinebi F. L-type voltage-gated calcium channels are involved in the in vivo and in vitro expression of fear conditioning. Ann N Y Acad Sci 2003;985:135–149. 12724155 10.1111/j.1749-6632.2003.tb07078.x

[181] Yoshizawa K , Nakashima K, Tabuchi M, Okumura A, Nakatake Y, Yamada M, Tsuneoka Y, Higashi T. Benzothiazepines diltiazem and JTV-519, exert an anxiolytic-like effect via neurosteroid biosynthesis in mice. J Pharmacol Sci 2020;143:234–237. 32249061 10.1016/j.jphs.2020.03.003

[182] Chan CS , Guzman JN, Ilijic E, Mercer JN, Rick C, Tkatch T, Meredith GE, Surmeier DJ. “Rejuvenation” protects neurons in mouse models of Parkinson’s disease. Nature 2007;447:1081–1086. 17558391 10.1038/nature05865

[183] Ilijic E , Guzman JN, Surmeier DJ. The L-type channel antagonist isradipine is neuroprotective in a mouse model of Parkinson’s disease. Neurobiol Dis 2011;43:364–371. 21515375 10.1016/j.nbd.2011.04.007 PMC3235730

[184] Shibasaki M , Kurokawa K, Ohkuma S. Upregulation of L-type Cav1 channels in the development of psychological dependence. Synapse 2010;64:440–444. 20169575 10.1002/syn.20745

[185] Michaluk J , Karolewicz B, Antkiewicz-Michaluk L, Vetulani J. Effects of various Ca2 + channel antagonists on morphine analgesia, tolerance and dependence, and on blood pressure in the rat. Eur J Pharmacol 1998;352:189–197. 9716354 10.1016/s0014-2999(98)00373-2

[186] Little HJ . L-type calcium channel blockers: a potential novel therapeutic approach to drug dependence. Pharmacol Rev 2021;73:127–154. 34663686 10.1124/pharmrev.120.000245

[187] Alboghobeish S , Naghizadeh B, Kheirollah A, Ghorbanzadeh B, Mansouri MT. Fluoxetine increases analgesic effects of morphine, prevents development of morphine tolerance and dependence through the modulation of L-type calcium channels expression in mice. Behav Brain Res 2019;361:86–94. 30550947 10.1016/j.bbr.2018.12.020

[188] Ferreira MAR , O’Donovan MC, Meng YA, Ones IR, Ruderfer DM, Jones L, Fan J, Kirov G, Perlis RH, Green EK, Smoller JW, Grozeva D, Stone J, Nikolov I, Chambert K, Hamshere ML, Nimgaonkar VL, Moskvina V, Thase ME, Caesar S, Sachs GS, Franklin J, Gordon-Smith K, Ardlie KG, Gabriel SB, Fraser C, Blumenstiel B, Defelice M, Breen G, Gill M, Morris DW, Elkin A, Muir WJ, McGhee KA, Williamson R, MacIntyre DJ, MacLean AW, St CD, Robinson M, Van Beck M, Pereira AC, Kandaswamy R, McQuillin A, Collier DA, Bass NJ, Young AH, Lawrence J, Ferrier IN, Anjorin A, Farmer A, Curtis D, Scolnick EM, McGuffin P, Daly MJ, Corvin AP, Holmans PA, Blackwood DH, Gurling HM, Owen MJ, Purcell SM, Sklar P, Craddock N; Wellcome Trust Case Control Consortium. Collaborative genome-wide association analysis supports a role for ANK3 and CACNA1C in bipolar disorder. Nat Gen 2008;40:1056–1058. 10.1038/ng.209 PMC2703780 18711365

[189] Cross-Disorder Group of the Psychiatric Genomics Consortium . Identification of risk loci with shared effects on five major psychiatric disorders: a genome-wide analysis. Lancet 2013;381:1371–1379. 23453885 10.1016/S0140-6736(12)62129-1 PMC3714010

[190] Sklar P , Smoller JW, Fan J, Perlis RH, Chambert K, Nimgaonkar VL, McQueen MB, Faraone SV, Kirby A, de Bakker PI, Ogdie MN, Thase ME, Sachs GS, Todd-Brown K, Gabriel SB, Sougnez C, Gates C, Blumenstiel B, Defelice M, Ardlie KG, Franklin J, Muir WJ, McGhee KA, MacIntyre DJ, McLean A, VanBeck M, McQuillin A, Bass NJ, Robinson M, Lawrence J, Anjorin A, Curtis D, Scolnick EM, Daly MJ, Blackwood DH, Gurling HM, Purcell SM. Whole-genome association study of bipolar disorder. Mol Psychiatry 2008;13:558–569. 18317468 10.1038/sj.mp.4002151 PMC3777816

[191] Casamassima F , Huang J, Fava M, Sachs GS, Smoller JW, Cassano GB, Lattanzi L, Fagerness J, Stange JP, Perlis RH. Phenotypic effects of a bipolar liability gene among individuals with major depressive disorder. Am J Med Genet B Neuropsychiatr Genet 2010;153:303–309. 10.1002/ajmg.b.30962 19388002

[192] Schizophrenia Working Group of the Psychiatric Genomics Consortium . Biological insights from 108 schizophrenia-associated genetic loci. Nature 2014;511:421–427. 25056061 10.1038/nature13595 PMC4112379

[193] Liao X , Liao X, Li Y. Genetic associations between voltage-gated calcium channels and autism spectrum disorder: a systematic review. Mol Brain 2020;13:96. 32571372 10.1186/s13041-020-00634-0 PMC7310353

[194] Byrne EM , Gehrman PR, Medland SE, Nyholt DR, Heath AC, Madden PA, Hickie IB, Van Duijn CM, Henders AK, Montgomery GW, Martin NG, Wray NR; Chronogen Consortium. A genome-wide association study of sleep habits and insomnia. Am J Med Genet B Neuropsychiatr Genet 2013;162:439–451. 10.1002/ajmg.b.32168 PMC4083458 23728906

[195] Parsons MJ , Lester KJ, Barclay NL, Nolan PM, Eley TC, Gregory AM. Replication of genome-wide association studies (GWAS) loci for sleep in the British G1219 cohort. Am J Med Genet B Neuropsychiatr Genet 2013;162:431–438. 10.1002/ajmg.b.32106 23780892

[196] Van Oort S , Beulens JWJ, Van Ballegooijen AJ, Grobbee DE, Larsson SC. Association of cardiovascular risk factors and lifestyle behaviors with hypertension: a Mendelian randomization study. Hypertension 2020;76:1971–1979. 33131310 10.1161/HYPERTENSIONAHA.120.15761

[197] Andrade A , Brennecke A, Mallat S, Brown J, Gomez-Rivadeneira J, Czepiel N, Londrigan L. Genetic associations between voltage-gated calcium channels and psychiatric disorders. Int J Mol Sci 2019;20:3537. 31331039 10.3390/ijms20143537 PMC6679227

[198] Yoshimizu T , Pan JQ, Mungenast AE, Madison JM, Su S, Ketterman J, Ongur D, McPhie D, Cohen B, Perlis R, Tsai LH. Functional implications of a psychiatric risk variant within CACNA1C in induced human neurons. Mol Psychiatry 2015;20:162–169. 25403839 10.1038/mp.2014.143 PMC4394050

[199] Starnawska A , Demontis D, Pen A, Hedemand A, Nielsen AL, Staunstrup NH, Grove J, Als TD, Jarram A, O'Brien NL, Mors O, McQuillin A, Børglum AD, Nyegaard M. CACNA1C hypermethylation is associated with bipolar disorder. Transl Psychiatry 2016;6:e831. 27271857 10.1038/tp.2016.99 PMC4931616

[200] Balaraman Y , Lahiri DK, Nurnberger JI. Variants in ion channel genes link phenotypic features of bipolar illness to specific neurobiological process domains. Mol Neuropsychiatry 2015;1:23–35. 27602355 10.1159/000371886 PMC4996004

[201] Soeiro-De-Souza MG , Lafer B, Moreno RA, Nery FG, Chile T, Chaim K, da Costa Leite C, Machado-Vieira R, Otaduy MC, Vallada H. The CACNA1C risk allele rs1006737 is associated with age-related prefrontal cortical thinning in bipolar i disorder. Transl Psychiatry 2017;7:e1086. 28398341 10.1038/tp.2017.57 PMC5416698

[202] Atkinson LZ , Colbourne L, Smith A, Harmer CH, Nobre AC, Rendell J, Jones H, Hinds C, Mould A, Tunbridge EM, Cipriani A, Geddes JR, Saunders KEA, Harrison PJ. The Oxford study of calcium channel antagonism, cognition, mood instability and sleep (OxCaMS): study protocol for a randomised controlled, experimental medicine study. Trials 2019;20:120. 30755265 10.1186/s13063-019-3175-0 PMC6373140

[203] Hayes JF , Lundin A, Wicks S, Lewis G, Wong ICK, Osborn DPJ, Dalman C. Association of hydroxylmethyl glutaryl coenzyme A reductase inhibitors, L-type calcium channel antagonists, and biguanides with rates of psychiatric hospitalization and self-harm in individuals with serious mental illness. JAMA Psychiatry 2018;76:382–390. 10.1001/jamapsychiatry.2018.3907 PMC6450278 30624557

[204] Kessing LV , Rytgaard HC, Ekstrøm CT, Torp-Pedersen C, Berk M, Gerds TA. Antihypertensive drugs and risk of depression: a nationwide population-based study. Hypertension 2020;76:1263–1279. 32829669 10.1161/HYPERTENSIONAHA.120.15605

[205] Paul M , Mehr AP, Kreutz R. Physiology of local renin-angiotensin systems. Physiol Rev 2006;86:747–803. 16816138 10.1152/physrev.00036.2005

[206] Wright JW , Harding JW. The brain renin-angiotensin system: a diversity of functions and implications for CNS diseases. Pflugers Arch 2013;465:133–151. 22535332 10.1007/s00424-012-1102-2

[207] Vadhan JD , Speth RC. The role of the brain renin-angiotensin system (RAS) in mild traumatic brain injury (TBI). Pharmacol Ther 2021;218:107684. 32956721 10.1016/j.pharmthera.2020.107684

[208] Loera-Valencia R , Eroli F, Garcia-Ptacek S, Maioli S. Brain renin–angiotensin system as novel and potential therapeutic target for Alzheimer’s disease. Int J Mol Sci 2021;22:10139. 34576302 10.3390/ijms221810139 PMC8468637

[209] Cosarderelioglu C , Nidadavolu LS, George CJ, Oh ES, Bennett DA, Walston JD, Abadir PM. Brain renin–angiotensin system at the intersect of physical and cognitive frailty. Front Neurosci 2020;14:586314. 33117127 10.3389/fnins.2020.586314 PMC7561440

[210] Nakagawa P , Gomez J, Grobe JL, Sigmund CD. The renin-angiotensin system in the central nervous system and its role in blood pressure regulation. Curr Hypertens Rep 2020;22:7. 31925571 10.1007/s11906-019-1011-2 PMC7101821

[211] Bodiga VL , Bodiga S. Renin angiotensin system in cognitive function and dementia. Asian J Neurosci 2013;2013:1–18.

[212] Deschepper CF , Bouhnik J, Ganong WF. Colocalization of angiotensinogen and glial fibrillary acidic protein in astrocytes in rat brain. Brain Res 1986;374:195–198. 3521789 10.1016/0006-8993(86)90411-7

[213] Grobe JL , Xu D, Sigmund CD. An intracellular renin-angiotensin system in neurons: fact, hypothesis, or fantasy. Physiology 2008;23:187–193. 18697992 10.1152/physiol.00002.2008 PMC2538674

[214] Elased KM , Cunha TS, Marcondes FK, Morris M. Brain angiotensin-converting enzymes: role of angiotensin-converting enzyme 2 in processing angiotensin II in mice. Exp Physiol 2008;93:665–675. 18263657 10.1113/expphysiol.2007.040311 PMC7197900

[215] Labandeira-Garcia JL , Rodríguez-Perez AI, Garrido-Gil P, Rodriguez-Pallares J, Lanciego JL, Guerra MJ. Brain renin-angiotensin system and microglial polarization: implications for aging and neurodegeneration. Front Aging Neurosci 2017;9:129. 28515690 10.3389/fnagi.2017.00129 PMC5413566

[216] Lenkei Z , Palkovits M, Corvol P, Llorens-Cortes C. Distribution of angiotensin type-1 receptor messenger RNA expression in the adult rat brain. Neuroscience 1997;82:827–841. 10.1016/s0306-4522(97)00328-x 9483539

[217] Crozier RA , Ajit SK, Kaftan EJ, Pausch MH. MrgD activation inhibits KCNQ/M-currents and contributes to enhanced neuronal excitability. J Neurosci 2007;27:4492–4496. 17442834 10.1523/JNEUROSCI.4932-06.2007 PMC6672314

[218] Costa-Besada MA , Valenzuela R, Garrido-Gil P, Villar-Cheda B, Parga JA, Lanciego JL, Labandeira-Garcia JL. Paracrine and intracrine angiotensin 1-7/Mas receptor axis in the substantia nigra of rodents, monkeys, and humans. Mol Neurobiol 2018;55:5847–5867. 29086247 10.1007/s12035-017-0805-y PMC7102204

[219] Chai SY , Bastias MA, Clune EF, Matsacos DJ, Mustafa T, Lee JH, McDowall SG, Paxinos G, Mendelsohn FA, Albiston AL. Distribution of angiotensin IV binding sites (AT4 receptor) in the human forebrain, midbrain and pons as visualised by in vitro receptor autoradiography. J Chem Neuroanat 2000;20:339–348. 11207430 10.1016/s0891-0618(00)00112-5

[220] Ahmed HA , Ishrat T, Pillai B, Bunting KM, Patel A, Vazdarjanova A, Waller JL, Arbab AS, Ergul A, Fagan SC. Role of angiotensin system modulation on progression of cognitive impairment and brain MRI changes in aged hypertensive animals – a randomized double- blind pre-clinical study. Behav Brain Res 2018;346:29–40. 29229547 10.1016/j.bbr.2017.12.007 PMC5866136

[221] Zhang TL , Fu JL, Geng Z, Yang JJ, Sun XJ. The neuroprotective effect of losartan through inhibiting AT1/ASK1/MKK4/JNK3 pathway following cerebral I/R in rat hippocampal CA1 region. CNS Neurosci Ther 2012;18:981–987. 23095236 10.1111/cns.12015 PMC6493444

[222] Guimond MO , Gallo-Payet N. The angiotensin II type 2 receptor in brain functions: an update. Int J Hypertens 2012;2012:351758. 23320146 10.1155/2012/351758 PMC3540774

[223] Jackson L , Eldahshan W, Fagan SC, Ergul A. Within the brain: the renin angiotensin system. Int J Mol Sci 2018;19:876. 29543776 10.3390/ijms19030876 PMC5877737

[224] Valenzuela R , Costa-Besada MA, Iglesias-Gonzalez J, Perez-Costas E, Villar-Cheda B, Garrido-Gil P, Melendez-Ferro M, Soto-Otero R, Lanciego JL, Henrion D, Franco R, Labandeira-Garcia JL. Mitochondrial angiotensin receptors in dopaminergic neurons. Role in cell protection and aging-related vulnerability to neurodegeneration. Cell Death Dis 2016;7:e2427. 27763643 10.1038/cddis.2016.327 PMC5133991

[225] Forrester SJ , Booz GW, Sigmund CD, Coffman TM, Kawai T, Rizzo V, Scalia R, Eguchi S. Angiotensin II signal transduction: an update on mechanisms of physiology and pathophysiology. Physiol Rev 2018;98:1627–1738. 29873596 10.1152/physrev.00038.2017 PMC6335102

[226] Garrido AM , Griendling KK. NADPH oxidases and angiotensin II receptor signaling. Mol Cell Endocrinol 2009;302:148–158. 19059306 10.1016/j.mce.2008.11.003 PMC2835147

[227] Labandeira-García JL , Garrido-Gil P, Rodriguez-Pallares J, Valenzuela R, Borrajo A, Rodríguez-Perez AI. Brain renin-angiotensin system and dopaminergic cell vulnerability. Front Neuroanat 2014;8:67. 25071471 10.3389/fnana.2014.00067 PMC4086395

[228] Guimond MO , Gallo-Payet N. How does angiotensin AT2 receptor activation help neuronal differentiation and improve neuronal pathological situations? Front Endocrinol 2012;3:164. 10.3389/fendo.2012.00164 PMC3525946 23267346

[229] Côté F , Do TH, Laflamme L, Gallo JM, Gallo-Payet N. Activation of the AT2 receptor of angiotensin II induces neurite outgrowth and cell migration in microexplant cultures of the cerebellum. J Biol Chem 1999;274:31686–31692. 10531378 10.1074/jbc.274.44.31686

[230] Bedecs K , Elbaz N, Sutren M, Masson M, Susini C, Strosberg AD, Nahmias C. Angiotensin II type 2 receptors mediate inhibition of mitogen-activated protein kinase cascade and functional activation of SHP-1 tyrosine phosphatase. Biochem J 1997;325:449–454. 9230127 10.1042/bj3250449 PMC1218581

[231] Huang X -C, Richards EM, Sumners C . Angiotensin II type 2 receptor-mediated stimulation of protein phosphatase 2A in rat hypothalamic/brainstem neuronal cocultures. J Neurochem 1995;65:2131–2137. 7595499 10.1046/j.1471-4159.1995.65052131.x

[232] Zhao Y , Foryst-Ludwig A, Bruemmer D, Culman J, Bader M, Unger T, Kintscher U. Angiotensin II induces peroxisome proliferator-activated receptor gamma in PC12W cells via angiotensin type 2 receptor activation. J Neurochem 2005;94:1395–1401. 15992368 10.1111/j.1471-4159.2005.03275.x

[233] Rodriguez-Pallares J , Rey P, Parga JA, Muñoz A, Guerra MJ, Labandeira-Garcia JL. Brain angiotensin enhances dopaminergic cell death via microglial activation and NADPH-derived ROS. Neurobiol Dis 2008;31:58–73. 18499466 10.1016/j.nbd.2008.03.003

[234] Bhat SA , Sood A, Shukla R, Hanif K. AT2R activation prevents microglia pro-inflammatory activation in a NOX-dependent manner: inhibition of PKC activation and p47 phox phosphorylation by PP2A. Mol Neurobiol 2019;56:3005–3023. 30076526 10.1007/s12035-018-1272-9

[235] Wosik K , Cayrol R, Dodelet-Devillers A, Berthelet F, Bernard M, Moumdjian R, Bouthillier A, Reudelhuber TL, Prat A. Angiotensin II controls occludin function and is required for blood-brain barrier maintenance: relevance to multiple sclerosis. J Neurosci 2007;27:9032–9042. 17715340 10.1523/JNEUROSCI.2088-07.2007 PMC6672193

[236] Xue B , Zhang Y, Johnson AK. Interactions of the brain renin-angiotensin-system (RAS) and inflammation in the sensitization of hypertension. Front Neurosci 2020;14:650. 32760236 10.3389/fnins.2020.00650 PMC7373760

[237] Huber G , Schuster F, Raasch W. Brain renin-angiotensin system in the pathophysiology of cardiovascular diseases. Pharmacol Res 2017;125:72–90. 28687340 10.1016/j.phrs.2017.06.016

[238] Zucker IH , Patel KP, Schultz HD. Neurohumoral Stimulation. Heart Fail Clin 2012;8:87–99. 22108729 10.1016/j.hfc.2011.08.007 PMC3224981

[239] Von Bohlen Und Halbach O , Albrecht D. The CNS renin-angiotensin system. Cell Tissue Res 2006;326:599–616. 16555051 10.1007/s00441-006-0190-8

[240] Medelsohn FAO , Jenkins TA, Berkovic SF. Effects of angiotensin II on dopamine and serotonin turnover in the striatum of conscious rats. Brain Res 1993;613:221–229. 7514480 10.1016/0006-8993(93)90902-y

[241] Brown DC , Steward LJ, Ge J, Barnes NM. Ability of angiotensin II to modulate striatal dopamine release via the AT1 receptor in vitro and in vivo. Br J Pharmacol 1996;118:414–420. 8735646 10.1111/j.1476-5381.1996.tb15418.x PMC1909619

[242] Szekeres M , Nádasy GL, Turu G, Süpeki K, Szidonya L, Buday L, Chaplin T, Clark AJ, Hunyady L. Angiotensin II-induced expression of brain-derived neurotrophic factor in human and rat adrenocortical cells. Endocrinology 2010;151:1695–1703. 20181798 10.1210/en.2009-1060

[243] Namsolleck P , Boato F, Schwengel K, Paulis L, Matho KS, Geurts N, Thöne-Reineke C, Lucht K, Seidel K, Hallberg A, Dahlöf B, Unger T, Hendrix S, Steckelings UM. AT2-receptor stimulation enhances axonal plasticity after spinal cord injury by upregulating BDNF expression. Neurobiol Dis 2013;51:177–191. 23174180 10.1016/j.nbd.2012.11.008

[244] Wright JW , Harding JW. Brain renin-angiotensin-a new look at an old system. Progr Neurobiol 2011;95:49–67. 10.1016/j.pneurobio.2011.07.001 21777652

[245] Pariante CM , Lightman SL. The HPA axis in major depression: classical theories and new developments. Trends Neurosci 2008;31:464–468. 18675469 10.1016/j.tins.2008.06.006

[246] Watson S , Gallagher P, Ritchie JC, Ferrier IN, Young AH. Hypothalamic-pituitary-adrenal axis function in patients with bipolar disorder. Br J Psychiatry 2004;184:496–502. 15172943 10.1192/bjp.184.6.496

[247] Balthazar L , Lages YVM, Romano VC, Landeira-Fernandez J, Krahe TE. The association between the renin-angiotensin system and the hypothalamic-pituitary-adrenal axis in anxiety disorders: A systematic review of animal studies. Psychoneuroendocrinology 2021;132:105354. 34329905 10.1016/j.psyneuen.2021.105354

[248] Gadelha A , Vendramini AM, Yonamine CM, Nering M, Berberian A, Suiama MA, Oliveira V, Lima-Landman MT, Breen G, Bressan RA, Abílio V, Hayashi MA. Convergent evidences from human and animal studies implicate angiotensin I-converting enzyme activity in cognitive performance in schizophrenia. Transl Psychiatry 2015;5:e691. 26645626 10.1038/tp.2015.181 PMC5068582

[249] Oh SJJ , Fan X. The possible role of the angiotensin system in the pathophysiology of schizophrenia: implications for pharmacotherapy. CNS Drugs 2019;33:539–547. 30993607 10.1007/s40263-019-00632-4

[250] Martin S , Markus MA, Morris BJ, Davisson RL, Lawrence AJ, van den Buuse M. Does angiotensin interact with dopaminergic mechanisms in the brain to modulate prepulse inhibition in mice? Neuropharmacology 2008;54:399–404. 18037452 10.1016/j.neuropharm.2007.10.008

[251] Fujita T , Hirooka K, Nakamura T, Itano T, Nishiyama A, Nagai Y, Shiraga F. Neuroprotective effects of angiotensin II type 1 receptor (AT1-R) blocker via modulating AT1-R signaling and decreased extracellular glutamate levels. Invest Ophthalmol Vis Sci 2012;53:4099–4110. 22661470 10.1167/iovs.11-9167

[252] Kobiec T , Otero-Losada M, Chevalier G, Udovin L, Bordet S, Menéndez-Maissonave C, Capani F, Pérez-Lloret S. The renin–angiotensin system modulates dopaminergic neurotransmission: a new player on the scene. Front Synaptic Neurosci 2021;13:638519. 33967734 10.3389/fnsyn.2021.638519 PMC8100578

[253] Skidgel RA , Engelbrecht S, Johnson AR, Erdös EG. Hydrolysis of substance P and neurotensin by converting enzyme and neutral endopeptidase. Peptides 1984;5:769–776. 6208535 10.1016/0196-9781(84)90020-2

[254] Binder EB , Kinkead B, Owens MJ, Nemeroff CB. The role of neurotensin in the pathophysiology of schizophrenia and the mechanism of action of antipsychotic drugs. Biol Psychiatry 2001;50:856–872. 11743941 10.1016/s0006-3223(01)01211-2

[255] Sharma RP , Janicak PG, Bissette G, Nemeroff CB. CSF neurotensin concentrations and antipsychotic treatment in schizophrenia and schizoaffective disorder. Am J Psychiatry 1997;154:1019–1021. 9210757 10.1176/ajp.154.7.1019

[256] Atlas SA . The renin-angiotensin aldosterone system: Pathophysiological role and pharmacologic inhibition. J Manag Care Pharm 2007;13:9–20. 17970613 10.18553/jmcp.2007.13.s8-b.9 PMC10437584

[257] Mentz RJ , Bakris GL, Waeber B, McMurray JJ, Gheorghiade M, Ruilope LM, Maggioni AP, Swedberg K, Piña IL, Fiuzat M, O'Connor CM, Zannad F, Pitt B. The past, present and future of renin-angiotensin aldosterone system inhibition. Int J Cardiol 2013;167:1677–1687. 23121914 10.1016/j.ijcard.2012.10.007 PMC4145865

[258] Luo H , Wu PF, Cao Y, Jin M, Shen TT, Wang J, Huang JG, Han QQ, He JG, Deng SL, Ni L, Hu ZL, Long LH, Wang F, Chen JG. Angiotensin-converting enzyme inhibitor rapidly ameliorates depressive-type behaviors via bradykinin-dependent activation of mammalian target of rapamycin complex 1. Biol Psychiatry 2020;88:415–425. 32220499 10.1016/j.biopsych.2020.02.005

[259] Saavedra JM , Armando I, Bregonzio C, Juorio A, Macova M, Pavel J, Sanchez-Lemus E. A centrally acting, anxiolytic angiotensin II AT1 receptor antagonist prevents the isolation stress-induced decrease in cortical CRF 1 receptor and benzodiazepine binding. Neuropsychopharmacology 2006;31:1123–1134. 16205776 10.1038/sj.npp.1300921

[260] Srinivasan J , Suresh B, Ramanathan M. Differential anxiolytic effect of enalapril and losartan in normotensive and renal hypertensive rats. Physiol Behav 2003;78:585–591. 12782212 10.1016/s0031-9384(03)00036-2

[261] Costall B , Domeney AM, Gerrard PA, Horovitz ZP, Kelly ME, Naylor RJ, Tomkins DM. Effects of captopril and SQ29,852 on anxiety-related behaviours in rodent and marmoset. Pharmacol Biochem Behav 1990;36:13–20. 2112256 10.1016/0091-3057(90)90118-2

[262] Ayyub M , Najmi AK, Akhtar M. Protective effect of irbesartan an angiotensin (AT1) receptor antagonist in unpredictable chronic mild stress induced depression in mice. Drug Res (Stuttg) 2017;67:59–64. 27756096 10.1055/s-0042-118172

[263] Ping G , Qian W, Song G, Zhaochun S. Valsartan reverses depressive/anxiety-like behavior and induces hippocampal neurogenesis and expression of BDNF protein in unpredictable chronic mild stress mice. Pharmacol Biochem Behav 2014;124:5–12. 24844704 10.1016/j.pbb.2014.05.006

[264] Aswar U , Chepurwar S, Shintre S, Aswar M. Telmisartan attenuates diabetes induced depression in rats. Pharmacol Rep 2017;69:358–364. 28189098 10.1016/j.pharep.2016.12.004

[265] Zakrocka I , Targowska-Duda KM, Wnorowski A, Kocki T, Jóźwiak K, Turski WA. Angiotensin II type 1 receptor blockers inhibit KAT II activity in the brain—its possible clinical applications. Neurotox Res 2017;32:639–648. 28733707 10.1007/s12640-017-9781-2 PMC5602025

[266] Büki A , Kekesi G, Horvath G, Vécsei L. A potential interface between the kynurenine pathway and autonomic imbalance in schizophrenia. Int J Mol Sci 2021;22:10016. 34576179 10.3390/ijms221810016 PMC8467675

[267] Kakuta H , Kurosaki E, Niimi T, Gato K, Kawasaki Y, Suwa A, Honbou K, Yamaguchi T, Okumura H, Sanagi M, Tomura Y, Orita M, Yonemoto T, Masuzaki H. Distinct properties of telmisartan on agonistic activities for peroxisome proliferator-activated receptor γ among clinically used angiotensin II receptor blockers: drug-target interaction analyses. J Pharmacol Exp Ther 2014;349:10–20. 24424487 10.1124/jpet.113.211722

[268] Vasconcelos GS , dos Santos Júnior MA, Monte AS, Monte AS, da Silva FER, Lima CNC, Moreira Lima Neto AB, Medeiros IDS, Teixeira AL, de Lucena DF, Vasconcelos SMM, Macedo DS. Low-dose candesartan prevents schizophrenia-like behavioral alterations in a neurodevelopmental two-hit model of schizophrenia. Prog Neuropsychopharmacol Biol Psychiatry 2021;111:110348. 33984421 10.1016/j.pnpbp.2021.110348

[269] Chen S , Ge Y, Si J, Rifai A, Dworkin LD, Gong R. Candesartan suppresses chronic renal inflammation by a novel antioxidant action independent of AT1R blockade. Kidney Int 2008;74:1128–1138. 18650791 10.1038/ki.2008.380

[270] Ancelin ML , Carrière I, Scali J, Ritchie K, Chaudieu I, Ryan J. Angiotensin-converting enzyme gene variants are associated with both cortisol secretion and late-life depression. Transl Psychiatry 2013;3:e322. 24193727 10.1038/tp.2013.95 PMC3849962

[271] Firouzabadi N , Shafiei M, Bahramali E, Ebrahimi SA, Bakhshandeh H, Tajik N. Association of angiotensin-converting enzyme (ACE) gene polymorphism with elevated serum ACE activity and major depression in an Iranian population. Psychiatry Res 2012;200:336–342. 22688325 10.1016/j.psychres.2012.05.002

[272] Zill P , Baghai TC, Schüle C, Born C, Früstück C, Büttner A, Eisenmenger W, Varallo-Bedarida G, Rupprecht R, Möller HJ, Bondy B. DNA methylation analysis of the angiotensin converting enzyme (ACE) gene in major depression. PLoS ONE 2012;7:e40479. 22808171 10.1371/journal.pone.0040479 PMC3396656

[273] Baghai TC , Binder EB, Schule C, Salyakina D, Eser D, Lucae S, Zwanzger P, Haberger C, Zill P, Ising M, Deiml T, Uhr M, Illig T, Wichmann HE, Modell S, Nothdurfter C, Holsboer F, Müller-Myhsok B, Möller HJ, Rupprecht R, Bondy B. Polymorphisms in the angiotensin-converting enzyme gene are associated with unipolar depression, ACE activity and hypercortisolism. Mol Psychiatry 2006;11:1003–1015. 16924268 10.1038/sj.mp.4001884

[274] Annerbrink K , Jönsson EG, Olsson M, Nilsson S, Sedvall GC, Anckarsäter H, Eriksson E. Associations between the angiotensin-converting enzyme insertion/deletion polymorphism and monoamine metabolite concentrations in cerebrospinal fluid. Psychiatry Res 2010;179:231–234. 20483169 10.1016/j.psychres.2009.04.018

[275] Mazaheri H , Saadat M. Association between insertion/deletion polymorphism in angio-tension converting enzyme and susceptibility to schizophrenia. Iran J Public Health 2015;44:369–373. 25905080 PMC4402415

[276] Song GG , Lee YH. The insertion/deletion polymorphism in the angiotensin-converting enzyme and susceptibility to schizophrenia or Parkinson’s disease: a meta-analysis. JRAAS - J Renin Angiotensin Aldosterone Syst 2015;16:434–442. 25143327 10.1177/1470320313495909

[277] Mohite S , de Campos-Carli SM, Rocha NP, Sharma S, Miranda AS, Barbosa IG, Salgado JV, Simoes-E-Silva AC, Teixeira AL. Lower circulating levels of angiotensin-converting enzyme (ACE) in patients with schizophrenia. Schizophr Res 2018;202:50–54. 29925475 10.1016/j.schres.2018.06.023

[278] Consortium TSWG of the PG, Ripke S, Walters JT, O’Donovan MC. Mapping genomic loci prioritises genes and implicates synaptic biology in schizophrenia. medRxiv 2020;2020.09.12.20192922.

[279] Chauquet S , Zhu Z, O’Donovan MC, Walters JTR, Wray NR, Shah S. Association of antihypertensive drug target genes with psychiatric disorders: a Mendelian randomization study. JAMA Psychiatry 2021;78:623–631. 33688928 10.1001/jamapsychiatry.2021.0005 PMC7948097

[280] Holman EA . Acute stress and cardiovascular health: is there an ACE gene connection? J Trauma Stress 2012;25:592–597. 23055331 10.1002/jts.21746

[281] Kehoe AD , Eleftheriou KI, Heron M, Coats TJ, Montgomery HE. Angiotensin-converting enzyme genotype may predict survival following major trauma. Emerg Med J 2008;25:759–761. 18955615 10.1136/emj.2006.045336

[282] Erhardt A , Lucae S, Kern N, Unschuld PG, Ising M, Lieb R, Uhr M, Hohoff C, Deckert J, Bandelow B, Maier W, Binder EB, Müller-Myhsok B, Keck ME, Holsboer F. Association of polymorphisms in the angiotensin-converting enzyme gene with syndromal panic attacks. Mol Psychiatry 2008;13:242–243. 18285758 10.1038/sj.mp.4002094

[283] Nylocks KM , Michopoulos V, Rothbaum AO, Almli L, Gillespie CF, Wingo A, Schwartz AC, Habib L, Gamwell KL, Marvar PJ, Bradley B, Ressler KJ. An angiotensin-converting enzyme (ACE) polymorphism may mitigate the effects of angiotensin-pathway medications on posttraumatic stress symptoms. Am J Med Genet B Neuropsychiatr Genet 2015;168:307–315. 10.1002/ajmg.b.32313 25921615

[284] Lehmann DJ , Cortina-Borja M, Warden DR, Smith AD, Sleegers K, Prince JA, van Duijn CM, Kehoe PG. Large meta-analysis establishes the ACE insertion-deletion polymorphism as a marker of Alzheimer’s disease. Ame J Epidemiol 2005;162:305–317. 10.1093/aje/kwi202 16033878

[285] Rigat B , Hubert C, Alhenc-Gelas F, Cambien F, Corvol P, Soubrier F. An insertion/deletion polymorphism in the angiotensin I-converting enzyme gene accounting for half the variance of serum enzyme levels. J Clin Invest 1990;86:1343–1346. 1976655 10.1172/JCI114844 PMC296868

[286] Kehoe PG , Russ C, McIlroy S, Holmans P, Holmes C, Liolitsa D, Vahidassr D, Powell J, McGleenon B, Liddell M, Plomin R, Dynan K, Williams N, Neal J, Cairns NJ, Wilcock G, Passmore P, Lovestone S, Williams J, Owen MJ. Variation in DCP1, encoding ACE, is associated with susceptibility to Alzheimer disease. Nat Gen 1999;21:71–72. 10.1038/5009 9916793

[287] Chou PS , Wu MN, Chou MC, Chien I, Yang YH. Angiotensin-converting enzyme insertion/deletion polymorphism and the longitudinal progression of Alzheimer’s disease. Geriatr Gerontol Int 2017;17:1544–1550. 27862810 10.1111/ggi.12929

[288] Oliveira FF de, de Almeida SS, Smith MC, Bertolucci PHF . Behavioural effects of the ACE insertion/deletion polymorphism in Alzheimer’s disease depend upon stratification according to APOE-ɛ4 carrier status. Cogn Neuropsychiatry 2021;26:293–305. 34034613 10.1080/13546805.2021.1931085

[289] Reyes M , Stanton PK. Induction of hippocampal long-term depression requires release of CA2 + from separate presynaptic and postsynaptic intracellular stores. J Neurosci 1996;16:5951–5960. 8815877 10.1523/JNEUROSCI.16-19-05951.1996 PMC6579182

[290] Egorova PA , Bezprozvanny IB. Inositol 1,4,5-trisphosphate receptors and neurodegenerative disorders. FEBS J 2018;285:3547–3565. 29253316 10.1111/febs.14366

[291] Kovacs G , Reimer L, Jensen PH. Endoplasmic reticulum-based calcium dysfunctions in synucleinopathies. Front Neurol 2021;12:742625. 34744980 10.3389/fneur.2021.742625 PMC8563702

[292] Liss BR . Molecular physiology of neuronal K-ATP channels. Mol Membr Biol 2001;18:117–127. 11463204

[293] Chen Y-F , Chen L-H, Yeh Y-M, Wu P-Y, Chen Y-F, Chang L-Y, Chang J-Y, Shen M-R. Minoxidil is a potential neuroprotective drug for paclitaxel-induced peripheral neuropathy. Sci Rep 2017;7:45366. 28349969 10.1038/srep45366 PMC5368986

[294] Takatani T , Takahashi K, Jin C, Matsuda T, Cheng X, Ito T, Azuma J. Minoxidil attenuates ischemia-induced apoptosis in cultured neonatal rat cardiomyocytes. J Cardiovas Pharmacol 2004;43:789–794. 10.1097/00005344-200406000-00008 15167272

[295] Blasco-Serra A , Escrihuela-Vidal F, González-Soler EM, Martínez-Expósito F, Blasco-Ausina MC, Martínez-Bellver S, Cervera-Ferri A, Teruel-Martí V, Valverde-Navarro AA. Depressive-like symptoms in a reserpine-induced model of fibromyalgia in rats. Physiol Behav 2015;151:456–462. 26222614 10.1016/j.physbeh.2015.07.033

[296] Leão AHFF , Sarmento-Silva AJ, Santos JR, Ribeiro AM, Silva RH. Molecular, neurochemical, and behavioral hallmarks of reserpine as a model for Parkinson’s disease: new perspectives to a long-standing model. Brain Pathol 2015;25:377–390. 25726735 10.1111/bpa.12253 PMC8029054

[297] Walker VM , Kehoe PG, Martin RM, Davies NM. Repurposing antihypertensive drugs for the prevention of Alzheimer’s disease: a Mendelian randomization study. Int J Epidemiol 2020;49:1132–1140. 31335937 10.1093/ije/dyz155 PMC7751008

[298] Gill D , Georgakis MK, Koskeridis F, Jiang L, Feng Q, Wei WQ, Theodoratou E, Elliott P, Denny JC, Malik R, Evangelou E, Dehghan A, Dichgans M, Tzoulaki I. Use of genetic variants related to antihypertensive drugs to inform on efficacy and side effects. Circulation 2019;140:270–279. 31234639 10.1161/CIRCULATIONAHA.118.038814 PMC6687408

[299] Ou Y-N , Yang Y-X, Shen X-N, Ma Y-H, Chen S-D, Dong Q, Tan L, Yu J-T. Genetically determined blood pressure, antihypertensive medications, and risk of Alzheimer's disease: a Mendelian randomization study. Alzheimers Res Ther 2021;13:41. 33563324 10.1186/s13195-021-00782-y PMC7874453

