# Erratum to: Antihypertensive drugs and brain function: mechanisms underlying therapeutically beneficial and harmful neuropsychiatric effects

**DOI:** 10.1093/cvr/cvad055

**Published:** 2023-04-11

This is an erratum to: Carla Carnovale, Cristiana Perrotta, Sara Baldelli, Dario Cattaneo, Cristina Montrasio, Silvia S Barbieri, Giulio Pompilio, Chiara Vantaggiato, Emilio Clementi, Marco Pozzi, Antihypertensive drugs and brain function: mechanisms underlying therapeutically beneficial and harmful neuropsychiatric effects, *Cardiovascular Research*, 2022, cvac110, https://doi.org/10.1093/cvr/cvac110

In the originally published online version of this manuscript Table 2 was published as plain text without colour markers. This information was correct in the PDF version and so only the online version has been updated. The publisher apologizes to the authors and readers for any confusion caused.

This error in the online version has now been corrected.

