# Supplementary material for: Antihypertensive drugs and brain function: mechanisms underlying therapeutically beneficial and harmful neuropsychiatric effects
Source: Cardiovasc Res. 2022 Jul 28;119(3):647–67. doi: 10.1093/cvr/cvac110 (PMC10153433; doi:10.1093/cvr/cvac110)
Supplement: cvac110_Supplementary_Data [file cvac110_supplementary_data.docx]

**STable 1. The main characteristics of studies reporting beneficial vs harmful effects of each antihypertensive drug class in the neuropsychiatric clinic, published in any clinical setting over the last 20 years.**

| **Author** | **Study design** | **Arms** | **Patients (n)** | **Cardiovascular Diagnosis** | **Psychiatric Diagnosis** | **Males (%)** | **Age** | **Concomitant therapy** | **OUTCOME (Assessment)** | **MAIN FINDINGS** |
| --- | --- | --- | --- | --- | --- | --- | --- | --- | --- | --- |
|  |  |  |  |  |  |  | **(Mean±SD)** |  |  |  |
| ***More than one Antihypertensive drug class*** | | | | | | | | | | |
|  |  |  |  |  |  |  |  |  |  |  |
| ***Béné***  ***2012*** | Prospective observational studies | ACEIs ARBs Diuretics  β-blockers CCBs | 179 | Stroke | None | 58.7 | 60.8±14.8 | None | **Post-stroke cognitive decline (MMSE ≤ 24)** | ACEIs increased significantly post-stroke cognitive decline (OR: 4.07; 95%CI: 1.01-16.43), β-blockers showed a trend to cognitive protection(P:0.12), CCBs was associated to cognitive decline (P:0.10) |
|  |  |  |  |  |  |  |  |  |  |  |
| ***Boal***  ***2016*** | Retrospective cohort study | ACEIs  ARBs | 10814 | Hypertension | MDD, BD | 56 | 60.1±11.1 | NA | **Risk for mood disorder** | Patients on ACEIs or ARBs had the lowest risk for mood disorder admissions |
|  |  | β-blockers | 11605 |  |  | 45,1 | 56.9±11.2 |  | **Risk for mood disorder** | Compared with the 1° group, β-blockers showed higher risk (hazard ratio=2.11; [95% confidence interval, 1.12–3.98]; P=0.02) |
|  |  | CCBs | 5880 |  |  | 48,5 | 62.9±9.9 |  | **Risk for mood disorder** | Compared with the 1° group, CCBs showed higher risk (2.28 [95% confidence interval, 1.13–4.58]; P=0.02) |
|  |  | Diuretics | 3831 |  |  | 28,5 | 63.5±10.2 |  | **Risk for mood disorder** | Compared with the 1° group, thiazide diuretics showed no significant difference (1.56 [95% confidence interval, 0.65–3.73]; P=0.32) |
|  |  | Control | 111936 |  |  | 48.5 | 54.3±10.9 |  | **Risk for mood disorder** | Compared with the 1° group, control group showed no significant difference (1.63 [95% confidence interval, 0.94–2.82]; P=0.08) |
| ***Callréus***  ***2007*** | Case-Control study | ACEIs ARBs Diuretics  β-blockers CCBs | 743 | None | Depression or suicide risk | 68.2 | Males: 53.4 (±18.6) | Any type | **Suicide risk**  (Danish Registry of Cause of Death, and the Odense University Pharmacoepidemiological Database) | Suicide risk was associated with current ARBs (OR: 3.52; 95% CI: 1.33–9.30) |
|  |  |  |  |  |  |  | Females: 58.1 (±17.6) |  |  |  |
| ***Climent***  ***2013*** | Prospective observational studies | ACEIs ARBs Diuretics | 618 | Hypertension | NA | NA | 74.41±6.4 | None | **Cognitive impairment (**SPMSQ ≥3 errors; MMSE ≤24) | Better SPMSQ and MMSE score for elderly hypertensive people in treatment with CCBs [(OR) 1.95 CI (1.10–3.33); P = 0.02]. |
|  |  | β-blockers CCBs |  |  |  |  |  |  |  |  |
| ***Colbourne 2021*** | Cohort Study | CCBs+diuretics | 192,161 | Hypertension | None | 52 | Diuretics: 59.9±14.6 | None | **Incidence rate of major psychiatric disorders** | CCBs were associated with a lower incidence of psychotic, affective, and anxiety disorders than β-blockers (risk ratios 0.69–0.99) and a higher incidence than ARBs (risk ratios 1.04–2.23) for both first and recurrent diagnoses.  All drug classes were also associated with the incidence of substance use and sleep disorders. |
|  |  |  |  |  |  |  | CCB: 59.9±14.7 |  |  |  |
|  |  | CCBs+ACEIs | 270,326 |  |  | 48 | ACEI: 60.4±13.8 | None | **Incidence rate of major psychiatric disorders** |  |
|  |  |  |  |  |  |  | CCB: 60.5±14.4 |  |  |  |
|  |  | CCB+ARBs | 192,164 |  |  | CCB:47 | ARB: 60.3±12.8 | None | **Incidence rate of major psychiatric disorders** |  |
|  |  |  |  |  |  | ARB:46 | CCB: 59.6± 14.3 |  |  |  |
|  |  | CCB+ β-blockers | 260,703 |  |  | 48 | 59.8±14.4 | None | **Incidence rate of major psychiatric disorders** |  |
| ***Hu***  ***2021*** | Retrospective case-matched cohort study | ACEIs | 13,974 | Hypertension, stroke, coronary artery disease, heart failure | None | 60.3 | 58.0±13.8 | α-blockers, β-blockers, potassium sparing diuretics, thiazides, loop diuretics, CCB, others | **Incidence rate of major psychiatric disorders** | 41.0 per 1,000 person-years in ACEI cohort. |
|  |  |  |  |  |  |  |  |  |  | ACEI users had a higher risk of major psychiatric disorders than ARB users, (adj HR = 1.07; 95% CI = 1.02, 1.13) |
|  |  | ARBs | 13,974 |  |  | 60.1 | 58.0±14.0 | α-blockers, β-blockers, potassium sparing diuretics, thiazides, loop diuretics, CCB, others | **Incidence rate of major psychiatric disorders** | 39.9 per 1,000 person-years in ARB cohort. |
|  |  |  |  |  |  |  |  |  |  | ACEI users had a higher risk of major psychiatric disorders than ARB users, (adj HR = 1.07; 95% CI = 1.02, 1.13) |
| ***Kessing***  ***2018*** | Retrospective cohort study | ACE-I, ARBs | 494,183 | Hypertension, congestive heart failure | Depressive disorder | 51.2 | 61 (52;71)* | NA | **Incidence rate of depression** | Data Sources: Danish nationwide population-based registers (ICD codes: DF32-DF33.31). Continued use of angiotensin agents was associated with a decreased rate of incident depression |
| ***Korosi***  ***2017*** | Cross-sectional and prospective study | ACE-I, ARBs, CCB, Beta-Blockers, alpha-1-Blockers, diuretics | 22 | Hypertension | None | 36 | 31 (26;41.2)* | Statins, acetylsalicylic acid | **Psychometric parameters**  **(**DI; HAM-A; SCL-90; TEMPS-A; BFI-44; BEQ; PVAQ) | Significant improvements in BDI (0.73 points) and in several Scl-90 subscales |
|  |  | Control | 31 |  |  | 71 | 47 (38;63)* | None | **Psychometric parameters (**DI; HAM-A; SCL-90; TEMPS-A; BFI-44; BEQ; PVAQ) | BDNF was not different between groups |
|  |  |  |  |  |  | ***ACEIs*** | | | | |
| ***Braszko***  ***2003*** | Clinical trial | Captopril | 15 | Hypertension | None | 47 | 54.67±2.03 | None | **Cognition function and depressed mood** (RAVLT; Wechsler Memory Scale; BDI; HSC) | Treatment with captopril was poorer in reversing the deficits of hypertension-induced cognition, through RAVLT; Wechsler Memory Scale; BDI; HSC |
|  |  | Enalapril | 15 |  |  | 47 | 52.07±1.39 | None | **Cognition function and depressed mood** (RAVLT; Wechsler Memory Scale; BDI; HSC) | Enalapril significantly reversed the impairment resulting from the pre-existing hypertension with respect to learning ability and slightly reversed the deficits in immediate and delayed recall of verbal tasks. |
|  |  | No treatment | 9 |  |  | 55.6 | 51.89±3.84 | None | **Cognition function and depressed mood** (RAVLT; Wechsler Memory Scale; BDI; HSC) | Untreated hypertensive patients can have impaired cognition, particularly learning. |
|  |  | Control | 15 | None |  | 47 | 54.57±2.03 | None | **Cognition function and depressed mood** (RAVLT; Wechsler Memory Scale; BDI; HSC) | Untreated hypertensive patients can have impaired cognition, particularly learning. |
| ***Tarlow***  ***2000*** | Case Report | Quinalapril | 1 | Congestive heart failure | None | 0 | 93 | Acetaminophen/hydrocodone; lansoprazole; acetylsalicylic acid; calcium carbonate; furosemide; potassium chloride; spironolactone; nitroglycerin; metolazone | **Safety outcome** | Visual hallucinations, paranoid delusions, confusion, disorientation, anxiety. |
| ***Williams***  ***2016*** | Nested case-control study | Perindopril, ramipril, fosinopril, enalapril, lisinopril, quinalapril, trandopril, captopril | 125 (3 MD patients; 122 controls) | None | Mood disorder | 100 | 52.0 (42.5-64.4)* | Antidepressants, hormone therapy, NSAID, other hypertensive agents, diuretics | **Risk of Mood Disorders** | ACEIs were associated with a reduced likelihood for mood disorder onset.  Among the 756 non-exposed participants, 40 (5.3%) developed de novo mood disorders |
| ***Williams***  ***2016*** | Retrospective cohort study | Perindopril, ramipril, fosinopril, enalapril, lisinopril, trandopril, captopril | 80 (756 non-exposed participants) | Hypertension | None | 100 | NA | NA | **Risk of Mood Disorders** | 0 (0%) patients developed de novo mood disorders |
|  |  |  |  |  | ***Alpha-2 Agonists*** | | | | | |
| ***Akouchakian***  ***2021*** | RCT | Clonidine | 28 | None | OCD | 67.9 | 36.57±9.78 | SSRI or clomipramine | **OCD symptoms** (Y-BOCS, CGI-S) | CGI: 3.89±1.57 vs 2.29±1.18; YBOCS: 27.61±8.08 vs 20.25±6.80 |
|  |  |  |  |  |  |  |  |  |  | Although the use of clonidine posed no remarkable drug-related adverse effects, it was not superior to placebo considering symptom relief. |
|  |  | Placebo | 29 |  |  | 62.1 | 36±10.23 | SSRI or clomipramine | **OCD symptoms** (Y-BOCS, CGI-S) | CGI: 4.10±1.61 vs 3.07±1.51; YBOCS: 28.69±7.44 vs 25.45±7.35 |
| ***Aksu***  ***2019*** | Case Report | Clonidine | 1 | None | Schizophraenia | 0 | 42 | None | **Schizophraenia symptoms** (PSYRATS, PANSS, CDSS, HAM-A) | After 7 days, dramatic reduction of the auditory hallucinations and reduction in the emotional component of the delusions was observed. After 6-months follow-up, auditory hallucinations had resolved almost completely. PSYRATS: 16/9, PANSS: 12/17/29, CDSS: 10, HAM-A: 16 |
| ***Biederman 2008 (A;B)*** | RCT | Placebo | 86 | None | ADHD | 74.4 | 10.6 | None | **ADHD symptoms (**ADHD, CGI-I, PGA, CPRS-R, CTRS-R) | The mean reduction in ADHD-RS-IV scores at the endpoint across children taking placebo was -8.9. Significant improvement in CGI-I scores at the endpoint was shown in 25.64% in the placebo group. |
|  |  |  |  |  |  |  |  |  |  | Significant improvement in PGA scores at the endpoint was shown in 23.08% in the placebo group. |
|  |  | Guanfacine 2 mg | 87 |  |  | 77 | 10.6 | None | **ADHD symptoms** (ADHD, CGI-I, PGA, CPRS-R, CTRS-R) | The mean reduction in ADHD-RS-IV scores at the endpoint across all groups of children taking GXR was -16.7. Significant improvement in CGI-I scores at the endpoint was shown in 55.95%. Significant improvement in PGA scores at the endpoint was shown in 62.12%. All groups of children taking GXR showed significant improvement from baseline in CPRS-R and CTRS-R mean day total scores, compared with placebo. |
|  |  | Guanfacine 3 mg | 86 |  |  | 80.2 | 10.8 | None | **ADHD symptoms (**ADHD, CGI-I, PGA, CPRS-R, CTRS-R) | The mean reduction in ADHD-RS-IV scores at the endpoint across all groups of children taking GXR was -16.7. Significant improvement in CGI-I scores at the endpoint was shown in 50%. |
|  |  |  |  |  |  |  |  |  |  | Significant improvement in PGA scores at the endpoint was shown in 50.82%. All groups of children taking GXR showed significant improvement from baseline in CPRS-R and CTRS-R mean day total scores, compared with placebo. |
|  |  | Guanfacine 4 mg | 86 |  |  | 66.3 | 10.1 | None | **ADHD symptoms** (ADHD, CGI-I, PGA, CPRS-R, CTRS-R) | The mean reduction in ADHD-RS-IV scores at the endpoint across all groups of children taking GXR was -16.7. |
|  |  |  |  |  |  |  |  |  |  | Significant improvement in CGI-I scores at the endpoint was shown in 55.56%. |
|  |  |  |  |  |  |  |  |  |  | Significant improvement in PGA scores at the endpoint was shown in 66.10%. |
|  | OLE | Guanfacine (OLE) | 240 |  |  | 76.7 | 10.5±2.6 | None | **ADHD symptoms (**ADHD, CGI-I, PGA, CPRS-R, CTRS-R) | The most common adverse events were somnolence (30.4%), headache (26.3%), fatigue (14.2%) and sedation (13.3). |
|  |  |  |  |  |  |  |  |  |  | ADHD-RS-IV total score was significantly reduced from baseline to endpoint. Reductions were apparent at 1 month and were sustained for up to 24 months for those subjects who continued in the study for 2 years. Significant decreases in ADHD-RS-IV total scores were also achieved in the 6-12-years and a13-years age groups. PGA: 58.6% of subjects were improved at the endpoint. CHQ-PF50 scores did not change significantly from baseline to endpoint overall or in any dose or age group. |
| ***Bunevicius 2005*** | Cross-Over Trial | Clonidine | 14 | None | PMDD | 0 | 34 | None | **PMDD symptoms (PRISM)** | No significant differences between clonidine and placebo for mood scales or premenstrual symptom rating |
|  |  | Placebo |  |  |  |  |  |  | **PMDD symptoms (PRISM)** |  |
| ***Butterfield 2016*** | RCT | Guanfacine | 26 (Total) | None | ADHD | 46.2 | 37.54±12.22 | Treatment approved for ADHD | **ADHD symptoms (ADHD –RS, CGI—S)** | In both groups: statistically significant improvement in their symptoms and functioning over the course of the trial; no difference in terms of their efficacy, safety, or tolerability.. Adverse events included: fatigue (GH: 30.8%, placebo 61.5%) dry mouth (GH: 38.5%, placebo 23.1%), irritability (GH: 15.4%, placebo 23.1%), headache (GH: 15.4%, placebo 23.1%), and increased appetite (GH: 7.7%, placebo 15.4%). |
|  |  | Placebo |  |  |  | (Total) | (Total) |  | **ADHD symptoms (ADHD –RS, CGI—S)** |  |
| ***Capone***  ***2016*** | Clinical trial | Guanfacine | 23 | None | Down Syndrome and Comorbid ADHD With Disruptive Behaviors | 78.26 | 7.4±4.1 | Several drugs | **ADHD symptoms (ABC, ASQ-P)** | The mean decline on Hyperactivity was 25% (–7.8 points), and for Irritability, 25% (–3.5 points). The total maladaptive behavior composite score was significantly reduced by –12 points. |
|  |  |  |  |  |  |  |  |  |  | Guanfacine improves hyperactivity and irritability in children with Down Syndrome and ADHD |
| ***Connor***  ***2013*** | Clinical trial | Guanfacine | 19 | None | PTSD, ADHD, GAD, depression. Anxiety disorder | 52.6 | 11.9±1.5 | None | **PTSD symptoms** (UCLA-RI, GAD subscale, CIS, ADHD-RS-IV, CGI Severity and Improvement Scales) | GXR may be effective in decreasing PTSD symptoms including cluster B (reexperiencing), cluster C (avoidant), and cluster D (overarousal) symptoms |
| ***Connor***  ***2000*** | RCT | Methylphenidate | 8 | None | ADHD/ODD/CD | 100 | 8.9±2.6 | None | **ADHD symptoms** (DBS, APRS, HSQ SSQ, GGDS, GPB, Side-Effects Rating Scale) | Clonidine group experienced significantly greater impairment in fine motor task performance than the groups receiving MPH did.  There was also a marginally significant effect for the group receiving clonidine only to have slower times to complete the pegboard task with the dominant hand (F6,63 2.22, p=0.052). |
|  |  | Clonidine | 8 |  |  |  | 9.3±1.7 | None | **ADHD symptoms** (DBS, APRS, HSQ SSQ, GGDS, GPB, Side-Effects Rating Scale) |  |
|  |  | Methylphenidate+Clonidine | 8 |  |  |  | 10.1±2.4 | None | **ADHD symptoms** (DBS, APRS, HSQ SSQ, GGDS, GPB, Side-Effects Rating Scale) |  |
| ***Dardennes 2010*** | Case Report | Clonidine | 1 | None | Schizophraenia | 100 | 28 | Clozapine, aripiprazole | **Schizophraenia symptoms** (AHRS) | AHRS: 13-17 (-35% to -50%). The most common treatment-emergent adverse events in participants receiving GXR + psychostimulant were headache (21.2%) and somnolence (13.6%) |
| ***Delaney***  ***2006*** | Case Report | Clonidine | 1 | Hypertension | None | 100 | 52 | None | **Safety outcome** | Hallucinations |
| ***Elbe***  ***2016*** | Case Report | Guanfacine | 1 | None | Behavioral and emotional dysregulation, chronic sleep difficulties, self-harm and suicidal ideation | 0 | 6 | Risperidone, Clonidine | **Safety outcome** | Maniac symptoms including decreased need for sleep, excessive energy, giddiness, grandiosity, pressured speech, hypersexuality, increased self-harm and aggression |
| ***Friedman 2001*** | RCT | Placebo+typicals antipsychotics | 9 | None | Schizophraenia | 67 | 51.2 | None | **Schizophrenia symptoms** (PANSS, Simpson Angus Scale, Simple Spatial Working Memory Test, CCPT-computerized, Learning Test-RAVLT, Digit Span Distraction Test, and Verbal Fluency) | Risperidone + guanfacine: improvement in spatial working memory performance, reaction time on the CPT, and Trails B performance; the mean improvement of 3.1 correct targets on the 5 s delay of the spatial working memory test was significantly greater than that for the placebo group (U:11.5, p:0.03); the mean improvement of 3 correct target locations at the 15 s delay showed a trend difference from the placebo+risperidone group (U:18, p:0.14); the mean 55 ms improvement in CPT reaction time showed a trend difference from the placebo risperidone group (U: 12, p: 0.1); improvement of 5 words on serial learning and 1.5 words on delayed recall from the RAVLT, and a mean 40 s improvement on the Trails B test. No improvement on verbal fluency or digit span distraction test performance. |
|  |  | Guanfacine+typicals antipsychotics | 8 |  |  | 88 | 53 | None | **Schizophraenia symptoms** (PANSS, Simpson Angus Scale, Simple Spatial Working Memory Test, CCPT-computerized, Learning Test-RAVLT, Digit Span Distraction Test, and Verbal Fluency) |  |
|  |  | Risperidone+placebo | 11 |  |  | 91 | 44.1 | None | **Schizophraenia symptoms** (PANSS, Simpson Angus Scale, Simple Spatial Working Memory Test, CCPT-computerized, Learning Test-RAVLT, Digit Span Distraction Test, and Verbal Fluency) |  |
| ***Gaffney***  ***2002*** | RCT | Clonidine | 12 | None | Tourette's syndrome, OCD, ADHD | 92 | 12.1±3 | None | **Tourette’s Syndrome symptoms** (YGTSS, Y-BOCS, ADHDRS, TS-CGI severity, MOVES, HAM-D) | YGTSS: -13.8; Y-BOCS: -2.8; ADHDRS: -11.2. Secondary outcomes - MOVES: -4.2b, TS-CGI severity: -1.2b, OCD-CGI severity: -0.8b (b=baseline to endpoint change, p<0.5) |
|  |  | Risperidone | 9 |  |  | 89 | 10.4±2.7 | None | **Tourette’s Syndrome symptoms** (YGTSS, Y-BOCS, ADHDRS, TS-CGI severity, MOVES, HAM-D) | YGTSS: -10.9; Y-BOCS: -6.3; ADHDRS: -9.9 Secondary outcomes - MOVES: -2.0, TS-CGI severity: -0.6, OCD-CGI severity: -0.9b (b=baseline to endpoint change, p<0.5) |
| ***Hazell***  ***2003*** | RCT | Clonidine | 38 | None | ADHD with oppositional defiant disorder or conduct disorder | 94.7 | 9.4 | Methylphenidate, dexamphetamine | **ADHD symptoms** (Conduct and hyperactive index subscales of the Connners Behavior checklist, Barkley self-report side effect checklists) | There was a greater reduction in mean Conduct and Hyperactive Index scores for the clonidine-treated group than the placebo control group: 57% of the clonidine-treated participants improved by ≥38% on the Conduct scale compared |
|  |  | Placebo | 29 |  |  | 86.2 | 10.5 | Methylphenidate, dexamphetamine | **ADHD symptoms (**Conduct and hyperactive index subscales of the Connners Behavior checklist, Barkley self-report side effect checklists) | More treated children (35%) than placebo control children (17%) met criteria for improvement on the Hyperactive Index, but the difference was not statistically significant. |
| ***Hervas***  ***2014*** | RCT | Guanfacine | 114 | None | ADHD | 66.7 | 10.9±2.77 | None | **ADHD symptoms** (ADHD-RS-IV, CGI-I, WFIRS-P) | ADHD-RS-IV total score (placebo-adjusted differences) (GXR: [8.9, p=0.001]; ATX: [3.8, p=0.05]), the difference from placebo in the percentage of patients showing improvement (1[‘very much improved’] or 2[‘much improved’]) for CGI-I (GXR: [23.7, po0.001]; ATX:[12.1, po0.05]), WFIRS-P learning and school domain (GXR: [0.22, po0.01]; ATX:[0.16, po0.05]) and WFIRS-P family domain (GXR: [0.21, po0.01]; ATX: [0.09, p=0.242]). |
|  |  | Atomexetine | 112 |  |  | 77.7 | 10.5±2.81 | None | **ADHD symptoms** (ADHD-RS-IV, CGI-I, WFIRS-P) |  |
| ***Ingrassia***  ***2005*** | Case Series | Clonidine | 6 | None | Neurodevelopmental disorders | 83 | 11.2±2.8 | Hydrocortisone and Desmopressine (1/6); sodium valproate (1/6) | **Managing of Severe sleep problems** | All children showed maintained improvements in their sleep pattern |
| ***Iwanami***  ***2020*** | RCT | Guanfacine | 100 | None | ADHD | 66 | 31.1±8.1 | None | **ADHD symptoms** (ADHD-RS-IV, CPRS-R, CGI-S, CGI-I, PGI-I, AAQoL, BRIEF-A) | Compared to placebo, ADHD-RS-IV total scores: -4.28 (-6.67 to -1.88) P Value= 0.0005 |
|  |  |  |  |  |  |  |  |  |  | ADR: somnolence (34.7%); insomnia (5.0%) |
|  |  | Placebo | 100 |  |  | 63 | 33.8±10.2 | None | **ADHD symptoms** (ADHD-RS-IV, CPRS-R, CGI-S, CGI-I, PGI-I, AAQoL, BRIEF-A) | ADR: somnolence (8.0%); insomnia (0%) |
| ***Kollins***  ***2011 (A)*** | RCT | Placebo | 95 | None | ADHD | 66 | 10.5±2.5 | Methylphenidate or Amphetamine | **ADHD symptoms (**ADHD-RS-IV, CPRS-R, CGI-S, CGI-I, PGI-I, PGA) | Clonidine ER + stimulant group vs the placebo + stimulant group: at week 5, greater improvement from baseline in ADHD-RS-IV total score (95% confidence interval: 7.83 to 1.13; P .009), ADHD-RS-IV hyperactivity and inattention subscale scores (P .014 and P .017, respectively), Conners’ Parent Rating Scale scores (P .062), CGI-S (P .021), CGI-I (P .006), and PGA (P .001) was observed in the Clonidine ER plus stimulant group versus the placebo plus stimulant group. ADR: Somnolence (20%); Irritability (5%); Insomnia (5%) |
|  |  | Clonidine | 102 |  |  | 79 | 10.4±2.5 | Methylphenidate or Amphetamine | **ADHD symptoms (**ADHD-RS-IV, CPRS-R, CGI-S, CGI-I, PGI-I, PGA) |  |
| ***Kollins***  ***2011 (B)*** | RCT | Guanfacine | 121 | None | ADHD | 66.1 | 12.6±2.83 | None | **ADHD symptoms** (RT as measured by the CRT test from the CANTAB; PSS; PDSS; ADHD-RS-IV; CGI-I) | GXR was associated with significant improvement in ADHD symptoms (6.3 [95% CI: 2.7, 9.8], p=0.001 for ADHD Rating Scale IV total scores at endpoint). |
|  |  | Placebo | 57 |  |  | 77.2 | 12.8±2.77 | None | **ADHD symptoms** (RT as measured by the CRT test from the CANTAB; PSS; PDSS; ADHD-RS-IV; CGI-I) | no significant differences between the GXR and placebo groups on measures of psychomotor functioning or alertness from the CRT at endpoint (least-square mean difference: 2.5 [95% confidence interval (CI): 22.9, 28.0], p=0.8 for CRT; 2.5 [95% CI: 21.5, 26.4], p=0.84 for correct responses; 15.5 [95% CI: 45.1, 14.1], p=0.30 for movement time; and 8.2 [95% CI: 54.1, 37.6] p¼0.72 for total time). |
| ***Lerner***  ***2000*** | Clinical trial | Clonidine | 8 | None | HPPD | NA | 23.25±2.49 | None | **HPPD** symptoms (CGI-I, self-report scale) | Clonidine improved CGI from 5 to 2 score after 2 months of therapy and was well tolerated and the self-report scale was 2 for all patients, indicating very mild symptomatolgy. |
| ***Bilder2016, Loo2016, McCracken 2016 (NCT00429273)*** | RCT | Guanfacine | 68 | None | ADHD | 66.2 | 10.1±2.1 | None | **Cognitive functions** (ADHD-RS-IV and CGI-S and ADR: Physical symptoms checklist) | Improvement in ADHD symptoms; however, it appeared to slower reaction time. |
|  |  |  |  |  |  |  |  |  |  | It may be most effective for those individuals with ADHD who have high arousal or affective dysregulation, perhaps in line with the reported secondary effect of decreasing emotional lability and irritability. |
|  |  | d-Methylphenidate | 69 |  |  | 66.7 | 10.1±2 | None | **Cognitive functions** (ADHD-RS-IV and CGI-S and ADR: Physical symptoms checklist) | d-MPH leads to improvement in ADHD symptoms |
|  |  | Guanfacine + d-Methylphenidate | 70 |  |  | 72.9 | 9.9±2.2 | None | **Cognitive functions** (ADHD-RS-IV and CGI-S and ADR: Physical symptoms checklist) | Combination leads to improvement in ADHD symptoms as well as with improved cognitive functioning, such as fewer inattentive errors and lower reaction time variability. |
| ***Cutler 2014 and Wilens 2017 (NCT00734578)*** | RCT | Guanfacine (morning dose) | 150 | None | ADHD | 72 | 11.0±2.6 | Psychostimulants (Methylphenidate or Amphetamine) | **ADHD symptoms** (CGI-S; ADHD-RS-IV; CGI-P; BSFQ) | CGI-P scores improved with GXR (morning assessment, GXR AM, placebo-adjusted least squares [LS] mean = -1.7, GXR PM = -2.6; evening assessment, GXR AM = -2.4, GXR PM = -3.0).  BSFQ scores reflected improved morning functioning with GXR (GXR AM, placebo-adjusted LS mean = -5.1; GXR PM = -4.7). Results were similar for symptomatic remission (ADHD-RS-IV total score ≤ 18; 61.1%, 62.2%, versus 46.1%; p= .010 and p = .005, respectively) and syndromal remission (symptomatic remission plus CGI-S score ≤ 2). |
|  |  | Guanfacine (evening dose) | 152 |  |  | 69.7 | 10.6±2.3 | Psychostimulants (Methylphenidate or Amphetamine) | **ADHD symptoms** (CGI-S; ADHD-RS-IV; CGI-P; BSFQ) |  |
|  |  | Placebo | 153 |  |  | 73.2 | 10.8±2.3 | Psychostimulants (Methylphenidate or Amphetamine) | **ADHD symptoms** (CGI-S; ADHD-RS-IV; CGI-P; BSFQ) |  |
| ***Palumbo***  ***2008*** | RCT | Placebo | 30 | None | ADHD, ODD, Conduct Disorder | 76.7 | 9±1.5 | None | **ADHD symptoms** (ASQ-Teacher, ASQ Parent, Conners Continuous Performance Task, CGAS) | Clonidine was not found to be as effective as methylphenidate in reducing ADHD symptoms based on the ASQ-Teacher.    However, secondary outcome measures using parent (ASQ-Parent) ratings of ADHD symptoms and clinician ratings of global functioning suggested a benefit of clonidine.  Sedation was the most problematic side effect. |
|  |  | Methylphenidate | 29 |  |  | 82.8 | 9.4±1.6 | None | **ADHD symptoms** (ASQ-Teacher, ASQParent, Conners Continuous Performance Task, CGAS) |  |
|  |  | Clonidine | 31 |  |  | 87.1 | 9.4±1.2 | None | **ADHD symptoms** (ASQ-Teacher, ASQParent, Conners Continuous Performance Task, CGAS) |  |
|  |  | Clonidine+Methylphenidate | 32 |  |  | 75 | 10±2 | None | **ADHD symptoms** (ASQ-Teacher, ASQParent, Conners Continuous Performance Task, CGAS) |  |
| ***Sallee***  ***2009*** | RCT | Guanfacine 1 mg | 62 | None | ADHD | 67.2 | 9.3±2.14 | None | **ADHD symptoms** (ADHD-RS-IV, CPRS-R, CGI-S, CGI-I, PGA) | Statistically significant reductions in ADHD Rating Scale-IV scores were observed from baseline to endpoint at all doses of GXR.  ADHD-RS-IV mean total score from baseline to endpoint were significant for all GXR doses in the younger age group (6-12 years) but not for the older age group (13-17 years).  ADR: lethargy (0/65); sedation (6/65); somnolence (11/65); irritability (4/65) |
|  |  | Guanfacine 2 mg | 65 |  |  | 70.8 | 10.6±2.81 | None | **ADHD symptoms** (ADHD-RS-IV, CPRS-R, CGI-S, CGI-I, PGA) |  |
|  |  | Guanfacine 3 mg | 65 |  |  | 73.8 | 11.1±2.96 | None | **ADHD symptoms** (ADHD-RS-IV, CPRS-R, CGI-S, CGI-I, PGA) |  |
|  |  | Guanfacine 4 mg | 66 |  |  | 81.5 | 10.5±2.53 | None | **ADHD symptoms (**ADHD-RS-IV, CPRS-R, CGI-S, CGI-I, PGA) |  |
|  |  | Placebo | 66 |  |  | 68.2 | 10.8±2.89 | None | **ADHD symptoms** (ADHD-RS-IV, CPRS-R, CGI-S, CGI-I, PGA) |  |
| ***Scahill***  ***2001*** | RCT | Guanfacine | 17 | None | ADHD, tic disorders | 91.2 | 10.4±2 | None | **ADHD symptoms** (ADHD-RS, the hyperactivity index of the Parent Conners Questionnaire, CGI-I, The Yale Global Tic Severity Scale, The Children’s Yale-Brown Obsessive Compulsive Scale) | After 8 weeks of treatment guanfacine group: 37% drop in the total score on the ADHD Rating Scale completed by the teacher, compared to an 8% drop in the placebo group (t=3.61, df=32, p<0.001).  CGI-I: 9/17 subjects were rated much improved or very much improved, compared to 9/17 in the placebo group (p<0.001, Fisher’s exact test).  Guanfacine was also associated with a 31% drop in the total tic score of the Yale Global Tic Severity Scale, compared with 0% improvement in the placebo group (t=2.02, df=30, p=0.05).  The 27% improvement from baseline on the parent-rated hyperactivity index for the guanfacine group was not significantly different from the 21% improvement in the placebo group.  ADR: One subject in the guanfacine group withdrew from the study at week 4 due to sedation. Six other subjects also complained of mild sedation, which relented with continued treatment or dose decrease. Three subjects reported midsleep awakening during the dose escalation period. |
|  |  |  |  |  |  |  |  |  |  |  |
|  |  | Placebo | 17 |  |  |  |  | None | **ADHD symptoms (**ADHD-RS, the hyperactivity index of the Parent Conners Questionnaire, CGI-I, The Yale Global Tic Severity Scale, The Children’s Yale-Brown Obsessive Compulsive Scale) |  |
| ***Scahill***  ***2006*** | Clinical trial | Guanfacine | 25 | None | PDD, ASD | 92 | 9.03±3.14 | None | **ADHD symptoms (**ABC, CGI-I) | After 8 weeks of treatment, the parent-rated Hyperactivity subscale of the ABC went from a mean of 31.2 (±8.77) at baseline to 18.9 (±10.37) (effect size = 1.4; p < 0.001).  The teacher-rated Hyperactivity subscale decreased from a mean of 29.9 (±10.09) at baseline to 21.9 (±9.56) (effect size = 0.83; p < 0.01). 12 children (48%) were rated as Much Improved or Very Much Improved on the CGI-I.  Common adverse effects:  irritability, sedation, sleep disturbance (insomnia or midsleep awakening), and constipation. |
|  |  |  |  |  |  |  |  |  |  |  |
| ***Scahill***  ***2015*** | RCT | Guanfacine | 30 | None | ASD, PDD | 86.67 | 8.44±2.28 | None | **ADHD symptoms** (ABC, ADHD-RS, CGI-I) | The guanfacine group showed a 43.6% decline in scores on the ABC-hyperactivity subscale (least squares mean from 34.2 to 19.3) compared with a 13.2% decrease in the placebo group (least squares mean from 34.2 to 29.7; effect size=1.67).  The rate of positive response (much improved or very much improved on the CGI-I) was 50% (15 of 30) for guanfacine compared with 9.4% (3 of 32) for placebo.  A brief cognitive battery tapping working memory and motor planning showed no group differences before or after 8 weeks of treatment. |
|  |  | Placebo | 32 |  |  | 84.38 | 8.39±2.23 | None | **ADHD symptoms** (ABC, ADHD-RS, CGI-I) |  |
| ***Spencer***  ***2009*** | Clinical trial | Guanfacine + Methylphenidate | 42 | None | ADHD | 69.0 | 11.1±1.8 | None | **ADHD symptoms** (PDSS, PSERS, ADHD-RS-IV, CPRS-R, CGI-S, CGI-I, PGA, CHQ-PF50). | Fatigue (28.6%), irritability (14.3%), somnolence (14.3%), sedation (9.5%). Decrease in mean PDSS, PSERS.  Mean change from baseline to endpoint in AHDH-RS-IV total score: -17.8 (p > 0.0001), overall mean % reduction: 56.0%. 73% improvement in CGI. 84.1% improvement PGA.  CHQ-PF50 psychosocial score: 10.2 (p < 0,0001). |
|  |  | Guanfacine + Amphetamine | 33 |  |  | 78.8 | 11.6±2.6 | None | **ADHD symptoms** (PDSS, PSERS, ADHD-RS-IV, CPRS-R, CGI-S, CGI-I, PGA, CHQ-PF50). | Decrease in mean PDSS, PSERS. Mean change from baseline to endpoint in AHDH-RS-IV total score: -13.8 (p > 0.0001), overall mean % reduction: 56.0%. 73% improvement in CGI. 84.1% improvement PGA. CHQ-PF50 psychosocial score: 10.2 (p < 0,0001).  Fatigue (18.2%), irritability (33.3%), somnolence (24.2%), sedation (6.1%). |
| ***Stein***  ***2015*** | RCT | Guanfacine (morning dose) | 107 | None | ADHD | 67.3 | 9.1 | None | **ADHD symptoms** (WFIRS-P, ADHD-RS-IV, CGI-I) | Significant improvements from baseline in the placebo-adjusted difference in least-squares (LS) mean (95 %confidence interval)  WFIRS-P Total scores for both GXR treatment groups combined [GXR all-active: -0.16 (-0.25, -0.07), effect size (ES) = 0.448, P\0.001] and separately [GXR AM: -0.15 (-0.26, -0.05), ES = 0.417, P = 0.004; GXR PM: -0.18 (-0.28, -0.07), ES = 0.478, P = 0.001 |
|  |  | Guanfacine (evening dose) | 114 |  |  |  |  | None | **ADHD symptoms** (WFIRS-P, ADHD-RS-IV, CGI-I) |  |
|  |  | Placebo | 112 |  |  |  |  | None | **ADHD symptoms (**WFIRS-P, ADHD-RS-IV, CGI-I) |  |
| ***Symons***  ***2004*** | Case Report | Clonidine | 1 | None | ADHD, mild mental retardation | 0 | 13 | Methylphenidate; clomipramine | **ADHD symptoms** (SIT, SIG) | Reduction of number of self-injury body sites by 90%, complete resolved self-injury behaviour severity. |
| ***Taormina 2016*** | Case Report | Guanfacine | 2 | None | OCD, ADHD | 100 | Min-Max: 9-10 | Sertraline | **ADHD symptoms (**CYBOCS) | CYBOCS: significant improvement within 1-week, complete remission by week 18, stable at 1 year follow-up. |
| ***Taylor***  ***2001*** | Cross-Over trial | Placebo | 17 | None | ADHD | 41 | 41.2±11.4 | None | **ADHD symptoms** (DSM-IV ADHD Behavior Checklist for Adults and the COWAT). | Both drugs significantly reduced ADHD symptoms on the DSM-IV Adult Behavior Checklist for Adults over placebo (p < 0.05). The Stroop Color subscale showed significant improvement for both drugs (p < 0.05), but the Color-Word measures showed significant improvement for guanfacine only (p < 0.01). |
|  |  | Guanfacine |  |  |  |  |  |  | **ADHD symptoms** (DSM-IV ADHD Behavior Checklist for Adults and the COWAT). |  |
|  |  | Dextroamphetamine |  |  |  |  |  |  | **ADHD symptoms** (DSM-IV ADHD Behavior Checklist for Adults and the COWAT). |  |
| ***Tourette's Syndrome Study Group***  ***2002*** | RCT | Clonidine | 34 | None | ADHD, tic disorders, OCD, ODD, CD, GAD, MDD | 85 | 9.7±1.8 | Amitriptyline, clomipramine, desipramine, carbamazepine, gabapentin | **ADHD symptoms (**Conners Abbreviated Symptom Questionnaire--Teacher**)** | Significant improvement occurred for subjects assigned to Clonidine (p < 0.002) and those assigned to Methylphenidate (p < 0.003).  Compared with placebo, the greatest benefit occurred with combined Clonidine + Methylphenidate (p < 0.0001).  Clonidine appeared to be most helpful for impulsivity and hyperactivity.  Compared with placebo, measured tic severity lessened in all active treatment groups in the following order: Clonidine + Methylphenidate, Clonidine alone, Methylphenidate alone.  Sedation was common with Clonidine treatment (28% reported moderate or severe sedation), but otherwise the drugs were tolerated well, including absence of any evident cardiac toxicity. |
|  |  | Clonidine + Methylphenidate | 33 |  |  | 73 | 10.6±1.9 |  | **ADHD symptoms (**Conners Abbreviated Symptom Questionnaire--Teacher**)** |  |
|  |  | Methylphenidate | 37 |  |  | 92 | 10.7±2.0 |  | **ADHD symptoms (**Conners Abbreviated Symptom Questionnaire--Teacher**)** |  |
|  |  | Placebo | 32 |  |  | 91 | 9.7±1.8 |  | **ADHD symptoms (**Conners Abbreviated Symptom Questionnaire--Teacher**)** |  |
| ***Rakesh***  ***2011*** | Fixed-dose RCT | Clonidine 0.2 mg/day | 78 | None | ADHD | 58 | 9.4 | None | **ADHD symptoms** (ADHD-RS-IV total score; CGI-I; CGI-S; PGA**)** | Improvement from baseline in ADHD-RS-IV total score was significantly greater in both Clonidine-XR groups versus placebo at week 5.  A significant improvement in ADHD-RS-IV total score occurred between groups as soon as week 2 and was maintained throughout the treatment period.  Improvement in ADHD-RS-IV inattention and hyperactivity/impulsivity subscales, Conners Parent Rating Scale–Revised: Long Form, CGI-I, CGI-S, and PGA, occurred in both treatment groups versus placebo. |
|  |  | Clonidine 0.4 mg/day | 80 | None | ADHD | 55 | 9.4 | None | **ADHD symptoms** (ADHD-RS-IV total score; CGI-I; CGI-S; PGA**)** |  |
|  |  | Placebo | 78 | None | ADHD | 52 | 9.4 | None | **ADHD symptoms** (ADHD-RS-IV total score; CGI-I; CGI-S; PGA**)** |  |
| ***Okazaki***  ***2019*** | Case Report | Guanfacine | 1 | None | ADHD, ASD, Tourette’s Syndrome | 100 | 10 | None | **ADHD symptoms** | Symptoms of ADHD as well as tic symptoms were improved. The patient was able to continue taking Guanfacine for 6 more months (ADHD-RS-IV-J score of 9, YGTSS score of 15) |
| ***Van Stralen 2020*** | RCT | Guanfacine | 47 | None | ADHD | 84 | NA | Psychostimulants | **ADHD symptoms** (BRIEF-P; ADHD-RS IV; CGI-S; CGI-I scales) | Significant benefits of GXR plus psychostimulant were observed on BRIEF-P (p value = .0392), ADHD-RS-IV (p < .0001), CGI-S (p = .0007), and CGI-I (p = .003). There were no serious adverse events |
|  |  | Placebo | 48 |  |  | 85 | NA | Psychostimulants | **ADHD symptoms** (BRIEF-P; ADHD-RS IV; CGI-S; CGI-I scales) |  |
| ***Yadav***  ***2021*** | Case Report | Clonidine | 1 | None | Schizophraenia | 100 | 55 | Clozapine | **ADHD symptoms (**WFIRS-P, ADHD-RS-IV, CGI-I) | New onset behavioural problems (sexual disinhibition, impulsivity) |
| ***Ye***  ***2018*** | Case Report | Clonidine | 1 | None | ADHD, disruptive mood dysregulation, Anxiety, social phobia | 100 | 14 | None | **Anxiety** (CGI-S, BAI) | CGI-S score from 5 (markedly ill) at start to 2 (borderline mentally ill) after 2 months, BAI score from 49 at start to 4 after 2 months |
| ***Ye***  ***2019*** | Case Report | Clonidine | 1 | None | MDD, generalized anxiety, ADHD, learning disorder, PTSD | 100 | 12 | Aripiprazole; Fluvoxamine; Lamotrigine | **PTSD symptoms** (BDI, BAI) | BDI score from 28 at start to 7 after 4 weeks, BAI score from 18 to 14 after initiation.  PTSD symtoms decreased, improved scholarship, socialization, relationships. |
| ***Alpha-Blockers*** | | | | | | | | | | |
| ***Aggarwal***  ***2020*** | Case Report | Prazosin | 2 | None | Substance use disorder, CD, MDD | 100 | 15.5 |  | **Drug dreams symptoms** | Good response and tolerability |
| ***Ahmadpanah***  ***2014*** | RCT | Hydroxyzine | 34 | None | PTSD | 74 | 36.12±6.05 | Lorazepam, clonazepam, sertraline, alprazolam | **Sleep Quality** (Pittsburgh Sleep Quality Index; Mini International Neuropsychiatric Interview; PTSD scores) | Sleep duration, (h): 4.29±1.45 vs 5.41±1.64; sleep onset latency, min 80.50±41.85 vs 40.68±18.71; sleep quality (1 = high, 3 = low) 2.21±0.81 vs 1.53±0.56; nightmares (1 = good dreams, 3 = bad dreams) 2.50±0.86 vs 1.29±0.80; overall PSQI score 15.56±2.23 vs 12.21±2.07; PTSD score 7±0.05 vs 3.58±0.98 |
|  |  | Placebo | 33 |  |  | 67 | 34.21±6.05 | Lorazepam, clonazepam, sertraline, alprazolam | **Sleep Quality (**Pittsburgh Sleep Quality Index; Mini International Neuropsychiatric Interview; PTSD scores) | Sleep duration, (h): 4.58±1.23 vs 4.79±1.22; Sleep onset latency, min 73.70±33.48 vs 54.97±27.36; (1 = high, 3 = low) 2.21±0.81 vs 01.08±0.63; Nightmares (1 = good dreams, 3 = bad dreams) 2.48±0.91 vs 2.30±0.95; Overall PSQI score 15.48±1.97 vs 15.03±1.69; PTSD score 7±0.40 vs 5.39±1.29 |
|  |  | Prazosin | 33 |  |  | 76 | 36.18±7.09 | Lorazepam, clonazepam, sertraline, alprazolam | **Sleep Quality** (Pittsburgh Sleep Quality Index; Mini International Neuropsychiatric Interview; PTSD scores) | Sleep duration, (h): 4.39±1.62 vs 6.12±1.27; Sleep onset latency, min 51.64±53.39 vs 31.91±27.4; Sleep quality (1 = high, 3 = low) 2.39±0.79 vs 1.48±0.83; Nightmares (1 = good dreams, 3 = bad dreams) 2.42±0.97 vs 0.85±1.03; Overall PSQI score 15.52±2.17 vs 10.21±2.18; PTSD score 7±0.04 vs 3.36±0.78 |
| ***Byers***  ***2010*** | Observational Study | Prazosin | 62 | None | PTSD | 98 | 54 | Benzodiazepines, sleep agents, SSRI | **Nighttime symptoms** | Patients were significantly more likely to continue their therapy to study end date compared with quetiapine (48.4% vs 24%; P<0.001; odds ratio, 3.0; 95% confidence interval, 1.62-5.45), thus achieving long-term effectiveness |
|  |  | Quetiapine | 175 |  |  | 98 | 53 | Benzodiazepines, sleep agents, SSRI | **Nighttime symptoms** | Patients were more likely to discontinue therapy because of adverse effects compared with the prazosin group (34.9% vs 17.7%; P = 0.008) |
| ***Calohan***  ***2010*** | Clinical Trial | Prazosin | 13 | None | PTSD | 84.6 | 27 | Zolpidem, Sertraline, Citalopram, Clonazepam, Quetiapine, Hydroxyzine | **PTSD‐related nightmares and sleep disturbance (**CAPS) | The CAPS “distressing dreams” decreased from M = 7.0 (SD = 0.7) to M = 2.9 (SD = 3.0), paired (12) = 5.2, p < .001.  CAPS “difficulty falling and staying asleep” (item D1) decreased from M = 6.7 (SD = 0.9) to M = 3.7 (SD = 2.4), (12) = 6.6, p < .001.  Prazosin can be used successfully to treat trauma nightmares and sleep disturbance in military personnel engaged in combat operations |
|  |  |  |  |  |  |  |  |  |  |  |
| ***Diokpa***  ***2021*** | Retrospective Study | Prazosin | 84 | None | PTSD+Depression/Anxiety/Psychosis/BD | 71 | 39 | Antidepressants; trazodone; hypnotics; gabapentin; antihypertensive; | **PTSD‐related nightmares** (PCL and Clinician Administered PTSD Scale) | Nightmare severity (before intervention PCL:4/5, after starting prazosin: 3.19/5 a significant decrease in repeated distressing dreams), nightmare frequency (from 4 to 2 times per week on average |
|  |  |  |  |  |  |  |  | hydroxyzine; benzodiazepines; antipsychotics; mood stabilizers; psychostimulants |  |  |
| ***Ferrafiat***  ***2020*** | Retrospective Study | Prazosin | 18 | None | PTSD+MDD/Anxiety disorder | 44.4 | 13±1.8 | SSRI, antipsychotics and Carbamazepine | **PTSD symptoms** (UCLA-PTSD-RI scores, CGI-S) | CGI-S score significantly decreased from 5.3 (± 0.9) to 2.9 (± 0.7).  The mean total UCLA-PTSD-RI score significantly decreased 11.4 points (± 5.4) during the first week and 37.9 (± 16) during the first month, leading to an improvement of 20% and 67%, respectively. |
| ***Germain***  ***2012*** | RCT | Prazosin | 18 | None | PTSD+Anxiety/MDD/Insomnia | 88.9 | 39.4±11.9 | NA | **Sleep and daytime symptoms** (Insomnia Severity Index, PSQI Addendum for PTSD, and the PghSD) | Both active treatment groups showed greater reductions in insomnia severity and daytime PTSD symptom severity.  Reductions on insomnia severity as measured by the ISI were significantly greater in the BSI and prazosin groups compared to the placebo group post-treatment (F (2, 37)=6.06, p<.01). |
|  |  | Behavioural Intervention | 17 |  |  | 82.4 | 40.0±14.2 | NA | **Sleep and daytime symptoms** (Insomnia Severity Index, PSQI Addendum for PTSD, and the PghSD) |  |
|  |  | Placebo | 15 |  |  | 100 | 43.6±14.0 | NA | **Sleep and daytime symptoms** (Insomnia Severity Index, PSQI Addendum for PTSD, and the PghSD) |  |
| ***Gopalakrishna***  ***2016*** | Case Report | Prazosin | 2 | None | BD I, substance use disorder | 50 | 33 | Lithium, olanzapine | **Drug dreams symptoms** | Drug dreams symptoms resolved after the addition of prazosin during an acute psychiatric hospitalization |
| ***Petrakis***  ***2016*** | RCT | Prazosin | 50 | None | PTSD + alcohol dependence | 92 | 44.5±13.2 | NA | **PTSD symptoms** (CAPS, TLFB, OCDS, PSQI) | Prazosin is not effective in treating PTSD symptoms, improving sleep, or reducing alcohol consumption |
|  |  | Placebo | 46 |  |  | 95.56 | 43.4±12.95 | NA | **PTSD symptoms** (CAPS, TLFB, OCDS, PSQI) |  |
| ***Koola M***  ***2014*** | Case Report | Prazosin | 1 | None | PTSD +treatment-resistant mood disorders | 0 | 50 | antidepressants, neuroleptics | **PTSD symptoms** (hyperarousal, flashbacks and daytime re-experiencing of the trauma and nightmares) | Significant improvement of PTSD symptoms in conjunction with improvement in comorbid depression and anxiety symptoms with rational combination therapy |
|  | Case Report | Prazosin | 1 | None | PTSD +treatment-resistant mood disorders | 0 | 46 | antidepressants, neuroleptics | **PTSD symptoms (**hyperarousal, flashbacks and daytime re-experiencing of the trauma and nightmares) | Significant improvement of PTSD symptoms in conjunction with improvement in comorbid depression and anxiety symptoms with rational combination therapy |
| ***Racin***  ***2014*** | Case Report | Prazosin | 1 | None | ADHD + PTSD | 100 | 10 | Fluoxetine and MPH | **PTSD‐related nightmares** | Complete resolution of nightmares |
| ***Srivastava 2020*** | Case Report | Prazosin | 1 | None | Acute Stress Disorder | 100 | 51 | Methadone | **Acute stress disorder** | Complete resolution of nightmares, anxiety and flashbacks |
| ***ARBs*** | | | | | | | | | | |
| ***Cody***  ***2020*** | Case Report | Valsartan | 1 | hypertension | None | 100 | 61 | None | **Anxiety symptoms** | Significant improvement in anxiety symptoms of generalized type, such as initial insomnia, palpitations, increased respiratory rate, worrying about missing work and fear of not waking up in time to get to work. |
| ***Dereli***  ***2020*** | Cross-sectional Study | Sacubitril/Valsartan | 115 | HFrEF+Atrial fibrillation/Hypertension | None | 75 | 64 ± 17 | NA | **Depression and anxiety**  **(**BDI-II, BAI, 6-MWT) | BDI-II and BAI scores when compared before and after the sacubitril/valsartan treatment: 13.7 ± 9.7 to 7.6 ± 3.8, p<0.001 and 13.3 ± 8.9 to 8.1 ± 4.1, p<0.001, respectively.  The 6-MWT distance significantly increased from 213 ± 95 to 327 ± 118 mt (p<0.001). |
| ***Fan***  ***2017*** | RCT | Telmisartan | 22 | None | Schizophraenia, schizoaffective disorder | 82 | 41.5 ± 12.3 | Clozapine,  Olanzapine | **Cognitive performance** (PANSS, SANS and a neuropsychological battery) | PANSS total score: mean SD: - 4.1 (8.1). Statistically significant difference between groups: P = 0.038, SCohen’s d = 0.57. No significant differences between the two groups in change from baseline to week 12 in PANSS subscale scores, SANS total score, or any cognitive measures (P > 0.100) |
|  |  | Placebo | 21 |  |  | 76 | 44.4 ± 11.5 | Clozapine, Olanzapine | **Cognitive performance** (PANSS, SANS and a neuropsychological battery) | PANSS total score: mean SD: 0.4 (7.5). Statistically significant difference between groups: P = 0.038, SCohen’s d = 0.57.  No significant differences between the two groups in change from baseline to week 12 in PANSS subscale scores, SANS total score, or any cognitive measures (P > 0.100) |
| ***Lin***  ***2020*** | Retrospective matched-cohort study | Controls (No ARBs) | 40976 | Hypertension | Suicidal attempts | 54.9 | 53.3 (45.3;63.3)* | BDZ, NON-BDZ, Antipsychotics, Fluoroquinolones, Statins, Oral Antihypertensive drugs, alpha-blockers, beta-blockers, Diuretics, CCB, ACE-I, Others | **Incidence of suicide attempts** | Lower incidence of suicide attempts in ARBs users, especially men over 50 |
|  |  | ARBs | 40976 |  |  | 54.9 | 53.1 (45.1;63.3) ^*^ | BDZ, NON-BDZ, Antipsychotics, Fluoroquinolones, Statins, Oral Antihypertensive drugs, alpha-blockers, beta-blockers, Diuretics, CCB, ACE-I, Others | **Incidence of suicide attempts** | Lower incidence of suicide attempts in ARBs users, especially men over 51 |
| ***Saxby***  ***2008*** | RCT | Candesartan | 112 | Hypertension | None | 40.7 | 76± 4 | HCTZ, aspirin, warfarin, NSAIDs, psychotropic drugs | **Cognitive decline** (MMSE ≤24) | Less decline in attention (0.004 vs 0.036, p   0.04) and episodic memory (0.14 vs 0.22, p   0.04) compared to placebo, a similar trend for speed of cognition (2.3 vs 17.4, p 0.15), but no differences in working memory (0.0014 vs 0.0010, p 0.90) or executive function (0.0031 vs 0.0023, p 0.95) |
|  |  | Placebo | 116 |  |  | 40.5 | 76±5 |  | **Cognitive decline** (MMSE ≤24) |  |
| ***β-blockers*** | | | | | | | | | | |
| ***Ahmed***  ***2010*** | Case Report | Metoprolol | 1 | Hypertension; angina pectoris | None | 100 | 81 | Acetylsalicylic acid, pantoprazole, oxazepam, valproic acid | **Safety outcome** | Sleep disorders, nightmares, depression and anxiety |
| ***Al-Dury***  ***2021*** | Case Report | Propanolol | 1 | Small esophageal varices | None | 0 | 65 | Lactulose, rifamixin and benzodiazepines | **Safety outcome** | Visual and auditory hallucinations after a few months of treatment |
| ***Alexander 2007*** | Cross-Over Trial | Placebo+control | 16 | None | Stress induced in healthy volunteers | 50 | 23.81±4.69 | None | **Cognitive flexibility performance** (CRA, CFT, GP) | Propranolol reversed stress-induced impairment of cognitive flexibility, improving performance levels to within the range achieved during the control conditions (CRA:7.94±0.60).  Propranolol enhanced the ability to solve CRA problems (CRA:8.19±0.70) during stress as compared to placebo (P:0.05), anagrams: 28.65±1.89 (trend-to significant decrease with propranolol during stress compared to placebo P:0.05).  During stress, neither visuospatial memory, nor motor speed was affected by propranolol (Rey CFT: 22.03±1.80, grooved pegboard:65.57±4.02) |
|  |  | Placebo+stress |  |  |  |  |  |  | **Cognitive flexibility performance** (CRA, CFT, GP) |  |
|  |  | Propranolol+control |  |  |  |  |  |  | **Cognitive flexibility performance** (CRA, CFT, GP) |  |
|  |  | Propranolol+stress |  |  |  |  |  |  | **Cognitive flexibility performance** (CRA, CFT, GP) |  |
| ***Alsini***  ***2021*** | Cross-sectional Study | Beta-blockers | 3326 | None | Anxiety, schizopharenia, depression, OCD | 33 | 21.9±1.7 | NA | **Safety outcome, Anxiety** (GAD-7 score) | Significant positive correlation between the Beta-blockers usage and the GAD-7 scores |
| ***Armstrong 2020*** | Retrospective study | Atenolol | 92 | None | PTSD, OTSRD, anxiety disorder, GAD, SAD, adjustment disorder, MDD | 71 | Min-Max: 19-51 | NA | **Symptoms of anxiety** | 79 (86%) out of 92 patients had a positive effect. |
| ***Avital***  ***2009*** | RCT | Zolmitriptan | 19 | None | Schizophraenia/schizoaffective disorder + acute neuroleptic-induced akathisia | 71.4 | 38.0±12.0 | Neuroleptic, benzodiazepines, anticholinergics, mood stabilizers, antidepressants, ACEIs, bezafibrate, mercaptizole | **Acute neuroleptic-induced akathisia (**BARS, HAMD, HAMA, SAS, PANSS**)** | After 3 days of treatment BARS:8.13±3.6 (decreased by 2.85 points).  HAMD day 0: 17.42±2.99, HAMD day 3: 14.71±2.63;  HAMA day 0: 29.42±8.14, HAMA day 3: 27.14±7.10;  SAS day 0: 16.70±2.14, SAS day 3: 17.00±4.65;  PANSS day 0: 88.29±20.76, PANSS day 3: 82.86±19.60  Significant reduction in scores of all scales. |
|  |  | Propranolol | 14 |  |  | 57.9 | 35.2±9.26 |  | **Acute neuroleptic-induced akathisia (**BARS, HAMD, HAMA, SAS, PANSS**)** | After 3 days of treatment BARS:6.3±3.8 (decreased by 4.07 points).  HAMD day 0: 15.33±7.77, HAMD day 3: 12.66±5.85;  HAMA day 0: 29.33±9.92, HAMA day 3: 26.00±6.57;  SAS day 0: 15.08±2.5, SAS day 3: 13.9±2.5; PANSS day 0: 89.83±12.17, PANSS day 3: 87.00±10.06  Significant reduction in scores of all scales. |
| ***Beversdorf 2008*** | Cross-over double-blind trial | Propranolol | 18 (9 autistic patients+ 9 controls) | None | ASD | Autistic patients: 88.9 | Autistic patients: 29.2±9.9 Controls: 23.4±2.2 | None | **Verbal problem solving** | Compared to placebo: propranolol significantly improved performance on category fluency, but not letter fluency. |
|  |  | Placebo |  |  |  | Controls: 77.8 |  |  | **Verbal problem solving** |  |
| ***Beversdorf 2011*** | Cross-Over, case-matched Trial | Propranolol | 28  (14 autistic patients + 14 controls) | None | ASD | 71.4 | Autistic patients:18.9±2.9 | None | **Word Fluency** (letter fluency task, category fluency task, correlation with vocabulary score, correlation with WASI IQ) | Letter fluency task (22.4±6.0), category fluency task (34.3±10.5), no correlation between vocabulary score and the effect of propranolol, no correlation between WASI IQ and effect of propranolol. |
|  |  | Placebo |  |  |  |  | Controls: 19.4±2.0 |  | **Word Fluency** (letter fluency task, category fluency task, correlation with vocabulary score, correlation with WASI IQ) | Letter fluency task (21.9±7.1), category fluency task (30.1±8.9) |
| ***Bodner***  ***2012*** | Cross-Over, case-matched Trial | Propranolol | 27  (14 autistic patients + 13 controls) | None | ASD | Autistic patients:71.4  Controls: 69.2 | Autistic patients:18.9  Controls: 19.2 | None | **Working memory, inhibitory control, general sustained attention ability** | Working memory (mean error rate 18.5%). Inhibitory control (mean error rate: 15.4%). General sustained attention ability (AX mean error rate: 6.2%; in BY condition ropranolol had no apparent effect on BX trial performance for the non-ASD group) |
|  |  | Placebo |  |  |  |  |  |  | **Working memory, inhibitory control, general sustained attention ability** | Working memory (mean error rate: 3.1%), propranolol was not associated with significant improvements. Inhibitory control (mean error rate: 9.7%). General sustained attention ability (AX mean error rate: 2.6%; in BY condition individuals with ASD made more errors than those non-ASD comparison group) |
| ***Burkauskas***  ***2016*** | Cross sectional study | Metoprolol | 646 | CAD, arterial hypertension | Depression,  anxiety | 73 | 57.8±9 | Nitrates, statins, benzodiazepines | **Cognitive Functions** (DSST) | BB use was associated with greater risk for impaired performance on the DSST (odds ratio=4.43; 95% confidence interval=1.02–19.20; p=0.047) |
|  |  | Controls | 76 |  |  | 72 | 58.4±10 | Nitrates, statins, benzodiazepines | **Cognitive Functions** (DSST) |  |
| ***Butt***  ***2017*** | Case-matched study | β-blockers | 12.147 | None | Exam-related anxiety | 19.7 | 19 (18–22)^*^ | None | **Risk of antidepressant/antipsychotropic use** | Risk of antidepressant use (adjusted HRs, 1.68 [95% confidence intervals (CIs), 1.57–1.79], p < 0.0001), other psychotropic medication use (HR, 1.93 [95% CI, 1.76–2.12] p < 0.0001), and suicide attempts (HR, 2.67 [95% CI, 1.04–6.82] p = 0.04). |
|  |  | Controls | 12.147 | None | None | 19.7 | 19 (18–22)^*^ | None | **Risk of antidepressant/antipsychotropic use** |  |
| ***Chang***  ***2013*** | Case-control study | Propranolol | 760 | hypertension + Ischemic stroke/ischemic heart disease | None | 47.2 | 73.3±6.3 | Diuretics, CCB, ACEIs, ARBS, alpha-blockers | **Risk of insomnia** | Using propanolol as reference: aOR (95% CI) for the insomnia risk was 0.47 (0.35-0.63) for non-propanolol users, 0.31 (0.19-0.50) for bisoprolol, 0.46 (0.33-0.66) for atenolol, and 0.48 (0.36-0.34) for β1-blockers. |
|  |  | Non-propanol users | 3303 |  | None | 50.9 | 72.3±6.1 |  | **Risk of insomnia** |  |
| ***Deepmala 2014*** | Case Report | Propranolol | 1 | None | Autism | 100 | 13 | Risperidone | **Hypersexual behaviors** | After 2 weeks of starting propanolol therapy, the number of school hypersexual incidents decreased to 20 per week (baseline 70 in 5 days) and after 3 months only 1 incident in a 2-month period |
| ***Fisher***  ***2002*** | Case Report | Metoprolol | 1 | Hypertension, CAD | None | 100 | 89 | Aspirin, isosorbide mononitrate, glyceryl trinitate transdermal | **Safety outcome** | Confusion, disorientation and agitation, speech incoherent, hallucinations |
|  | Retrospective chart review | Metoprolol | 24 | Hypertension, CAD, Cardiac arrhythmias | Depression | 50 | 71.8 | Prazosin, nitrates, methyldopa, perindopril, CCBs, diuretics, digoxin, sertraline, fluoxetine, laxatives | **Safety outcome** |  |
| ***Hegarty***  ***2017 & 2020*** | Cross-Over, case-matched Trial | Placebo | 26 | None | ASD | 84.6 | Autistic patients:  22.21±4.16 | None | **FC in task-related networks during cognitive processing** | Compared to placebo, propranolol was associated with decreased FC in the dorsal medial prefrontal cortex subnetwork of the default mode network and increased FC in the medial temporal lobe subnetwork, regardless of diagnosis.  These effects were not seen with nadolol suggesting that the alterations in FC following propranolol administration were not exclusively due to peripheral cardiovascular effects. |
|  |  | Propranolol | (13 ASD patients and 13 controls) |  |  |  | Controls: 22.86±2.68 |  | **FC in task-related networks during cognitive processing** |  |
|  |  | Nadolol |  |  |  |  |  |  | **FC in task-related networks during cognitive processing** |  |
| ***Kogoj***  ***2004*** | Case Report | Propranolol | 1 | None | Paranoid delusion | 100 | 74 | Olanzapine, ciproterone | **Safety outcome** | Delirium |
| ***Kroes***  ***2010*** | Clinical trial | Propranolol | 12 | None | None | 58.3 | 24.4 | None | **Emotional memory tests** | Propranolol reduced the declarative emotional memory enhancement for emotional items. The effect persists after 24 hoors, without a new administration of propranolol. |
|  |  | Placebo | 12 |  |  |  |  |  | **Emotional memory tests** |  |
| ***Maebara 2002*** | Case Report | Carvedilol | 1 | None | Panic disorder and anxiety | 100 | 41 | Alprazolam, etizolam | **Safety outcome** | Nightmares and sleep disorders because of overdose. |
| ***Mahabir 2016*** | RCT | Propranolol | 20 | None | PTSD | NA | 45.2±10.7 | Anxiolitics, antidepressant, antipsychotics | **PTSD severity changes** (IES-R)**; Cognitive function t** (Wechsler Abbreviated Scale of Intelligence) | After 2 hours, propranolol enhanced visual perception during a processing speed test.  After 7 days, symptoms severity decreased within groups but not significantly different from placebo (p=.75). |
|  |  | Placebo | 21 |  |  | NA | 41.7±12.5 |  | **PTSD severity changes** (IES-R)**; Cognitive function t** (Wechsler Abbreviated Scale of Intelligence) |  |
| ***Shahrbabaki 2013*** | Case Report | Propranolol | 1 | None | BD, panic disorder | 100 | 11 | Benzodiazepines | **Safety outcome** | Mania (euphoria, restlessness, insomnia, increased energy) |
| ***Zamzow 2016*** | Cross-Over double-blind trial | Propranolol | 20 (tot) | None | ASD | 95 | 21.39±4.55 | SSRI, atypical antipsychotics | **Conversational reciprocity** (autonomic activity and HAMA) | Compared to the placebo: propranolol significantly improved performance on the conversational reciprocity task total [d = 0.40] and nonverbal communication domain scores. Neither autonomic activity nor anxiety was significantly associated with drug response |
|  |  | Placebo |  |  |  |  |  |  | **Conversational reciprocity** (autonomic activity and HAMA) |  |
| ***Zhao 2013*** | Case Report | Metoprolol | 1 | Hypertension | None | 100 | 21 | L-amlodipine; isosorbide mononitrate | **Safety outcome** | Insomnia, anxiety, fears, distraction, confusion, disorientation, incoherent speech, auditory hallucinations, acute delirium. |
| ***CCBs*** | | | | | | | | | | |
| ***Burdick***  ***2020*** | Clinical trial | Isradipine | 10 | None | Schizophraenia, schizoaffective disorder | 70 | 38 ± 13.4 | Antipsychotics | **Neurocognition domains** (SANS, BPRS, HDRS, MCCB, UPSA, and Quality of Life Scale) | No clear benefit on neurocognition but a positive effect (baseline mean = 6.8 ± 1.3 to week 4 mean = 7.9 ± 1.1; t = 2.91, p = 0.017) on functional capacity was noted.  Side effects (change since baseline report): dizziness (1 new incidence at week 4); difficulty sleeping (2 new incidences at week 4); and decreased energy (3 new incidences at week 4, 2 of which reported difficulty sleeping as well). |
| ***Dikici***  ***2012*** | Case Report | Amlodipine | 1 | Hypertension + Ischemic stroke | None | 100 | 63 | Acetylsalicylic acid | **Safety outcome** | Delirium |
| ***Ostacher***  ***2014*** | Clinical trial | Isradipine | 10 | None | BD | 70 | 41.8 ± 12.1 | Anticonvulsants, lithium, benzodiazepine, antipsychotics, antidepressants | **Bipolar depression symptoms** (MADRS, YMRS, CGI-S, SAFTEE, QIDS-SR, CPFQ) | Improvement: MADRS: 2.1 (standard error = 0.36) points/week (p < 0.001); QIDS-SR: [1.0 (SE = 0.19) points/week; p < 0.001], CGI-S [0.18 (SE = 0.04) points/week; p < 0.001]; CPFQ, [0.67 (SE = 0.25) points/week; p = 0.007].  No significant change in YMRS was detected [mean decrease 0.13 (SE = 0.13) points/week; p = 0.30] |
| ***Ponciano***  ***2000*** | RCT | Nisoldipine | 15 | None | None | NA | >18 | NA | **Cognitive and Psychomotor functions** (CFF, DSST, MRT, LC) | No significant differences between treatments: CFF (F(1,28) =0.004), CRT (F(1,28) =0.12); RRT (F(1,28) = 0.003); MRT (F(1,28)= 0.35); DS (F(1,28) =0.04); DSST errors (F(1,28)=0.13), LC Task time (F(1,28) = 1.83), |
|  |  | Placebo | 15 |  |  |  |  |  | **Cognitive and Psychomotor functions** (CFF, DSST, MRT, LC) |  |
| ***Silverstone 2000*** | Retrospective study | Diltiazem | 8 | None | BD | 0 | >18 | Imipramine, carbamazepine, moclobemide, chlorpromazine, clonazepam, paroxetine, sodium valproate, lithium, thyroid hormone | **Manic and depressive episodes** (DSM-IV) | Pre- and post-diltiazem use:  statistically significant decrease in the frequency and severity of both manic and depressive episodes (p < 0.001). |
| ***Vahdani***  ***2020*** | RCT | Isradipine | 19 | None | Schizofrenia | 63.2 | 35.5 ± 7 | 1st generation antipsychotics, 2nd generation antipsychotics, antipsychotics combination, antidepressants | **Neurocognitive Functioning** (Wechsler Adult Intelligence Scale-Third Edition; Stroop, Wisconsin, Digit Symbol) | In comparison to the placebo group: Wechsler memory scale (verbal memory), significantly higher scores (P 0.002 and P 0.005 for the raloxifene and the isradipine group, respectively); Wisconsin test: substantial score improvement regarding the preservative response in both the raloxifene (P 0.064, Cohen d 3.44) and the isradipine (P 0.072, Cohen d 3.3); Stroop test, the isradipine group: post commission, congruent (P 0.025), post commission, incongruent (P 0.027), and post interference time (P0.036) |
|  |  | Raloxifene | 19 |  |  | 47.4 | 40 ± 7.8 |  | **Neurocognitive Functioning** (Wechsler Adult Intelligence Scale-Third Edition; Stroop, Wisconsin, Digit Symbol) |  |
|  |  | Placebo | 22 |  |  | 31.8 | 38.7 ± 7.3 |  | **Neurocognitive Functioning** (Wechsler Adult Intelligence Scale-Third Edition; Stroop, Wisconsin, Digit Symbol) |  |
| ***Wisner***  ***2002*** | Clinical trial | Verapamil | 37 | None | BD | 0 | >18 | None | **Bipolar disorders symptoms** (MRS, HRSD) | 39% of depressed responded, 100% of manic responded, patients with mixed states responded 100% on mania and 29% on depression. |
| ***Diuretics*** | | | | | | | | | | |
| ***Akhondzadeh***  ***2002*** | RCT | Diazoxide | 21 | None | Schizophraenia | 67 | Min-Max: 18-42 | Haloperidol | **Schizophraenia symptoms (**PANSS**)** | No significant difference between groups was observed (p 0.96). Both groups showed a significant improvement over the 8 weeks of treatment (Greenhouse–Geisser corrected: F 197.41, d.f. 2.88, P < 0.0001) |
|  |  | Placebo | 21 |  |  | 62 |  |  | **Schizophraenia symptoms (**PANSS**)** |  |
| ***Cheng***  ***2021*** | Clinical Trial | Bumetanide | 6 | None | ASD | 6 | 6.5 | Potassium | **ASD symptoms (**PANSS, CARS, CGI-S**)** | Positive results in 6 patients: according to the PASS—measuring symptom change—particularly with regard to “Communicative and cognitive abilities”. |
| ***Du***  ***2015*** | Clinical Trial | Controls (ABA) | 28 | None | ASD | 85.7 | 4.50±1.67 | None | **ASD symptoms** (ABC, CARS, and CGI) | Combination treatment group: statistically significant (p < 0.05) better treatment scores on the ABC and CGI, and a nonsignificant better treatment outcome on the CARS |
|  |  | Bumetanide+ ABA | 32 |  |  | 84.4 | 4.60±1.90 |  | **ASD symptoms** (ABC, CARS, and CGI) |  |
| ***Feng, J. Y 2020*** | Case Report | Bumetanide + Vitamin D3 | 1 | None | ASD | 0 | 30 months | None | **ASD symptoms (CARS)** | The patient’s symptoms remained unchanged after 6 months of Vitamin D3 supplementation, and her serum 25 (OH) D levels had reached 52.4ng/mL.  At the parent’s request, Vitamin D3 supplementation was discontinued because of lack of effectiveness. Thereafter, bumetanide was initiated.  A week after bumetanide initiation: positive language development, hyperactivity reduced.  After 1 month: patient’s behavior significantly improved. After 40 weeks, the improvement is confirmed by a CARS score =20. |
|  |  |  |  |  |  |  |  |  |  |  |
| ***Hadjikhani 2015*** | Clinical Trial | Bumetanide | 7 | None | ASD | 100 | 19.3±4.6 | None | **ASD symptoms** (WASI, TAS-20, magnetic resonance imaging and neuropsychological testing) | Significantly improved overall accuracy in emotion matching of faces with 40% intensity to their 100% intensity counterpart (overall mean accuracy (% correct) ± SD before: 62.5 ± 21.0; after: 75.0 ± 13.5; p 0.04). Bumetanide treatment also significantly improved overall RT for face emotion matching (overall mean RT (seconds) ± SD before: 8.18 ± 3.42; after: 5.82 ± 1.54; p 0.04). |
| ***Hadjikhani 2018*** | Clinical Trial | Bumetanide | 9 | None | ASD | 88.9 | 21.4±5.4 | None | **ASD symptoms** (Anatomical and functional MR images, Tobii T120 eye-tracking system and Tobii Studio 2.0.6) | Bumetanide reduces threat response to eye contact and increase time spontaneously spent looking in the eyes, potentially allowing acquisition of the necessary information to improve social processing |
| ***Lemonnier 2012*** | RCT | Bumetanide | 30 | None | ASD | 89 | 6.88±1.1 | None | **ASD symptoms** (ADOS, CARS, and CGI) | Compared to placebo: bumetanide reduced significantly the CARS (D90-D0; p < 0.004 treated vs placebo), CGI (p< 0.017 treated vs placebo) and ADOS values when the most severe cases (CARS values above the mean±sd.; n=9) were removed (Wilcoxon test: p 0.031; Student’s t-test: p 0.017 |
|  |  | Placebo | 30 |  |  | 74 | 7.11±1.8 |  | **ASD symptoms** (ADOS, CARS, and CGI) |  |
| ***Lemonnier 2013*** | Case Report | Bumetanide | 1 | No | Fragile X syndrome (ASD) | 100 | 10 | None | **ASD symptoms** (ADOS, ABC, CARS, RDEG and RRB) | The CARS, ADOS, ABC, RDEG and RRB scores improved. The CARS, total severity score fell by 6 points and 12 items that were equal, or above, three at baseline fell to 7. The ABC, RDEG and RRB scores were divided by 1.5 or 2 in 3 months, and the total ADOS score was reduced by 9 points, especially in sub-domains B (reciprocal social interactions) and C (play). |
| ***Lemonnier 2016*** | Case Report | Bumetanide | 1 | No | Schizophraenia | 100 | 14 | Risperidone, melatonin | **Schizophraenia symptoms** | Bumetanide reduced the severity of symptoms. After 1.5 years of bumetanide, he stopped the therapy. Hallucinations reappeared after two months and he decided to return to bumetanide and risperidone. |
| ***Lemonnier 2017*** | RCT | Bumetanide (1 mg) | 20 | None | ASD | 80 | 7.8±4.15 | None | **ASD symptoms** (SRS, CARS, and CGI) | CARS change, mean (sd) = -4.98 (4.33); SRS change, mean (sd) = − 12.36 (23.57); CGI (Very much improved) change, mean (sd) = 0 |
|  |  | Bumetanide (2 mg) | 23 |  |  | 95.7 | 7.87±4.58 |  | **ASD symptoms** (SRS, CARS, and CGI) | CARS change, mean (sd) = -3.74 (3.28); SRS change, mean (sd) = − 13.17 (20.45); CGI (Very much improved) change, mean (sd) = 0 |
|  |  | Bumetanide (4 mg) | 22 |  |  | 95.5 | 8.45±4.57 |  | **ASD symptoms** (SRS, CARS, and CGI) | CARS change, mean (sd) = -5.35 (3.88); SRS change, mean (sd) = − 21.83 (19.78); CGI (Very much improved) change, mean (sd) = 1 |
|  |  | Placebo | 23 |  |  | 82.6 | 8.87±4.96 |  | **ASD symptoms** (SRS, CARS, and CGI) | CARS change, mean (sd) = -1.79 (2.39); SRS change, mean (sd) − 1.55 (20.38); CGI (Very much improved) change, mean (sd) = 0 |
| ***Sprengers 2021*** | RCT | Bumetanide | 47 | None | ASD | 68.1 | 10.5±2.5 | None | **ASD symptoms (**SRS-2) | After 91 days, bumetanide was not superior to placebo on the primary outcome, the SRS-2 (mean difference 3.16, 95% CI= 9.68 to 3.37, p = .338). A superior effect was found on one of the secondary outcomes, the RBS (mean difference 4.16, 95% CI 8.06 to 0.25, p.0375), but not on the Sensory Profile (mean difference 5.64, 95% CI = 11.30 to 22.57, p .508) or the ABC (mean difference 0.65, 95% CI 2.83 to 1.52, p= .552) |
|  |  | Placebo | 45 |  |  | 68.9 | 10.25±2.4 |  | **ASD symptoms** (SRS-2) |  |
| ***Zhang***  ***2020*** | RCT | Placebo | 41 | None | ASD | 71 | 3.97±1.01 | None | **ASD symptoms** (CARS, CGI, and exploratory neurotransmitter concentrations measured by MRS) | Compared with the control group: the bumetanide group showed significant reduction in symptom severity, as indicated by both total CARS score and number of items assigned a score ≥ 3. The improvement in clinical symptoms was confirmed by CGI. GABA/Glx ratio in both the IC and VC decreased more rapidly over the 3-month period, with the symptom improvement in the bumetanide group. |
|  |  | Bumetanide | 42 |  |  | 86 | 4.19±0.95 |  | **ASD symptoms** (CARS, CGI, and exploratory neurotransmitter concentrations measured by MRS) |  |
| ***Lemmonier 2010*** | RCT | Bumetanide | 60 | None | ASD | 80 | range: 3-11 | None | **ASD symptoms** (ABC, CGI, CARS, RDEG and RRB) | Significant improvement in infantile autistic syndrome with no side effects. |

*Median (25th-75th percentiles)

6-MWT: 6-minute walk test; ABA: applied behavior analysis; ABC: Aberrant Behaviour Checklist; ABCIS: Aberrant Behavior Checklist Irritability Subscale; ACEIs: Angiotensin-converting enzyme inhibitors; ADHD-RS IV: ADHD Rating Scale IV; ADHD: attention deficit hyperactivity disorder; ADHDRS: ADHD Rating Scale; ADOS: Autism Diagnostic Observation Schedule; AHRS: Hofmann's Auditory Hallucinations Rating Scale; APRS: Academic Performance Rating Scale; ARBs: Angiotensin II Receptor Blockers; ASQ-P: Aberrant Behavior Checklist Irritability and Hyperactivity subscales, Conner’s Abbreviated Symptom; BAI: Beck Anxiety Inventory; BARS: Barnes Akathisia Rating Scale, BD: Bipolar Disorder; BDI-II: Beck Depression Inventory-II; BDI: Back Depression Inventory; BEQ: Berkeley Expressivity Questionnaire; BFI-44: Big Five Inventory; BPRS: Brief Psychiatric Rating Scale; BRIEF-P: Behavioural Rating Inventory of Executive Function; CAD: Coronary Artery Disease; CAPS: Clinician Administered PTSD Scale for DSM-IV; CARS: Children Autism Rating Scale; CCBs: Calcium Channel Blockers; CDSS: Calgary Depression Scale for Schizophrenia; CFT: Rey-Osterrieth Complex Figure Test; CGAS: Children's Global Assessment Scale; CGI-I: Clinical Global Impressions of Improvement; CGI-S: Clinical Global Impressions of Severity of Illness; COWAT: Cognitive measures of attention included the Stroop and Controlled Oral Word Association Test; CPT: Continuous Performance Test; CRA: Compound Remote Associates test; CYBOCS: Children's Yale-Brown Obsessive-Compulsive Scale; DBS: Disruptive Behavior Scale; DSST: Digit symbol substitution test; FC: functional connectivity; GAD: generalized anxiety disorder; GDS: Gordon Diagnostic System; GP: grooved pegboard; GPB: Grooved Pegboard; GXR: Guanfacine extended-release; HAM-A: Hamilton Anxiety Scale; HAMD: Hamilton Rating Scale for Depression; HDRS: Hamilton Rating Scale for Depression; HFrEF: heart failure with reduced ejection fraction; HPPD: Hallucinogen persisting perception disorder; HPPD: LSD-induced Hallucinogen Persisting Perception Disorder; HSC: Hopkins Symptom Check-list; HSQ SSQ: Home Situations Questionnaire and School Situations Questionnaire; IES-R: Impact of Event Scale-Revised; MCCB: MATRICS Consensus Cognitive Battery; MDD: major depressive disorder; MMSE: Mini Mental State Examination; MOVES: Motor tic, Obsessions and compulsions, Vocal tic Evaluation Survey; MRS: magnetic resonance spectroscopy; NA: Not Available; OCD: Obsessive-Compulsive Disorder; OCDS: Obsessive-Compulsive Drinking Scale; ODD: oppositional defiant disorder; OLE: Open-Label Extension study; OTSRD: other trauma- and stress-related disorders; PANSS: Positive and Negative Syndrome Scale; PDD: Pervasive Developmental Disorders; PMDD: Premenstrual dysphoric disorder; PRISM: Prospective record of the impact and severity of menstrual symptoms; PSQI: Pittsburgh Sleep Quality Index; PSYRATS: Psychotic Symptom Rating Scales; PTSD: Post-traumatic stress disorder; PVAQ: Pain Vigilance and Awarness Questionnaire; RAVLT: Rey Auditory Verbal Learning Test; RBS-R: Repetitive Behavior Scale Revised; RCT: Randomized Controlled Trial; RDEG: Regulation Disorders Evaluation Grid Questionnaire–Parent; RRB: Repetitive and Restricted Behaviour scale; SAD: social anxiety disorder; SANS: Scale for the Assessment of Negative Symptoms; SAS: Simpson Angus Scale for extra-pyramidal side effects; SCL-90: Symptom Checklist 90 Revised; SIG: Self-Injury Grid; SIT: Self-Injury Trauma; SPMSQ: Short Portable Mental Status Questionnaire; SSRI: Selective Serotonin Reuptake Inhibitors; SRS: Social Responsive Scale; TAS-20: Toronto Alexithymia Scale; TEMPS-A: Temperament Evaluation of Memphis, Pisa, Paris and San Diego, Autoquestionnaire; UCLA-PSTDRI: The University of California at Los Angeles Post-traumatic Stress Disorder Reaction Index; UPSA: UCSD Performace Skills Assessment; WASI: Wechsler Abbreviated Scale of Intelligence; WFIRS-P: Weiss Functional Impairment Rating Scale-Parent Report; Y-BOCS: Yale-Brown Obsessive-Compulsive scale.

**SUPPLEMENTARY REFERENCES (not reported in the Main text)**

Ahmadpanah, M., Sabzeiee, P., Hosseini, S.M., Torabian, S., Haghighi, M., Jahangard, L., Bajoghli, H., Holsboer-Trachsler, E., Brand, S., 2014. Comparing the effect of prazosin and hydroxyzine on sleep quality in patients suffering from posttraumatic stress disorder. Neuropsychobiology 69, 235–242. https://doi.org/10.1159/000362243

Biederman, J., Melmed, R.D., Patel, A., McBurnett, K., Donahue, J., Lyne, A., 2008. Long-term, open-label extension study of guanfacine extended release in children and adolescents with ADHD. CNS Spectr. 13, 1047–1055. https://doi.org/10.1017/S1092852900017107

Biederman, Joseph, Melmed, R.D., Patel, A., McBurnett, K., Konow, J., Lyne, A., Scherer, N., 2008. A randomized, double-blind, placebo-controlled study of guanfacine extended release in children and adolescents with attention-deficit/hyperactivity disorder. Pediatrics 121, e73–e84. <https://doi.org/10.1542/peds.2006-3695>

Bilder, R.M., Loo, S.K., McGough, J.J., Whelan, F., Hellemann, G., Sugar, C., Del’Homme, M., Sturm, A., Cowen, J., Hanada, G., McCracken, J.T., 2016. Cognitive Effects of Stimulant, Guanfacine, and Combined Treatment in Child and Adolescent Attention-Deficit/Hyperactivity Disorder. J. Am. Acad. Child Adolesc. Psychiatry 55, 667–673. https://doi.org/10.1016/j.jaac.2016.05.016

Byers, M.G., Allison, K.M., Wendel, C.S., Lee, J.K., 2010. Prazosin versus quetiapine for nighttime posttraumatic stress disorder symptoms in veterans: An assessment of long-term comparative effectiveness and safety. J. Clin. Psychopharmacol. 30, 225–229. <https://doi.org/10.1097/JCP.0b013e3181dac52f>

Braszko JJ, Karwowska-Polecka W, Halicka D, Gard PR. Captopril And Enalapril Improve Cognition And Depressed Mood In Hypertensive Patients. J Basic Clin Physiol Pharmacol J Basic Clin Physiol Pharmacol; 2003;14:323–344.

Bunevicius, R., Hinderliter, A.L., Light, K.C., Pedersen, C.A., Girdler, S.S., 2005. Lack of beneficial effects of clonidine in the treatment of premenstrual dysphoric disorder: Results of a double-blind, randomized study. Hum. Psychopharmacol. 20, 33–39. https://doi.org/10.1002/hup.652

Calohan, J., Peterson, K., Peskind, E.R., Raskind, M.A., 2010. Prazosin treatment of trauma nightmares and sleep disturbance in soldiers deployed in Iraq. J. Trauma. Stress 23, 645–648. https://doi.org/10.1002/jts.20570

Capone, G.T., Brecher, L., Bay, M., 2016. Guanfacine Use in Children with Down Syndrome and Comorbid Attention-Deficit Hyperactivity Disorder (ADHD) with Disruptive Behaviors. J. Child Neurol. 31, 957–964. https://doi.org/10.1177/0883073816634854

Connor, D.F., Barkley, R.A., Davis, H.T., 2000. A pilot study of methylphenidate, clonidine, or the combination in ADHD comorbid with aggressive oppositional defiant or conduct disorder. Clin. Pediatr. (Phila). 39, 15–25. https://doi.org/10.1177/000992280003900102

Cutler, A.J., Brams, M., Bukstein, O., Mattingly, G., McBurnett, K., White, C., Rubin, J., 2014. Response/remission with guanfacine extended-release and psychostimulants in children and adolescents with attention-deficit/hyperactivity disorder. J. Am. Acad. Child Adolesc. Psychiatry 53, 1092–1101. https://doi.org/10.1016/j.jaac.2014.08.001

Dereli S, Kılınçel O, Çerik İB, Kaya A. Impact of sacubitril/valsartan treatment on depression and anxiety in heart failure with reduced ejection fraction. Acta Cardiol Acta Cardiol; 2020;75:774–782.

Diokpa, C., Backe, K., Pinsonnault, J., 2021. A retrospective chart review to determine the safety and efficacy of prazosin for nightmares related to posttraumatic stress disorder in veterans. Hum. Psychopharmacol. 36. <https://doi.org/10.1002/hup.2785>

Dikici S, Kocaman G, Ozdem S, Kocer A. Amlodipine-induced delirium in a patient with ischemic stroke. Neurologist Neurologist; 2012;18:171–172.

Ferrafiat, V., Soleimani, M., Chaumette, B., Martinez, A., Guilé, J.M., Keeshin, B., Gerardin, P., 2020. Use of Prazosin for Pediatric Post-Traumatic Stress Disorder With Nightmares and/or Sleep Disorder: Case Series of 18 Patients Prospectively Assessed. Front. Psychiatry 11. https://doi.org/10.3389/fpsyt.2020.00724

Friedman, J.I., Adler, D.N., Temporini, H.D., Kemether, E., Harvey, P.D., White, L., Parrella, M., Davis, K.L., 2001. Guanfacine treatment of cognitive impairment in schizophrenia. Neuropsychopharmacology 25, 402–409. https://doi.org/10.1016/S0893-133X(01)00249-4

Gaffney, G.R., Perry, P.J., Lund, B.C., Bever-Stille, K.A., Arndt, S., Kuperman, S., 2002. Risperidone Versus Clonidine in the Treatment of Children and Adolescents with Tourette’s Syndrome. J. Am. Acad. Child Adolesc. Psychiatry 41, 330–336. https://doi.org/10.1097/00004583-200203000-00013

Germain, A., Richardson, R., Moul, D.E., Mammen, O., Haas, G., Forman, S.D., Rode, N., Begley, A., Nofzinger, E.A., 2012. Placebo-controlled comparison of prazosin and cognitive-behavioral treatments for sleep disturbances in US Military Veterans. J. Psychosom. Res. 72, 89–96. https://doi.org/10.1016/j.jpsychores.2011.11.010

Hazell, P.L., Stuart, J.E., 2003. A randomized controlled trial of clonidine added to psychostimulant medication for hyperactive and aggressive children. J. Am. Acad. Child Adolesc. Psychiatry 42, 886–894. https://doi.org/10.1097/01.CHI.0000046908.27264.00

Hervas, A., Huss, M., Johnson, M., McNicholas, F., van Stralen, J., Sreckovic, S., Lyne, A., Bloomfield, R., Sikirica, V., Robertson, B., 2014. Efficacy and safety of extended-release guanfacine hydrochloride in children and adolescents with attention-deficit/hyperactivity disorder: A randomized, controlled, Phase III trial. Eur. Neuropsychopharmacol. 24, 1861–1872. <https://doi.org/10.1016/j.euroneuro.2014.09.014>

Hu WS, Lin CL. Association between angiotensin-converting enzyme inhibitors, angiotensin receptor blockers, and major psychiatric disorders. J Affect Disord Elsevier; 2021;289:16–20.

Iwanami, A., Saito, K., Fujiwara, M., Okutsu, D., Ichikawa, H., 2020. Efficacy and safety of guanfacine extended-release in the treatment of attention-deficit/hyperactivity disorder in adults: Results of a randomized, double-blind, placebo-controlled study. J. Clin. Psychiatry 81, 7891. https://doi.org/10.4088/JCP.19m12979

Jain, R., Segal, S., Kollins, S.H., Khayrallah, M., 2011. Clonidine extended-release tablets for pediatric patients with attention-deficit/hyperactivity disorder. J. Am. Acad. Child Adolesc. Psychiatry 50, 171–179. https://doi.org/10.1016/j.jaac.2010.11.005

Kollins, S.H., Jain, R., Brams, M., Segal, S., Findling, R.L., Wigal, S.B., Khayrallah, M., 2011a. Clonidine extended-release tablets as add-on therapy to psychostimulants in children and adolescents with ADHD. Pediatrics 127. https://doi.org/10.1542/peds.2010-1260

Kollins, S.H., López, F.A., Vince, B.D., Turnbow, J.M., Farrand, K., Lyne, A., Wigal, S.B., Roth, T., 2011b. Psychomotor functioning and alertness with guanfacine extended release in subjects with attention-deficit/hyperactivity disorder. J. Child Adolesc. Psychopharmacol. 21, 111–120. https://doi.org/10.1089/cap.2010.0064

Kurlan, R., Goetz, C.G., McDermott, M.P., Plumb, S., Singer, H., Dure, L., Como, P., Sallee, F.R., Budman, C., Coffey, B., Juncos, J., Mink, J., Stebbins, G., Tuite, P., Seeberger, L., Pelham, W.E., Palumbo, D., Giuliano, J., Krieger, M., Lane, J., Pearson, N., Sine, L., Parsons, K., Peters, S., Thorne-Petrizzi, D., Parks, K., Kim, G., Craddock, K., Wood, C., Randle, J., Janko, K., Lasher, D., Johnston, T., Bean, S., Riddle, M., Leckman, J.F., Oakes, D., Richard, I., Sehgal, N., Hogarth, P., Marcus, D., Kieburtz, K., Harris, P., Sulkes, S., Cox, C., LaDonna, D., Brower, C., Greiner, A., 2002. Treatment of ADHD in children with tics: A randomized controlled trial. Neurology 58, 527–536. https://doi.org/10.1212/WNL.58.4.527

Loo, S.K., Bilder, R.M., Cho, A.L., Sturm, A., Cowen, J., Walshaw, P., Levitt, J., Del’Homme, M., Piacentini, J., McGough, J.J., McCracken, J.T., 2016. Effects of d-Methylphenidate, Guanfacine, and Their Combination on Electroencephalogram Resting State Spectral Power in Attention-Deficit/Hyperactivity Disorder. J. Am. Acad. Child Adolesc. Psychiatry 55, 674-682.e1. https://doi.org/10.1016/j.jaac.2016.04.020

McCracken, J.T., McGough, J.J., Loo, S.K., Levitt, J., Del’Homme, M., Cowen, J., Sturm, A., Whelan, F., Hellemann, G., Sugar, C., Bilder, R.M., 2016. Combined Stimulant and Guanfacine Administration in Attention-Deficit/Hyperactivity Disorder: A Controlled, Comparative Study. J. Am. Acad. Child Adolesc. Psychiatry 55, 657-666.e1. https://doi.org/10.1016/j.jaac.2016.05.015

Okazaki, K., Yamamuro, K., Iida, J., Kishimoto, T., 2019. Guanfacine monotherapy for ADHD/ASD comorbid with Tourette syndrome: A case report. Ann. Gen. Psychiatry 18, 1–5. https://doi.org/10.1186/S12991-019-0226-6/FIGURES/1

Palumbo, D.R., Sallee, F.R., Pelham, W.E., Bukstein, O.G., Daviss, W.B., McDermott, M.P., Burrows-MacLean, L., Como, P., Hoffman, M.T., Lock, T.M., Nelson, D., McConville, B., Janciuras, J., Bean, S.A., Raab, D., Arnold, F., Kipp, H., Pearson, N., Vierhile, A., Russell, B., Tresco, K., Cannon, M., Maher, S., Marcus, D., Riggs, G., Robb, A., Harris, P., Richard, I., Sulkes, S., Christopher Cox, Kurlan, R., Conners, K., Hunt, R., Kieburtz, K., De Blieck, E., Lindsay, P., Preston, L., Rothenburgh, K., Julian-Baros, E., Orme, C., Kamp, D., Thompson, L., 2008. Clonidine for attention-deficit/hyperactivity disorder: I. Efficacy and tolerability outcomes. J. Am. Acad. Child Adolesc. Psychiatry 47, 180–188. <https://doi.org/10.1097/chi.0b013e31815d9af7>

Koola, M.M., Varghese, S.P., Fawcett, J.A., 2014. High-dose prazosin for the treatment of post-traumatic stress disorder. Ther. Adv. Psychopharmacol. https://doi.org/10.1177/2045125313500982

Racin, R., Bellonci, C., Coffey, B.J., 2014. Expanded usage of prazosin in pre-pubertal children with nightmares resulting from posttraumatic stress disorder. J. Child Adolesc. Psychopharmacol. <https://doi.org/10.1089/cap.2014.2482>

Sallee, F.R., McGough, J., Wigal, T., Donahue, J., Lyne, A., Biederman, J., 2009. Guanfacine extended release in children and adolescents with attention-deficit/hyperactivity disorder: A placebo-controlled trial. J. Am. Acad. Child Adolesc. Psychiatry 48, 155–165. <https://doi.org/10.1097/CHI.0b013e318191769e>

Scahill, L., Aman, M.G., McDougle, C.J., McCracken, J.T., Tierney, E., Dziura, J., Arnold, L.E., Posey, D., Young, C., Shah, B., Ghuman, J., Ritz, L., Vitiello, B., Ramadan, Y., Witwer, A., Lindsay, R., Swiezy, N., Kohn, A., Cronin, P., McGough, J., Lee, L.S.Y., Martin, A., Koenig, K., Carroll, D., Lancor, A., Gonzalez, N.M., Grados, M., Chuang, S., Davies, M., Robinson, J., McMahon, D., 2006. A prospective open trial of guanfacine in children with pervasive developmental disorders. J. Child Adolesc. Psychopharmacol. 16, 589–598. https://doi.org/10.1089/cap.2006.16.589

Scahill, L., Chappell, P.B., Kim, Y.S., Schultz, R.T., Katsovich, L., Shepherd, E., Arnsten, A.F.T., Cohen, D.J., Leckman, J.F., 2001. A placebo-controlled study of guanfacine in the treatment of children with tic disorders and attention deficit hyperactivity disorder. Am. J. Psychiatry 158, 1067–1074. https://doi.org/10.1176/appi.ajp.158.7.1067

Scahill, L., McCracken, J.T., King, B.H., Rockhill, C., Shah, B., Politte, L., Sanders, R., Minjarez, M., Cowen, J., Mullett, J., Page, C., Ward, D., Deng, Y., Loo, S., Dziura, J., McDougle, C.J., 2015. Extended-release guanfacine for hyperactivity in children with autism spectrum disorder. Am. J. Psychiatry 172, 1197–1206. https://doi.org/10.1176/appi.ajp.2015.15010055

Spencer, T.J., Greenbaum, M., Ginsberg, L.D., Murphy, W.R., 2009. Safety and effectiveness of coadministration of guanfacine extended release and psychostimulants in children and adolescents with attention-deficit/ hyperactivity disorder. J. Child Adolesc. Psychopharmacol. 19, 501–510. https://doi.org/10.1089/cap.2008.0152

Srivastava, S.K., Nath, C., 2000. The differential effects of calcium channel blockers in the behavioural despair test in mice. Pharmacol. Res. 42, 293–297. https://doi.org/10.1006/phrs.2000.0696

Stein, M.A., Sikirica, V., Weiss, M.D., Robertson, B., Lyne, A., Newcorn, J.H., 2015. Does Guanfacine Extended Release Impact Functional Impairment in Children with Attention-Deficit/Hyperactivity Disorder? Results from a Randomized Controlled Trial. CNS Drugs 29, 953–962. https://doi.org/10.1007/s40263-015-0291-6

Symons, F.J., Thompson, A., Realmuto, G., 2004. Clonidine for self-injurious behavior [4]. J. Am. Acad. Child Adolesc. Psychiatry. https://doi.org/10.1097/01.chi.0000138565.72531.75

Taormina, S.P., Galloway, M.P., Rosenberg, D.R., 2016. Treatment Efficacy of Combined Sertraline and Guanfacine in Comorbid Obsessive-Compulsive Disorder and Attention Deficit/Hyperactivity Disorder: Two Case Studies. J. Dev. Behav. Pediatr. 37, 491–495. <https://doi.org/10.1097/DBP.0000000000000290>

Tarlow MM, Sakaris A, Scoyni R, Wolf-Klein G. Quinapril-associated acute psychosis in an older woman [1]. J. Am. Geriatr. Soc. John Wiley & Sons, Ltd; 2000. p. 1533.

Taylor, F.B., Russo, J., 2001. Comparing guanfacine and dextroamphetamine for the treatment of adult attention-deficit/hyperactivity disorder. J. Clin. Psychopharmacol. 21, 223–228. https://doi.org/10.1097/00004714-200104000-00015

van Stralen, J.P.M., 2020. A Controlled Trial of Extended-Release Guanfacine and Psychostimulants on Executive Function and ADHD. J. Atten. Disord. 24, 318–325. https://doi.org/10.1177/1087054717751197

Wilens, T.E., McBurnett, K., Turnbow, J., Rugino, T., White, C., Youcha, S., 2017. Morning and Evening Effects of Guanfacine Extended Release Adjunctive to Psychostimulants in Pediatric ADHD: Results From a Phase III Multicenter Trial. J. Atten. Disord. 21, 110–119. https://doi.org/10.1177/1087054713500144

Yadav, S., Takács, A., Pantelis, C., Thomas, N., 2021. Clonidine induced sexual disinhibition in a patient with treatment-resistant schizophrenia: A case report. Aust. N. Z. J. Psychiatry. https://doi.org/10.1177/0004867420945788
